# Mapping the global health burden of climate-sensitive exposures: a systematic scoping review

**DOI:** 10.1186/s12940-026-01294-8

**Published:** 2026-04-02

**Authors:** Julia Feriato Corvetto, Robin Simion, Perla Boutros, Nour Kassem, Kristine Belesova, Till Bärnighausen, Rainer Sauerborn, Sandra Barteit

**Affiliations:** 1 Heidelberg Institute of Global Health (HIGH), Faculty of Medicine and University Hospital, Heidelberg University, Heidelberg, Germany; 2 Graduate Program on Biomedical Engineering, Federal University of Technology - Paraná, Curitiba, Brazil; 3 Department of Primary Care and Public Health, School of Public Health, Imperial College, London, UK; 4 Africa Health Research Institute (AHRI), Somkhele, KwaZulu-Natal South Africa; 5 Harvard Center for Population and Development Studies, T.H. Chan School of Public Health, Cambridge, MA USA

**Keywords:** Climate change, Climate-sensitive exposures, Global health, Scoping review, Environmental health, Disease burden, Adaptation policy

## Abstract

**Background:**

Climate change is an increasing determinant of morbidity and mortality worldwide. However, the health burden attributable to climate-sensitive exposures remains poorly quantified and inconsistently reported across the existing literature. This lack of systematic evidence limits the ability of policymakers and health systems to allocate resources efficiently, design targeted interventions, and implement effective adaptation strategies.

**Methods:**

We conducted a systematic scoping review following the PRISMA-ScR guidelines to map and synthesize available global evidence on the health impacts of climate-sensitive hazards, including heat, temperature variability, extreme weather events, and air pollution. Peer-reviewed studies published up to November 2024 were identified through searches in Scopus, PubMed, Embase, and Web of Science. Eligible studies were charted by exposure type, health outcome, study design, and geographic region. The review protocol was pre-registered in PROSPERO (CRD42023421873).

**Results:**

Of the 15,538 records screened, 199 studies met the inclusion criteria. Evidence was most consistent for heat exposure, which showed strong associations with all-cause, cardiovascular, and respiratory mortality. Across 15 disease categories—including mental health, infectious, endocrine, and neurological disorders—exposures sensitive to climate change were associated with increased morbidity and mortality risks. However, the available evidence base was heavily skewed toward high- and upper-middle-income countries, with over half of the studies conducted in China. Evidence gaps were identified for undernutrition, injuries, disabilities, and non-fatal outcomes, particularly in low- and middle-income countries. Heterogeneity in exposure definitions, outcome, and regional data availability limited comparability across studies. A selective meta-analysis of comparable studies yielded pooled attributable fractions of 1.18% (95% CI: 1.01–1.37) for all-cause, 2.15% (1.54–2.88) for cardiovascular, 3.08% (2.17–4.15) for respiratory, and 2.71% (1.85–3.73) for stroke-related mortality due to heat. Additional climate-sensitive exposures and outcomes were not comparable and therefore could not be pooled.

**Conclusions:**

Climate-sensitive exposures are associated with a substantial health burden as reflected in the available global literature, with the strongest evidence originating from high-resource settings. Findings from this scoping review highlight the urgent need to enhance climate health surveillance systems in underrepresented regions, harmonize exposure and outcome metrics, and strengthen the integration of health evidence into national adaptation strategies and climate finance mechanisms. Addressing these evidence and infrastructure gaps is critical for informing equitable, data-driven adaptation planning, public health preparedness, and downstream loss-and-damage policy discussions.

**Supplementary Information:**

The online version contains supplementary material available at 10.1186/s12940-026-01294-8.

## Background

The health impacts of climate change are increasingly recognized at the global level, with a growing body of evidence linking climate-sensitive hazards—such as extreme heat, flooding, and storms—to elevated health risks [1, 2]. Notably, all ten of the leading global causes of death and disease burden, including ischemic heart disease, stroke, and chronic obstructive pulmonary disease (COPD), are sensitive to environmental conditions and may be influenced by climate-sensitive exposures [3, 4]. As both chronic disease prevalence and the intensity of climate hazards increase, the number of people affected is expected to rise as a consequence. However, the population-level health burden attributable to these exposures remains poorly quantified [5, 6], limiting our ability to assess their full impact on morbidity and mortality.

Although the Global Burden of Disease (GBD) framework has recently begun incorporating certain environmental risk factors—such as non-optimal ambient temperature—it does not yet provide comprehensive estimates of the burden associated with the broader spectrum of climate-sensitive exposures [7]. This represents a critical gap in global health surveillance and limits the evidence base available to guide adaptation policies and public health planning. While some recent studies have estimated the proportion of health outcomes attributable to specific climate-sensitive exposures, the literature remains methodologically diverse and fragmented.

Quantifying the health burden of climate hazards is essential for informing public health priorities, guiding equitable resource allocation, and evaluating climate-related economic losses. These estimates are also increasingly relevant for international negotiations and mechanisms such as the “loss and damage” fund formalized at COP28 (UNFCCC Conference of Parties), which aims to support vulnerable countries affected by climate change impacts.

Stern (2006) emphasized that health damages constitute a significant share of the economic costs associated with climate change, highlighting the importance of accurately quantifying their burden [8]. As climate-sensitive hazards intensify and chronic health conditions rise, the cumulative strain on public health systems is expected to increase. In response, burden of disease metrics such as the attributable fraction (AF) and attributable number (AN) have become increasingly common tools for estimating the proportion and absolute number of health outcomes linked to specific climate exposures. These metrics support adaptation planning by translating exposure-risk relationships into actionable public health estimates.

Additional burden metrics—including years of life lost (YLL), years lived with disability (YLD), and disability-adjusted life years (DALYs)—capture both mortality and morbidity impacts and are widely used in global health assessments. However, the application of these composite metrics to climate-sensitive health impacts remains limited, particularly in low-resource settings, due to challenges such as data scarcity, limited resolution of health statistics, and insufficient exposure-response functions. In contrast, AF and AN metrics are more feasible in many contexts because they require fewer data inputs, often rely on established relative risks from the literature, and are relevant for acute or short-term outcomes [9, 10]. Since 2018, these methods have gained prominence in estimating the health impacts of climate hazards—such as heatwaves, floods, and droughts—by linking environmental exposures to outcomes like emergency department visits, hospitalizations, and premature deaths. As the frequency and intensity of these events increase with climate change, population-level burdens are expected to rise even if individual-level risks remain constant.

Previous reviews have advanced our understanding of the health impacts of climate-sensitive exposures but continue to highlight substantial evidence gaps. For instance, Cheng et al. (2019) conducted a global review of studies published up to 2018, applying burden of disease metrics such as attributable fraction (AF) and years lived with disability (YLD) to assess health impacts linked to non-optimal temperatures [11]. Their findings showed that temperature-related exposures contributed significantly to mortality, with over 2.5% of deaths in high-income countries and more than 3% in middle-income countries attributed to non-optimal temperatures. However, their analysis was limited in scope: it excluded other climate-sensitive exposures such as air pollution and extreme weather events, and it aggregated results by country income level, without a deeper and more detailed resolution by disease category or population subgroup. These limitations constrained the identification of specific vulnerabilities and hindered targeted public health response. In a more recent assessment, the 2024 Wellcome Trust report proposed a conceptual framework for attributing human health outcomes to anthropogenic climate change, using rigorous detection and attribution methods [12]. While this represents an important step forward, the report also identified major gaps in the available literature. Of nearly 4,000 studies screened, only 13 met the criteria for robust attribution, and most focused narrowly on heat-related mortality. Other key health outcomes—such as infectious diseases, mental health, and non-communicable conditions—were largely absent.

The lack of a comprehensive synthesis on climate-sensitive exposures limits the ability to draw generalizable conclusions and constrains the evidence base required for effective public health planning. To address this gap, the present scoping review systematically identifies and maps existing studies reporting health outcomes attributable to major climate-sensitive hazards (e.g., heat, floods, storms, air pollution). AF is used as a standardized burden metric to facilitate comparison across studies and exposures. This review focuses on summarizing the distribution, scope, and methodological characteristics of existing AF-based evidence. In doing so, it identifies areas of emerging consistency and highlights persistent knowledge gaps. By charting exposure–outcome relationships across settings and health domains, the review seeks to support priority-setting for climate-health research and inform the design of adaptation strategies tailored to the most vulnerable populations.

## Methods

This scoping review was conducted following the PRISMA-ScR (Preferred Reporting Items for Systematic Reviews and Meta-Analyses extension for Scoping Reviews; see Supporting Information for the PRISMA Checklist – Appendix) [13] and was pre-registered in PROSPERO (CRD42023421873, registered on April 30, 2023) [14]. The search strategy was structured according to the PECO framework [15]: the population encompassed all human populations without subgroup restriction; exposures included all climate-sensitive exposures identified by the Intergovernmental Panel on Climate Change (IPCC, 2023) [16] being influenced or intensified by climate change, excluding cold exposure, as the global frequency and severity of cold extremes are projected to decrease [17]; the comparison group referred to populations experiencing no, lower, or baseline levels of these exposures; and outcomes were defined as morbidity and mortality estimates reported using AF or related burden measures.

A systematic and comprehensive search was conducted across multiple databases to identify studies reporting quantitative estimates of health outcomes linked to climate-sensitive exposures. Search terms were adapted for each database and combined using Boolean operators. Three clusters of terms were applied:

1. Climate-sensitive exposures: climate change, extreme weather, heat, storms, drought, floods, precipitation, sea level, air pollution.
2. Health outcomes: mortality, diseases, death, burden, morbidity, transmission, prevalence, incidence, emergency department visit, hospital admission.
3. Effect measures: attributable fraction, attributable risk, attributable proportion.

This review focused on exposures whose magnitude or frequency is intensified by anthropogenic climate change. Although cold-related mortality remains substantial globally, cold extremes have decreased in frequency and severity under current warming trends. Therefore, cold-related attributable fractions were considered outside the conceptual scope of this review. Extreme weather events (including floods, storms, and droughts) were grouped under a broader “extreme events” category due to the limited number of studies available for each specific hazard type. This grouping was intended to provide a descriptive overview rather than event-specific pooled estimates.

Database-specific syntax and Boolean logic are detailed in Supplementary Files S2 and S3.

### Study selection

We conducted a comprehensive literature search across four databases—PubMed, Embase, Web of Science, and Scopus—for studies published up to November 26, 2024. Duplicate records were removed using EndNote reference management software, and the remaining entries were screened using Rayyan, a collaborative screening platform [18, 19]. Screening was performed by a team of seven independent reviewers (JFC, RSi, PB, NK, RR, MA, and KB), with each record independently assessed by two reviewers. In the initial screening phase, titles and abstracts were retained if selected by at least one of the two reviewers. For the full-text review, inclusion required agreement between both reviewers; in cases of disagreement, a third independent reviewer resolved the decision. The eligibility criteria for inclusion and exclusion are detailed in Table 1. For studies meeting inclusion criteria but lacking critical data—such as 95% confidence intervals or population size—corresponding authors were contacted and given a 10-day window to respond. Studies with unresolved data gaps after this period were excluded from the analysis. As this review follows PRISMA-ScR guidance for scoping reviews, we did not conduct a formal risk-of-bias or quality appraisal using standardized tools, which are not routinely recommended for scoping reviews. Instead, a data completeness and methodological eligibility check was applied to ensure extractability and interpretability of AF estimates. Studies were excluded at this stage if they lacked extractable AF values, confidence intervals, population denominators, or sufficient methodological description to support descriptive or quantitative synthesis.

**Table 1:** Inclusion and exclusion criteria. The three groups are ‘literature and population’, ‘exposures’ and ‘outcomes’

Inclusion criteria	Exclusion criteria
Literature and Population	
- Peer-reviewed and original articles. - All countries. - Age and sex groups with no limitation. - Languages: English.	-Full text not available. -Non-peer reviewed articles, conference presentations, opinion papers, reviews. -Methodology not clear (no methods described, no AF calculation methods reported). -No AF values presented in extractable format. -No given confidence interval or number of events in the studied period (e.g., deaths, hospitalization). -Grey literature (books, government reports, interviews, etc.). -Studies related to other biological species.
Exposures	
- Variables considered to be consequences of climate change or climate change-sensitive: heat, heatwaves, sea level rising, air pollution, storms, floods, droughts and extreme precipitation.	- Risk attribution under the modelling of future CC scenarios. - Cold spells, geologic events (volcanoes, earthquakes). - Non-climate related air pollution: cigarette and second-hand smoking, indoors pollution, evaluation of air quality exclusively around roads.
Outcomes	
- Articles that primarily analyse the attributable fraction of climate-change-sensitive diseases. - All health areas. - Morbidity (emergency department visits, hospital admissions, transmission rate, prevalence, etc.) and mortality.	- No outcome groups were excluded from the analysis.

Studies modelling future climate scenarios were excluded because they do not report AFs based on observed exposures and are methodologically incompatible with historical observational AF estimates. Exclusions are acknowledged as limitations in the Discussion. Ambient air pollution was included as a climate-sensitive exposure, based on the last IPCC report, due to its partial sensitivity to meteorological conditions—such as air temperature, stagnation events, and photochemical reactions—while acknowledging that it also has substantial non-climatic anthropogenic sources. Our inclusion reflects the dual nature of air pollution as both a co-exposure and an impact-modifying factor in climate-health relationships.

### Data extraction

Extracted data included: authors, year, country, study period, population size (total and subgroups), study design, statistical model, health outcome (morbidity or mortality), ICD-10 diagnoses (International Classification of Diseases – 10th revision) [20], AF, and attributable number (AN). In the overall descriptive analysis resulting from all included studies, results were synthesized by exposure (heat, temperature variability, extreme events, and air pollution) and outcome (ICD-10 subgroups, e.g., A00-B99 – infectious diseases). For pre–post intervention studies, only pre-intervention data were extracted to maintain comparability across observational studies and focus on unmitigated exposure–outcome relationships.

### Additional quantitative synthesis approach

In response to the substantial heterogeneity identified across study designs, exposure definitions, and outcome metrics, we did not conduct a universal meta-analysis across all climate-sensitive exposure–outcome pairs, as such pooling would yield non-interpretable and potentially misleading estimates. However, to enhance quantitative interpretability, we performed selective random-effects meta-analyses for exposure–outcome pairs with methodological comparability (heat exposure and all-cause, cardiovascular, respiratory, and stroke mortality), using DerSimonian-Laird models. Attributable fractions were transformed using logit methods, and pooled estimates were calculated using inverse-variance weighting. Heterogeneity was assessed with I² and τ² statistics. Meta-analyses were limited to heat-related mortality outcomes due to methodological diversity across other exposure–outcome categories. For all other categories, where heterogeneity precluded valid pooling, we conducted a quantitative descriptive synthesis, reporting medians, interquartile ranges, and stratified summaries (by exposure type, region, and outcome group). This combined approach allows quantitative consolidation where appropriate while maintaining methodological rigor.

To assess methodological heterogeneity in AF calculations, we systematically extracted and compared key methodological dimensions across studies, including:

1. counterfactual definitions (e.g., theoretical minimum risk exposure level, observed minimum exposure, regulatory standards),
2. assumptions regarding exposure distribution,
3. baseline risk definitions, and
4. whether AF estimates were derived from model-based or empirical risk estimates.

For quantitative synthesis, comparability of counterfactual scenarios was treated as a prerequisite for pooling. Studies employing non-equivalent counterfactual definitions were not combined within the same meta-analysis but were analysed separately.

### Role of the funding source

This study did not receive any direct external funding. Kristine Belesova is affiliated with the NIHR Centre for Non-Communicable Diseases and Environmental Change (NIHR203247).

## Results

**Fig. 1:**
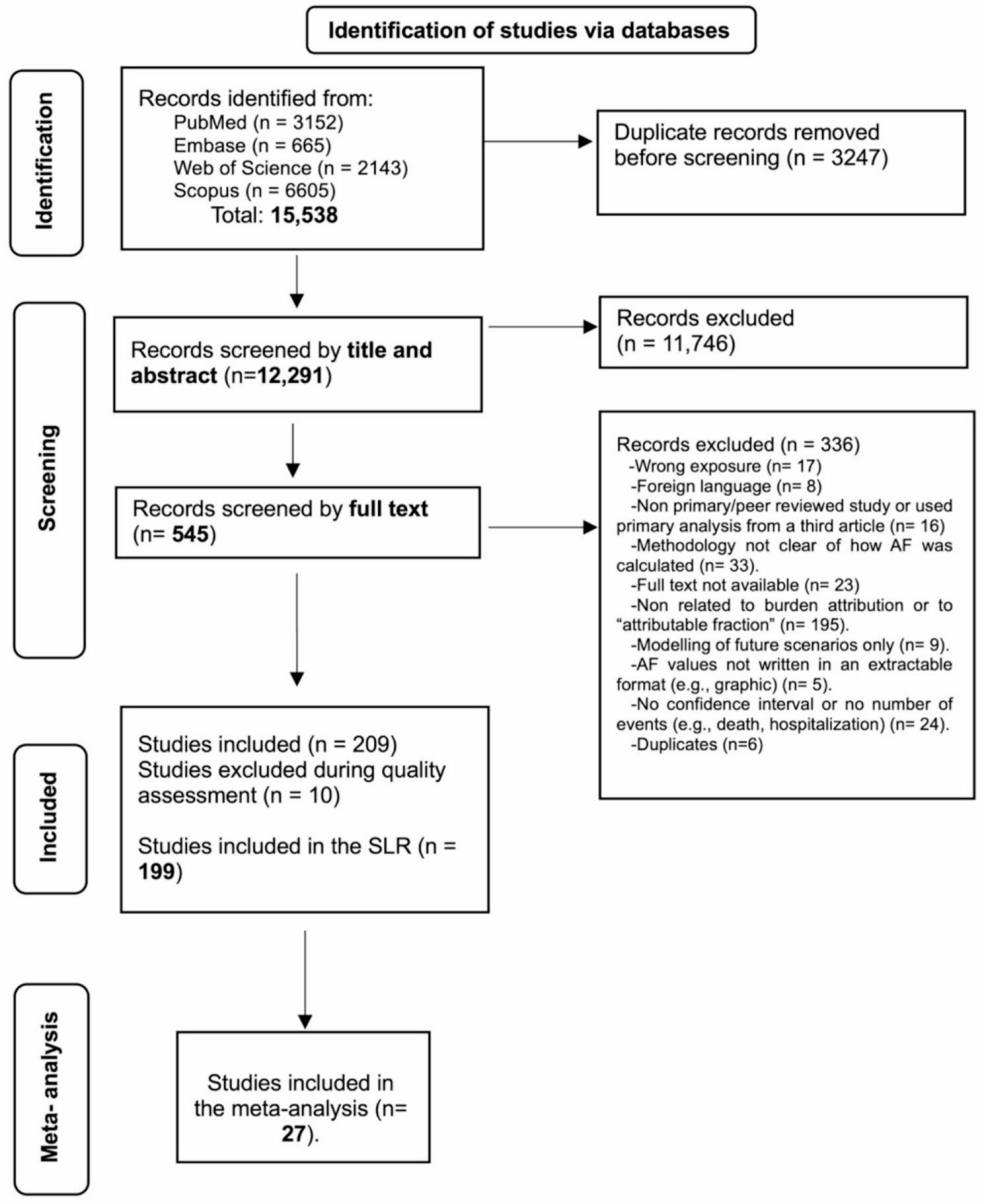
PRISMA flowchart

From 15,538 identified studies, 12,291 were screened by title and abstract, and 545 by full text. After excluding 329 records and removing 17 studies during a data completeness and methodological eligibility check, 199 studies remained eligible for inclusion (Fig. 1). Table 2 summarizes characteristics of the 199 included studies. Most were published after 2020 (69%) and conducted in high- and upper-middle-income countries, with heat (65%) and air pollution (27%) being the most frequently assessed exposures. Morbidity outcomes (57%) were more commonly studied than mortality, with cardiovascular and respiratory diseases comprising the largest disease categories. Detailed tables of included/excluded literature and quality assessment results are in the Appendix.

**Table 2:** Descriptive results of all 199 included studies

Date	No. of studies (%)
≤ 2010	1 (0.5%)
2011-2015	3 (1.5%)
2016-2020	57 (29%)
2021-2025	138 (69%)

GBD super regions	No. of country-level attributions
High-income	242
Sub-Saharan Africa	107
Southeast Asia, East Asia, and Oceania	193
North Africa and Middle East	29
Latin America and Caribbean	128
Central Europe, Eastern Europe, and Central Asia	35
South Asia	20

World Bank income levels	Country-level attributions
High	315
Upper-middle income	267
Lower-middle income	119
Low income	54

Study methodology	No. of studies (%)
Quantitative	199 (100%)

Study design	No. of studies (%)
Ecological time-series	164 (82.5%)
Time-stratified case crossover	29 (14.5%)
Cohort	3 (1.5%)
Case-control	2 (1%)
Quasi-experimental research design	1 (0.5%)

Exposure	No. of studies (%)
Heat	129 (65%)
Air pollution	54 (27%)
Temperature variability	22 (11%)
Extreme weather events	19 (10%)

Outcome	No. of studies (%)
Morbidity	113 (57%)
Mortality	90 (45%)
All-cause	63 (32%)
Infectious diseases (ICD-10 A00-B99)	15 (7.5%)
Neoplasm (C00-D48)	6 (3%)
Nutritional anemias (D50–D53)	1 (0.5%)
Endocrine, nutritional and metabolic (E00-E99)	10 (5%)
Mental health (F00-F99)	21 (10.5%)
Neurological (G00-G99)	13 (6.5%)
Cardiovascular (I00-I99)	53 (27%)
Respiratory (J00-J99)	59 (30%)
Diseases of the Digestive System (K00-K93)	2 (1%)
Skin (L00-L99)	3 (1.5%)
Musculoskeletal (M00-M99)	5 (2.5%)
Genitourinary (N00-N99)	13 (6.5%)
Pregnancy, childbirth and puerperium (O00-O9A)	4 (2%)
Occupational-related outcomes	5 (2.5%)
Injury-related outcomes	4 (2%)

**Fig. 2:**
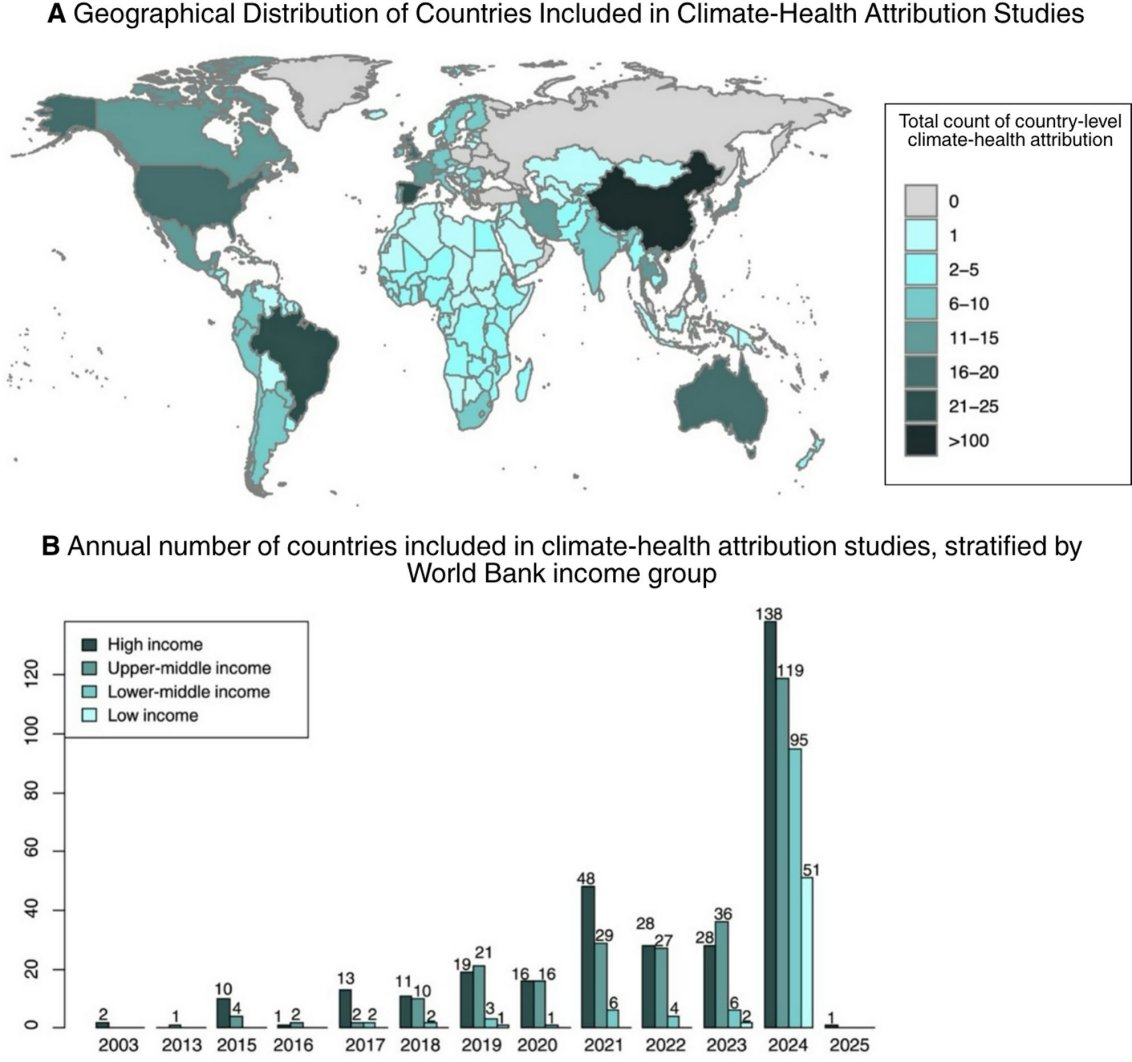
**A** Geographical distribution of country-level climate-health attributions of all included studies; darker shades indicate higher study concentration. **B** Annual number of countries included in climate-health attribution studies, stratified by World Bank income group [24]

The included literature spans 157 countries, representing a broad geographic and socioeconomic distribution: 25 low-income, 45 lower-middle-income, 39 upper-middle-income, and 48 high-income countries (Fig. 2A). China accounted for the largest number of studies (*n* = 116), followed by Brazil (*n* = 24) and Spain (*n* = 20). Publication volume has increased steadily over time, with a marked acceleration beginning in 2023 (Fig. 2B). Notably, the year 2024 shows broader representation from low- and lower-middle-income countries, as well as climate-vulnerable regions such as sub-Saharan Africa, South and Southeast Asia, and Latin America and the Caribbean—largely driven by multi-country study designs. Health outcomes related to morbidity (*n* = 113) were more frequently investigated than mortality (*n* = 90), with hospital admissions comprising the most common morbidity metric (*n* = 68). The vast majority of studies utilized administrative healthcare data (e.g., hospital or emergency department records), while only three studies relied on population-based survey data, specifically from the Demographic and Health Surveys (DHS) program [21–23]. This reflects a bias toward populations with health system access and limits insights into health burdens among those without regular access to care.

**Fig. 3:**
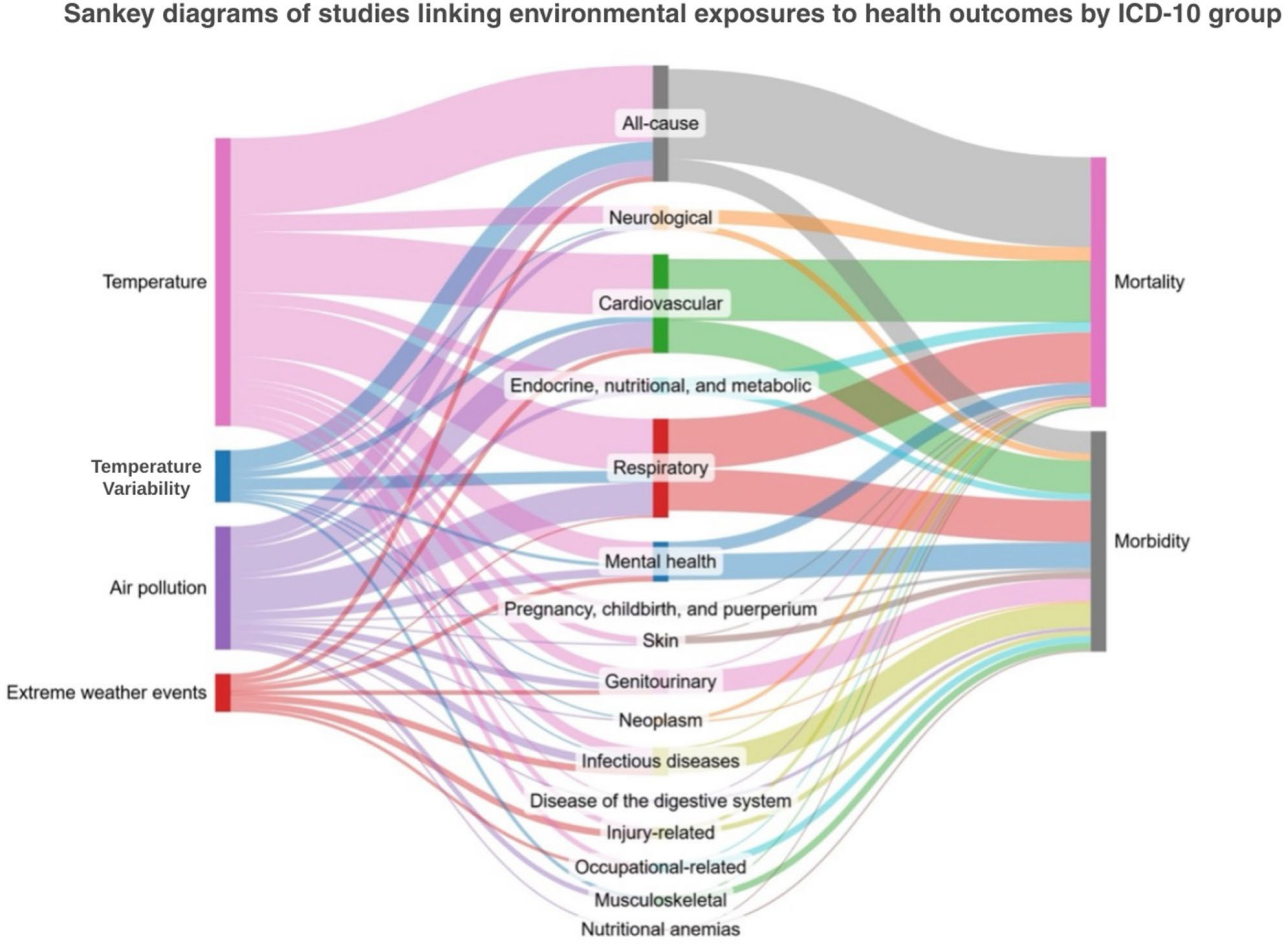
Sankey diagram showing the number of studies linking temperature, temperature variability, air pollution, and extreme weather events to health outcomes by ICD-10 group. The diagram (Fig. 3) illustrates the distribution of evidence across exposure–outcome pairings rather than the magnitude of attributable fractions. They highlight a pronounced clustering of studies on heat-related cardiovascular and respiratory outcomes, alongside substantial evidence gaps for injuries, undernutrition, disability, and several non-communicable disease categories—particularly in relation to extreme weather events

Figure 3 illustrates the frequency of each climate-sensitive exposure, disease category, and outcome (morbidity and mortality) analyzed across studies. This review encompassed 15 disease categories based on ICD-10 classifications, with the majority of studies focusing on respiratory (J00–J99; *n* = 59) and cardiovascular conditions (I00–I99; *n* = 53), particularly cerebrovascular diseases (I60–I69). Other health outcomes included infectious diseases (A00–B99), neoplasms (C00–D48), nutritional anemias (D50–D53), endocrine, nutritional, and metabolic disorders (E00–E99), mental and behavioral disorders (F00–F99), neurological conditions (G00–G99), digestive diseases (K00–K93), skin diseases (L00–L99), musculoskeletal disorders (M00–M99), genitourinary diseases (N00–N99), pregnancy and childbirth-related outcomes (O00–O9A), and outcomes related to occupational and injury-related exposures.

Temperature-related exposures were the most frequently examined (*n* = 129), although exposure definitions varied across studies. Common definitions included “heat,” typically operationalized as mean temperature (Tmean) above the minimum mortality temperature (MMT; *n* = 49), and “extreme heat,” defined as Tmean at or above the 97.5th percentile (*n* = 22). These definitions are used consistently throughout this review. Additional temperature metrics included Tmean values between the MMT and the 95th or 97.5th percentiles. Other exposures assessed included air pollution (*n* = 54), temperature variability—both intra-day (e.g., diurnal temperature range) and inter-day changes (*n* = 22)—and extreme weather events such as floods, storms, and droughts (*n* = 19).

### Selective meta-analysis of heat-related mortality

**Fig. 4:**
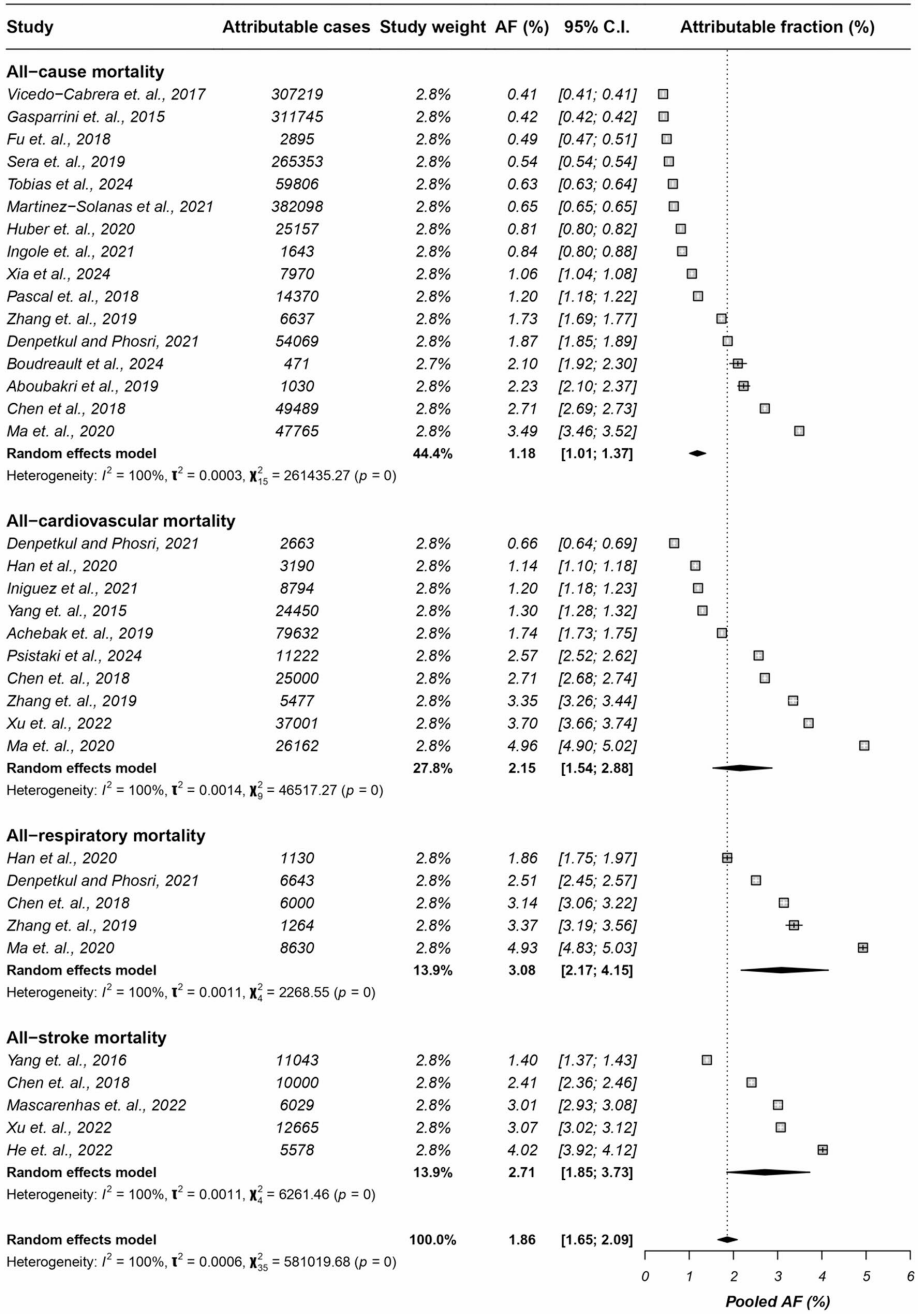
Random-effects meta-analyses of studies reporting AFs for mortality associated with heat exposure, stratified by all-cause, cardiovascular, respiratory, and stroke mortality. Pooled estimates are shown as diamonds, with individual study estimates and 95% CIs. High between-study heterogeneity (I² > 95%) reflects variation in exposure definitions, study populations, and analytical approaches. Estimates should be interpreted with caution

We conducted a selective meta-analysis for exposure–outcome pairs with sufficient methodological comparability. Pooled estimates were generated only for heat exposure and mortality outcomes (all-cause, cardiovascular, respiratory, and stroke), using random-effects models. As anticipated, heterogeneity was high across all pooled outcomes, reflecting variation in exposure thresholds, temperature metrics, population characteristics, and statistical modelling approaches. Therefore, pooled values should be interpreted cautiously as indicative summaries of the available evidence, rather than precise or globally representative burden estimates. For all-cause mortality, the pooled AF from 16 eligible studies was 1.18% (95% CI: 1.01–1.37) with substantial heterogeneity (I² = 100%). For cardiovascular mortality, 10 studies yielded a pooled AF of 2.15% (95% CI: 1.54–2.88) (I² = 100%). For respiratory mortality, 5 studies indicated a pooled AF of 3.08% (95% CI: 2.17–4.15) (I² = 100%). For stroke mortality, 5 studies produced a pooled AF of 2.71% (95% CI: 1.85–3.73) (I² = 100%). A forest plot summarizing these pooled estimates is provided in Fig. 4.

### Characterization of climate-sensitive exposure metrics

The included literature applied twelve distinct definitions of heat exposure, reflecting variation in threshold, duration, and metric type. Common definitions included (1) mean temperature (Tₘₑₐₙ) at or above the minimum mortality temperature (MMT), (2) Tₘₑₐₙ at or above the 95th percentile, (3) Tₘₑₐₙ at or above the 97.5th percentile, (4) maximum temperature (Tₘₐₓ) at or above the 95th percentile, (5) Tₘₐₓ at or above the 97.5th percentile, (6) minimum temperature (Tₘ_i_ₙ) at or above the 95th percentile, (7) Tₘ_i_ₙ at or above the 97.5th percentile, (8) fixed absolute thresholds (e.g., Tₘₑₐₙ ≥ 25 °C), (9) heatwave definitions based on duration and intensity (e.g., ≥ 3 days above threshold), (10) apparent temperature (heat-index) thresholds, (11) wet-bulb globe temperature (WBGT) thresholds, and (12) definitions based on the excess heat factor (EHF). Less frequently used metrics—including exposures above median temperature and variants of the apparent temperature index and urban heat island effects—are detailed in the Appendix [25–29].

### Mortality burden from climate-sensitive exposures

**Fig. 5:**
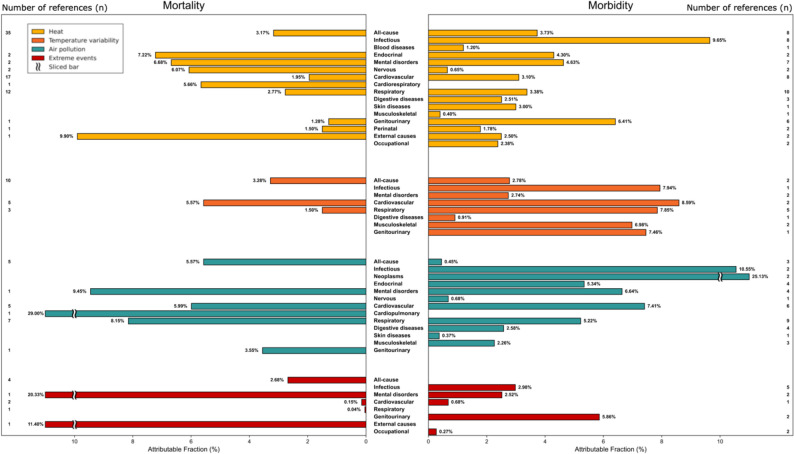
Horizontal bar charts compare the attributable fraction (AF, %) of mortality (left) and morbidity (right) due to heat (temperature over the minimum mortality temperature), temperature variability (intraday and interday variability), air pollution, and extreme events (including heatwaves, floods, extreme precipitation and tropical cyclones), stratified by the ICD-10 health outcome categories. Numbers at the edge of each bar indicate the number of studies included for that outcome (number of references). For visibility purposes, bars larger then 10% were sliced, as indicated in the figure

Exposure to heat was consistently associated with increased mortality risks (Fig. 5, Table 1a – Appendix). Heat exposure accounted for 3.17% (95% CI: 0.80–5.53) [21–23, 25, 26, 30–57] of all-cause mortality, 1.95% (95% CI: 1.14–2.75) [25, 36, 43, 44, 57–69] of cardiovascular deaths, and 2.77% (95% CI: 1.19–4.35) [25, 36, 55, 57–59, 62, 70] of respiratory-related deaths. Mortality associated with mental disorders showed a highly uncertain AF estimate of 6.68% (95% CI: − 21.09–34.44) [27, 44], based on a very small number of contributing studies, and a particularly strong association for suicides at 9.90% (95% CI: 9.40–10.40) [71], based on single-country evidence. Temperature variability (TV) also demonstrated significant associations with mortality, with attributable fractions (AFs) of 5.57% (95% CI: 0.22–11.26) [57, 72–74] for cardiovascular diseases and 1.50% (95% CI: − 0.66–3.66) [57, 72–74] for respiratory diseases. TV further contributed to all-cause mortality at 3.28% (95% CI: 0.11–6.35) [57, 72–80], highlighting widespread health impacts due to temperature fluctuations.

Ambient air pollution showed robust associations with increased mortality, with AFs of 5.57% (95% CI: − 4.15–15.29) [81–85] for all-cause mortality, 9.45% (95% CI: − 3.02–21.91) [86] for mental disorders, including dementia, 8.15% (95% CI: − 6.29–22.19) [58, 83–85, 87–89] for respiratory diseases, and 5.99% (95% CI: − 5.74–17.72) [58, 83–85, 88] for cardiovascular diseases.

Extreme weather events notably influenced mortality risk, particularly for mental disorders, with an AF of 20.33% (95% CI: 18.32–23.39) [90]. All-cause mortality attributable to extreme events was 2.68% (95% CI: − 5.02–10.38) [91–94]. External causes, especially drowning, exhibited strong associations with extreme events, showing an AF of 11.40% (95% CI: 10.00–12.90) [95].

### Morbidity burden from climate-sensitive exposures

Climate-sensitive exposures significantly contributed to morbidity (Fig. 5, Table 1b – Appendix). Heat exposure was associated with an AF of 3.73% (95% CI: 1.82–5.64) [42, 96–103] for non-specific outcomes and 9.65% (95% CI: − 1.55–20.86) [101, 103–109] for infectious diseases. Respiratory diseases and genitourinary diseases, including acute kidney injuries, demonstrated particularly strong associations, with AFs of 3.38% (95% CI: 0.89–5.88) [28, 62, 69, 101, 104, 110–112] and 6.41% (95% CI: 1.00–11.81) [101, 103, 113–116], respectively.

Temperature variability also substantially contributed to morbidity, notably affecting cardiovascular outcomes with an AF of 8.59% (95% CI: 1.09–16.09) [99, 117] and respiratory outcomes with an AF of 7.85% (95% CI: − 1.34–17.03) [82, 118–120]. Extreme weather events, including heatwaves and extreme precipitation, accounted for nearly 3% (95% CI: −0.94–6.90) [93, 121–124] of infectious disease morbidity. Combined exposures (temperature and air pollution) further elevated health risks, with two studies reporting AFs of 5.31% (95% CI: 4.58–5.91) [125] and 16.65% (95% CI: 16.43–16.87) [88]. Finally, estimates derived specifically from warm-season data may exaggerate year-round risks and can be found in the Appendix (Table 1b). This methodological concern, highlighted in multiple studies, emphasizes the need for cautious interpretation when applying seasonal AFs to annual morbidity burdens.

Morbidity outcomes varied substantially in severity and temporal profile, including acute hospital admissions, exacerbations of chronic disease, and subclinical or symptom-based endpoints. This heterogeneity limits direct comparison across morbidity AF estimates. Although analysed as a single category, included studies varied substantially in hazard characteristics. Floods typically represent acute, localized exposure events; storms may involve both immediate injury pathways and longer-term displacement effects; droughts often represent slow-onset exposures affecting food security and chronic health outcomes. This heterogeneity limits direct comparability of AF estimates across event types. In general, substantial heterogeneity was observed in AF calculation approaches, particularly regarding counterfactual definitions and baseline risk assumptions. Studies employing comparable counterfactual standards were pooled, while those using alternative counterfactual frameworks were analysed separately to avoid methodological conflation.

## Discussion

This review maps available global evidence on disease burdens attributable to climate-sensitive exposures, revealing significant health impacts across varied settings and outcomes. Estimates from the broader descriptive synthesis suggest that heat exposure accounts for approximately 1.18% of all-cause mortality - equivalent to around 800,000 deaths annually.

Higher attributable fractions (AFs) are observed for specific health outcomes: cardiovascular mortality (~ 2.15%), respiratory mortality (~ 3.08%), and stroke-related mortality (~ 2.71%). Temperature variability and air pollution are also consistently linked to elevated morbidity and mortality risks. Drawing on 199 studies spanning 157 countries and covering 15 distinct disease subgroups, our findings highlight both the breadth of climate-sensitive health risks and the severity of their impact. By estimating disease-specific AFs, this work addresses a crucial gap in the climate-health evidence base and builds on—but also advances beyond—existing reviews. To enhance the interpretability of the findings, we conducted a selective meta-analysis for heat–mortality pairs with comparable definitions and methodological quality, producing pooled AFs that complement the broader descriptive synthesis.

### Interpretation and context

Previous syntheses—such as Cheng et al. (2019) [11], Berrang-Ford et al. (2021) [2], the Wellcome Open Research report (2024) [12]—have each contributed foundational insights. Cheng et al. [11] provided country-level estimates of temperature-related burden using DALYs and YLDs, but they were limited to non-optimal temperatures and lacked other climate-sensitive exposures, estimating that more than 2.5% of mortality in high-income countries and over 3% in middle-income countries was due to temperature exposure. However, their analysis predated the surge of literature post-2018, which we were able to capture. Our review incorporates 199 studies (197 published since 2018), encompassing 15 disease subgroups and exposures beyond temperature, such as air pollution and extreme weather events, thereby offering a more granular and policy-relevant picture.

The Wellcome Trust review emphasized the nascent state of formal attribution science and identified a severe lack of studies quantifying health outcomes attributable specifically to anthropogenic climate change [12]. Only 13 such studies were found among nearly 4,000 screened. Our review, by contrast, systematically quantifies climate-attributable burdens using AFs, demonstrating that heat exposure alone is responsible for 1.18% of all-cause mortality (~ 800,000 deaths annually), with higher burdens for highly prevalent conditions, such as cardiovascular, respiratory, and stroke mortality. These estimates offer the empirical foundation the Wellcome report called for, suitable for use in health sector planning and climate compensation frameworks.

Berrang-Ford et al. (2021) [2] flagged key methodological challenges—such as lack of standardisation and concentration of studies in high-income countries—and mapped the available detection and attribution literature [2]. Our review addresses these concerns by applying a uniform metric (AF) across exposures and outcomes and by showing emerging representation from low- and lower-middle-income countries in recent years (notably 2023–2024). While their approach was exploratory, our work provides a structured and detailed evidence map that supports targeted climate-health research and adaptation planning.

An important question arising from this expanded evidence base is whether it alters prior conclusions regarding the relative importance of different climate-sensitive health outcomes. Overall, our findings suggest that the broad ranking of climate-sensitive exposures remains consistent with earlier reviews, with heat-related cardiovascular and respiratory outcomes continuing to dominate the available evidence, particularly for mortality. However, the substantially enlarged post-2018 literature extends this hierarchy by revealing emerging and increasingly consistent burdens in domains that were previously underrepresented, including mental health, renal disease (e.g., acute kidney injury), infectious diseases, and occupational outcomes. Importantly, the apparent dominance of heat-related mortality reflects a strong concentration of research effort rather than definitive evidence that heat constitutes the largest comparative health burden across all climate-sensitive exposures. This distinction—between evidence density and true burden—was less explicit in earlier syntheses and underscores the need to interpret apparent risk hierarchies in light of underlying research patterns.

Landscape. (2020) reported wide variability in heat-related AFs (0.04–10.5%) but focused solely on mortality and did not offer pooled estimates. We extend their work by providing pooled global AFs not only for mortality but also morbidity (e.g., 13.09% for tuberculosis hospitalizations linked to temperature variability), thereby expanding the burden landscape.

#### Sources of heterogeneity

The substantial heterogeneity observed across studies reflects systematic differences in study design, exposure metrics, regional climate conditions, and outcome definitions. Ecological time-series studies, which comprised the majority of the evidence base, reported sometimes lower AFs than case-crossover designs. AF estimates also varied meaningfully by exposure definition: studies using MMT thresholds generally produced lower AFs compatred with those using high percentile cut-offs (95th or 97.5th percentile). Regional patterns contributed further variability, with studies from tropical and subtropical climates reporting higher AFs than those from temperate regions. Unlike mortality outcomes, morbidity attributable fractions reflect a broader range of endpoints with differing severity, chronicity, and healthcare-seeking behavior. Acute outcomes such as emergency admissions may be more sensitive to short-term exposure fluctuations, whereas chronic conditions may reflect cumulative exposure. Furthermore, morbidity AF estimates may be influenced by healthcare access, reporting systems, and surveillance intensity, complicating cross-study comparability and public health interpretation.

Future syntheses examining total temperature-related burden should integrate both components.

Importantly, the distribution of attributable fraction (AF) studies across exposure–outcome pairs reflects research density rather than underlying burden magnitude. The dominance of heat-related mortality in the literature should not be interpreted as evidence that other climate-related health outcomes are of lesser importance. Instead, it highlights areas where AF methodology has been more frequently applied.

Consequently, morbidity attributable fraction (AF) estimates should not be interpreted solely based on their numerical magnitude, but rather in light of outcome severity, chronicity, and the broader health system context in which they arise. To enhance comparability and interpretability across settings, greater methodological harmonization is needed, including clearer consensus on counterfactual definitions, reference exposure values, and the integration of standardized approaches into study design from the outset.

### Limitations

This review has several limitations. First, the very high heterogeneity observed across studies reflects true variation in designs, exposure definitions, populations, and contexts, which limits direct comparability of findings. Second, there is a pronounced geographic bias: most data derive from high- and upper-middle-income countries, whereas evidence from highly vulnerable regions such as sub-Saharan Africa, Southeast Asia, and Latin America remains limited. Third, the reliance on healthcare utilization data likely underestimates the true morbidity burden in settings with limited access to care, particularly for non-fatal conditions among marginalized populations. This selection bias skews the perceived distribution of climate-sensitive health risks and highlights the need for expanded use of population-representative data sources, such as household surveys and community-based surveillance. Fourth, while many studies focus on heat exposure, extreme weather events remain relatively under-studied, indicating a meaningful gap in the literature. Fifth, although attributable fractions (AFs) provide intuitive estimates of the proportion of outcomes linked to specific exposures, they do not account for disease severity or duration, limiting comparability to integrated metrics such as disability-adjusted life years (DALYs). Additionally, AFs do not incorporate exposure prevalence the way population attributable fractions (PAFs) do; however, in contexts lacking exposure prevalence data, AFs still offer a foundational metric for mapping climate-health burdens. Finally, our estimates do not explicitly isolate the contribution of anthropogenic climate change; nonetheless, by translating observed exposure–response relationships into population-relevant metrics, these findings still hold value for health system planning under both current and future climate scenarios. The very high heterogeneity (I² > 95%) indicates that pooled AFs reflect broad central tendencies but cannot be interpreted as universal effect sizes. Differences in exposure metrics, population vulnerability, background climate, and model specifications introduce true effect variation that limits generalizability. Accordingly, pooled estimates should be interpreted as indicative summary measures rather than generalizable effect sizes, particularly given the substantial between-study heterogeneity.

While ambient air pollution is influenced by climate-sensitive meteorological conditions, its primary sources are often industrial, transportation-related, or agricultural. As such, the inclusion of air pollution may overestimate the fraction of health burden that is directly attributable to climate change. We included it given its partial climate sensitivity and widespread use in attribution literature but interpret these findings with appropriate caution. Aggregating heterogeneous extreme events may reduce interpretability of attributable fraction estimates, as exposure pathways, duration, and affected populations differ substantially across hazard types. The limited evidence base did not permit event-specific stratified synthesis. Future research should adopt more harmonized and hazard-specific exposure definitions to improve comparability. Outcomes with limited attributable fraction evidence, including several morbidity and extreme-event–related endpoints, likely represent methodological and research gaps rather than negligible public health impact. Variation in counterfactual definitions and exposure distributions limits direct comparability of AF estimates across studies. Differences in reference exposure levels, baseline risks, and modelling assumptions can substantially influence AF magnitude. Consequently, comparisons across studies should be interpreted with caution, particularly when counterfactual frameworks differ. Future research would benefit from greater harmonization of AF calculation standards in climate-health research. Excluding cold-related attributable fractions may limit completeness in representing total temperature-related mortality. However, because cold extremes are decreasing under current warming trends, inclusion would have conflated climate-amplified and climate-attenuated risks.

### Policy and research implications

Substantial investment in climate-sensitive health surveillance systems is needed, particularly in low-income and high-vulnerability settings where data gaps are most severe. National governments can operationalize the evidence summarized in this review by integrating attributable-fraction–based climate health indicators into National Adaptation Plans (NAPs), heat–health action plans, and public health preparedness strategies. Such integration would support prioritization of high-risk populations, facilitate monitoring of adaptation effectiveness, and strengthen alignment between health and climate policy domains.

WHO and other multilateral agencies are well positioned to advance methodological standardization by developing harmonized exposure definitions, outcome classifications, and reporting conventions for climate health attribution. Alignment with established frameworks such as the Global Burden of Disease (GBD) and IPCC assessment processes would enhance comparability across regions and improve the usability of attribution estimates for global monitoring, policy guidance, and climate finance mechanisms.

Research funders and academic institutions should prioritize sustained investment in longitudinal, population-based surveillance platforms—such as Health and Demographic Surveillance Systems (HDSS) and emerging climate health infrastructures like Climate Change and Health Evaluation and Response Systems (CHEERS) [126]—particularly in low- and middle-income countries. Expanding data sources beyond hospital and emergency department records is essential to capture non-fatal outcomes, marginalized populations, and climate-sensitive morbidity that remains underrepresented in administrative datasets. Together, improved data quality, geographic coverage, and methodological consistency are prerequisites for integrating health into adaptation planning, climate finance, and loss-and-damage frameworks. Taken together, these considerations define a three-axis research prioritization framework, spanning (i) geographic vulnerability, (ii) exposure complexity, and (iii) underrepresented outcome domains.

## Conclusion

In summary, this scoping review provides a synthesis of the available global evidence on health outcomes attributable to climate-sensitive exposures—including heat, temperature variability, extreme events, and ambient air pollution. By mapping the current literature, we identify consistent associations across multiple exposures and disease categories, while also revealing substantial gaps in geographic coverage, exposure types, and outcome domains. Although most studies focus on heat, this reflects a concentration of research effort rather than definitive comparative burden, underscoring the need to expand attention to underrepresented exposures such as extreme weather events, compound hazards, and temperature variability.

Furthermore, our findings highlight the importance of standardizing exposure metrics, enhancing climate health surveillance systems—particularly in low-resource settings—and strengthening the integration of health burden data into adaptation planning and climate finance frameworks. While attributable fractions do not replace DALYs or population attributable fractions, they offer a pragmatic starting point for estimating health burdens within data-constrained settings and evidence gaps. Quantifying these impacts, as reflected in the current literature, is essential for informing equitable public health responses, guiding research investments, and supporting downstream accountability and policy processes, including those related to climate-sensitive loss and damage.

## Supplementary Information

Supplementary Material 1.

## Data Availability

No datasets were generated or analysed during the current study.
