# Supplementary material for: Mapping the global health burden of climate-sensitive exposures: a systematic scoping review
Source: Environ Health. 2026 Apr 2;25:31. doi: 10.1186/s12940-026-01294-8 (PMC13063667; doi:10.1186/s12940-026-01294-8)
Supplement: Supplementary file 1 — Supplementary Material 1. [file 12940_2026_1294_MOESM1_ESM.docx]

Supplementary material

Quantifying the Global Health Burden of Climate-Sensitive Exposures: A Systematic Review and Meta-Analysis

Overview

[**S1. PRISMA Checklist.** 3](#_Toc203488332)

[S2. Search strings used for the research. 6](#_Toc203488333)

[S3. Detailed searching terms used and adapted according to the four databases. 6](#_Toc203488334)

[S4. Inclusion and exclusion criteria. 6](#_Toc203488335)

[S5. Risk of bias 7](#_Toc203488336)

[S6. Included studies 11](#_Toc203488337)

[S7. Excluded studies 83](#_Toc203488338)

[S8. Quality assessment 101](#_Toc203488339)

[Time series or case crossover 101](#_Toc203488340)

[Cohort and case-control studies 109](#_Toc203488341)

[Quasi-Experimental Studies 110](#_Toc203488342)

[S9. Narrative results: results synthesis of morbidity and mortality. 112](#_Toc203488343)

[S10. Sensitivity analysis Meta-Analysis. 112](#_Toc203488344)

[S11. Sensitivity analysis – meta-analysis performed after excluding outlying studies. 114](#_Toc203488345)

[S12. Funnel plots 117](#_Toc203488346)

[References of Appendix 118](#_Toc203488347)

| **S1. PRISMA Checklist.** | | | |
| --- | --- | --- | --- |
| **Section and Topic** | **Item #** | **Checklist item** | **Location where item is reported** |
| **TITLE** | | |  |
| Title | 1 | Identify the report as a systematic review. | √ Yes |
| **ABSTRACT** | | |  |
| Abstract | 2 | See the PRISMA 2020 for Abstracts checklist. | √ Yes |
| **INTRODUCTION** | | |  |
| Rationale | 3 | Describe the rationale for the review in the context of existing knowledge. | √ Page 3/4 |
| Objectives | 4 | Provide an explicit statement of the objective(s) or question(s) the review addresses. | √ Page 4 |
| **METHODS** | | |  |
| Eligibility criteria | 5 | Specify the inclusion and exclusion criteria for the review and how studies were grouped for the syntheses. | √ Page 4/5 |
| Information sources | 6 | Specify all databases, registers, websites, organisations, reference lists and other sources searched or consulted to identify studies. Specify the date when each source was last searched or consulted. | √ Page 5 |
| Search strategy | 7 | Present the full search strategies for all databases, registers and websites, including any filters and limits used. | √ Page 4/5 |
| Selection process | 8 | Specify the methods used to decide whether a study met the inclusion criteria of the review, including how many reviewers screened each record and each report retrieved, whether they worked independently, and if applicable, details of automation tools used in the process. | √ Page 5 |
| Data collection process | 9 | Specify the methods used to collect data from reports, including how many reviewers collected data from each report, whether they worked independently, any processes for obtaining or confirming data from study investigators, and if applicable, details of automation tools used in the process. | √ Page 5/6 |
| Data items | 10a | List and define all outcomes for which data were sought. Specify whether all results that were compatible with each outcome domain in each study were sought (e.g. for all measures, time points, analyses), and if not, the methods used to decide which results to collect. | √ Page 4/5 |
|  | 10b | List and define all other variables for which data were sought (e.g. participant and intervention characteristics, funding sources). Describe any assumptions made about any missing or unclear information. | √ Page 4/5 |
| Study risk of bias assessment | 11 | Specify the methods used to assess risk of bias in the included studies, including details of the tool(s) used, how many reviewers assessed each study and whether they worked independently, and if applicable, details of automation tools used in the process. | √ Page 6/7 (both quality assessment and risk of bias for meta-analyzed studies) |
| Effect measures | 12 | Specify for each outcome the effect measure(s) (e.g. risk ratio, mean difference) used in the synthesis or presentation of results. | √ Page 4/5 |
| Synthesis methods | 13a | Describe the processes used to decide which studies were eligible for each synthesis (e.g. tabulating the study intervention characteristics and comparing against the planned groups for each synthesis (item #5)). | √ Page 5/6 |
|  | 13b | Describe any methods required to prepare the data for presentation or synthesis, such as handling of missing summary statistics, or data conversions. | - |
|  | 13c | Describe any methods used to tabulate or visually display results of individual studies and syntheses. | √ Page 5/6 |
|  | 13d | Describe any methods used to synthesize results and provide a rationale for the choice(s). If meta-analysis was performed, describe the model(s), method(s) to identify the presence and extent of statistical heterogeneity, and software package(s) used. | √ Page 6 |
|  | 13e | Describe any methods used to explore possible causes of heterogeneity among study results (e.g. subgroup analysis, meta-regression). | - |
|  | 13f | Describe any sensitivity analyses conducted to assess robustness of the synthesized results. | √ Page 6 |
| Reporting bias assessment | 14 | Describe any methods used to assess risk of bias due to missing results in a synthesis (arising from reporting biases). | √ Page 6/7 |
| Certainty assessment | 15 | Describe any methods used to assess certainty (or confidence) in the body of evidence for an outcome. | √ Page 6/7 |
| **RESULTS** | | |  |
| Study selection | 16a | Describe the results of the search and selection process, from the number of records identified in the search to the number of studies included in the review, ideally using a flow diagram. | √ Figure 1 |
|  | 16b | Cite studies that might appear to meet the inclusion criteria, but which were excluded, and explain why they were excluded. | √ Table S6 |
| Study characteristics | 17 | Cite each included study and present its characteristics. | √ Table 1a, 1b, and S6, and Figure 2 |
| Risk of bias in studies | 18 | Present assessments of risk of bias for each included study. | √ S5 |
| Results of individual studies | 19 | For all outcomes, present, for each study: (a) summary statistics for each group (where appropriate) and (b) an effect estimate and its precision (e.g. confidence/credible interval), ideally using structured tables or plots. | √ Figure 2, 3a and 3b |
| Results of syntheses | 20a | For each synthesis, briefly summarise the characteristics and risk of bias among contributing studies. | √ Page 10/11 |
|  | 20b | Present results of all statistical syntheses conducted. If meta-analysis was done, present for each the summary estimate and its precision (e.g. confidence/credible interval) and measures of statistical heterogeneity. If comparing groups, describe the direction of the effect. | √ Page 10, 11, S10, S11 |
|  | 20c | Present results of all investigations of possible causes of heterogeneity among study results. | - |
|  | 20d | Present results of all sensitivity analyses conducted to assess the robustness of the synthesized results. | √ Page 10/11 and S10, S11, and S12 |
| Reporting biases | 21 | Present assessments of risk of bias due to missing results (arising from reporting biases) for each synthesis assessed. | - |
| Certainty of evidence | 22 | Present assessments of certainty (or confidence) in the body of evidence for each outcome assessed. | √ Page 10/11 |
| **DISCUSSION** | | |  |
| Discussion | 23a | Provide a general interpretation of the results in the context of other evidence. | √ Page 12/13 |
|  | 23b | Discuss any limitations of the evidence included in the review. | √ Page 14 |
|  | 23c | Discuss any limitations of the review processes used. | √ Page 14 |
|  | 23d | Discuss implications of the results for practice, policy, and future research. | √ Page 14/15 |
| **OTHER INFORMATION** | | |  |
| Registration and protocol | 24a | Provide registration information for the review, including register name and registration number, or state that the review was not registered. | √ Page 2 |
|  | 24b | Indicate where the review protocol can be accessed, or state that a protocol was not prepared. | √ Page 2 |
|  | 24c | Describe and explain any amendments to information provided at registration or in the protocol. | - |
| Support | 25 | Describe sources of financial or non-financial support for the review, and the role of the funders or sponsors in the review. | √ Page 15 |
| Competing interests | 26 | Declare any competing interests of review authors. | √ Page 15 |
| Availability of data, code and other materials | 27 | Report which of the following are publicly available and where they can be found: template data collection forms; data extracted from included studies; data used for all analyses; analytic code; any other materials used in the review. | √ Page 15 |

## S2. Search strings used for the research.

1. **CC variables**: Climate change, extreme weather, heat, storms, drought, floods, precipitation, sea level, air pollution.
2. **Health outcomes**: Mortality, diseases, death, burden, morbidity, transmission, prevalence, incidence, emergency department visit, hospital admission.
3. **Measure of effect**: Attributable fraction, attributable risk, attributable proportion.

## S3. Detailed searching terms used and adapted according to the four databases.

| **Database** | **Key words** |
| --- | --- |
| PubMed | ((climate change) **OR** (heat) **OR** (sea level) **OR** (storms) **OR** (drought) **OR** (floods) **OR** (precipitation) **OR** (extreme weather) **OR** (air pollution)) **AND** ((mortality) **OR** (diseases) **OR** (death) **OR** (burden) **OR** (morbidity) **OR** (transmission) **OR** (prevalence) **OR** (incidence) **OR** (hospital*) **OR** (emergency)) **AND** ((attributable fraction) **OR** (attributable risk) **OR** (attributable proportion)) |
| Web of Science | ((**ALL**=(climate change **OR** extreme weather **OR** heat* **OR** storm* **OR** drought* **OR** flood* **OR** rain* **OR** precipitation* **OR** air pollution* **OR** sea level*)) **AND** **ALL**=(mortalit* **OR** death* **OR** burden* **OR** morbidit* **OR** transmission* **OR** prevalance* **OR** incidence* **OR** hospital* **OR** emergenc*)) **AND** **ALL**=(attributable risk* **OR** attributable fraction* **OR** attributable proportion*) |
| Embase | ('climate change' **OR** 'extreme weather' **OR** heat **OR** storm **OR** flooding **OR** drought **OR** precipitation **OR** rain **OR** 'air pollution' **OR** 'sea level') **AND** (mortality **OR** death **OR** 'disease burden' **OR** morbidity **OR** transmission **OR** prevalence **OR** incidence **OR** 'hospital admission' **OR** hospitalization **OR** emergency) **AND** ('attributable risk' **OR** 'attributable fraction' **OR** 'attributable proportion') |
| Scopus | (“climate change” **OR** “extreme weather” **OR** heat OR storms **OR** drought **OR** floods **OR** rain* **OR** precipitation **OR** “air pollution” **OR** “sea level”) **AND** (mortality **OR** death **OR** burden **OR** morbidity **OR** transmission **OR** prevalence **OR** incidence **OR** hospital* **OR** emergency) **AND** (“attributable fraction” **OR** “attributable risk” **OR** “attributable proportion”) |

## S4. Inclusion and exclusion criteria.

| **Inclusion criteria** | **Exclusion criteria** |
| --- | --- |
| Literature and Population | |
| - Peer-reviewed and original articles.  - All countries.  - Age and sex groups with no limitation.  - Languages: English. | -Full text not available.  -Non-peer reviewed articles, conference presentations, opinion papers, reviews.  -Methodology not clear (no methods described, no AF calculation methods reported).  -No AF values presented in table format.  -No given confidence interval or number of events in the studied period (e.g., deaths, hospitalization).  -Grey literature (books, government reports, interviews, etc.).  -Studies related to other biological species. |
| Exposures | |
| - Variables considered to be consequences of climate change or climate change-sensitive: heat, heatwaves, sea level rising, air pollution, storms, floods, droughts and extreme precipitation. | - Risk attribution under the modelling of future CC scenarios.  - Cold spells, geologic events (volcanoes, earthquakes).  - Non-climate related air pollution: cigarette and second-hand smoking, indoors pollution, evaluation of air quality exclusively around roads. |
| Outcomes | |
| - Articles that primarily analyse the attributable fraction of CC-sensitive diseases.  - All health areas.  - Morbidity (emergency department visits, hospital admissions, transmission rate, prevalence, etc.) and mortality. | - No outcome groups were excluded from the analysis. |

Table 1. Inclusion and exclusion criteria. The three groups are ‘literature and population’, ‘exposures’ and ‘outcomes’.

## S5. Confidence in the body of evidence

| Outcome / design | Exposure (n of studies) | Start rating | Downgrade (reasons: (i) risk of bias, (ii) inconsistency, (iii) indirectness, (iv) imprecision, (v) publication bias) | Observations (downgrade) | Upgrade (reasons: (i) dose-response relationship, (ii) large effect sizes, (iii) apparent confounders were likely to reduce the effect) | Observations (upgrade) | Final rating: confidence in the body of evidence |
| --- | --- | --- | --- | --- | --- | --- | --- |
| All-cause mortality / time series | Heat: mean temperature > optimal temperature (16) | moderate | (-1) | (i) risk of bias: not significant, as only 3 studies received a grade of 3/5 (moderate) in the quality assessment/risk of bias, while the others graded at least 4/5. (ii) inconsistency: no.  (iii) indirectness: no. (iv) imprecision: no.  (v) publication bias: yes. Egger's test indicated funnel plot asymmetry, probably due to publication bias (p<0.0001). | (+1) | (i) dose-response relationship: yes. Studies suggested that high temperatures increase risk of mortality due to all-cause mortality.  (ii) large effect sizes: no.  (iii) apparent confounders were likely to reduce the effect: no. | moderate |
|  | Extreme heat: mean temperature ≥ 97.5th percentile (3) | moderate | (-1) | (i) risk of bias: no, all studies were graded at least 4/5. (ii) inconsistency: no. (iii) indirectness: no. (iv) imprecision: yes. Only three studies were included in this subgroup. Even though we consider this number as sufficient, we acknowledge that it is not the intended amount. (v) publication bias: Egger's test indicated no publication bias (p= 0.5121). | (+1) | (i) dose-response relationship: yes. Studies suggested that extreme high temperature increases risk of mortality.  (ii) large effect sizes: no.  (iii) apparent confounders were likely to reduce the effect: no. | moderate |
| All-cardiovascular mortality / time series | Heat: mean temperature > optimal temperature (10) | moderate | (-1) | (i) risk of bias: not significant, as only 1 study received a grade of 3/5 in the quality assessment/risk of bias, while the others graded at least 4/5.  (ii) inconsistency: yes. Effect size varies considerably across studies.  (iii) indirectness: no. (iv) imprecision: no.  (v) publication bias: no. Egger's test indicated no publication bias (p= 0.6927). | (+1) | (i) dose-response relationship: yes. Studies suggested that high temperatures increase risk of mortality due to all-cardiovascular mortality.  (ii) large effect sizes: no.  (iii) apparent confounders were likely to reduce the effect: no. | moderate |
| All-respiratory mortality / time series | Heat: mean temperature > optimal temperature (5) | moderate | (-1) | (i) risk of bias: no, all studies were graded at least 4/5.  (ii) inconsistency: yes. Effect size varies considerably across studies.  (iii) indirectness: no. (iv) imprecision: no.  (v) publication bias: no. Egger's test indicated no publication bias (p= 0.7116). | (+1) | (i) dose-response relationship: yes. Studies suggested that high temperatures increase risk of mortality due to all-respiratory mortality.  (ii) large effect sizes: no.  (iii) apparent confounders were likely to reduce the effect: no. | moderate |
| All-stroke mortality / time series | Heat: mean temperature > optimal temperature (5) | moderate | (-2) | (i) risk of bias: no, all studies were graded at least 4/5. (ii) inconsistency: yes. The effect size varies substantially across studies, without apparent reason or explanation. (iii) indirectness: no. (iv) imprecision: no.  (v) publication bias: yes. Egger's test indicated publication bias (p= 0.4679). | (+1) | (i) dose-response relationship: yes. Studies suggested that high temperatures increase risk of mortality due to all-respiratory mortality.  (ii) large effect sizes: no.  (iii) apparent confounders were likely to reduce the effect: no. Start | low (-1) |

## S6. Included studies

**Table 1.** Characteristics of included studies.

| Internal ID | Author, year | Country | Study period | Statistical model | Population count | Climate exposure; comparison measure | Outcome (ICD) | Mortality/Morbidity | Lags intervals used for AF measurement | AF % (CI) | Attributable numbers (CI) |
| --- | --- | --- | --- | --- | --- | --- | --- | --- | --- | --- | --- |
| 63 | Gasparrini et. al., 2015 | 384 locations, 13 countries | 28 years | TS (GLM + DLNM) | Total: 74,225,200 Australia: 1,177,95 Brazil: 3,401,136 Canada: 2,521,58 China: 950,130 Italy 820,390 Japan: 26,893,197 South Korea: 1,726,938 Spain: 3,479,910 Sweden: 190,090 Taiwan: 765,893 Thailand: 1,827,853 UK: 7,573,716 USA: 22,896,409 | Heat; mean temperature > MMT | Disorders in general | Mortality | 0-21 days | Total: 0.42 (0.39-0.44) Australia: 0.45 (0.20-0.70) Brazil: 0.70 (0.45-0.93) Canada: 0.54 (0.39-0.66) China: 0.64 (0.47-0.79) Italy: 1.62 (1.24-1.98) Japan: 0.32 (0.27-0.36) South Korea: 0.31 (0.15-0.45) Spain: 1.06 (0.96-1.16) Sweden: 0.18 (-47-0.65) Taiwan: 0.86 (0.12-1.50) Thailand: 0.76 (0.65-0.86) UK: 0.30 (0.25-0.36) USA: 0.35 (0.03-0.39) |  |
| 82 | Huber et. al., 2020 | 12 cities, Germany | 23 years | TS (GLM + DLNM) | 3,105,865 | Heat; mean temperature > MMT | Disorders in general | Mortality | 0-21 days | 0.81 (0.72-0.89) |  |
| 168 | Pascal et. al., 2018 | France | 11 years | TS (GLM + DLNM) | 1,197,555 | Heat; mean temperature > MMT | Disorders in general | Mortality | 0-21 days | 0-3 days: 1.2 (1.1-1.2)  0-21 days: 0.5 (0.4-0.6) | 0-3 days: 13,855 (13,388-14,403)  0-21 days: 5804 (4950-6619) |
| 83 | Ingole et. al., 2021 | Pune city, India | 9 years | TS (GLM + DLNM) | Total: 195,618 Men: 115,468 Women: 80,150 | Heat; mean temperature > MMT | Disorders in general | Mortality | 0-21 days | Total: 0.84 (0.35-1.30) Men: 0.84 (0.21-1.39) Women: 0.89 (0.14-1.57) |  |
| 148 | Martínez-Solanas et al., 2021 | 16 countries | 15 years | TS (DLNM) | 58,784,430 | Heat; mean temperature > MMT | Disorders in general | Mortality | 0-21 days | 0.65 (0.40-0.89) |  |
| 186 | Sera et. al., 2019 | 340 cities, 22 countries | 30 years | TS (DLNM) | 49,139,516 | Heat; mean temperature > MMT | Disorders in general | Mortality | 0-21 days | 0.54 (0.49- 0.58) | 265353 (240784, 285009) |
| 208 | Vicedo-Cabrera et. al., 2017 | 305 locations, 10 countries | 28 years | TS (DLNM) | 74,931,763 | Heat; mean temperature > MMT | Disorders in general | Mortality | 0-21 days | Total: 0.44 (0.43-0.44) Australia 0.48 (0.47-0.48) Brazil 0.67 (0.67-0.68) Canada 0.49 (0.49-0.50) Ireland 0.03 (0.03-0.03) Japan 0.44 (0.44-0.44) South Korea 0.34 (0.34-0.35) Spain 1.1 (1.1-1.11) Switzerland 1.28 (1.25-1.31) UK 0.32 (0.32-0.32) USA 0.37 (0.37-0.38) |  |
| 230 | Xu et. al., 2019 | China | 7 years | TS (DLNM) | 363,933 | Heat; mean temperature > MMT - Extreme heat; mean temperature > 97.5^th^ percentile | CVD (I00-I99) and respiratory diseases (J00-J99) | Mortality | 0-21 days | Total mortality: 1.65 (0.95- 2.36) Respiratory diseases: 3.09 (1.70- 4.24) CVD: 1.59 (0.73-2.38) | 6005 (3457, 8589) |
| 169 | Petkova et. al., 2021 | Bulgaria | 18 years | TS (DLNM) | 135,962 | Heat; mean temperature > MMT - Mild heat; MMT < mean temperature < 97.5^th^ percentile Extreme heat; mean temperature > 97.5^th^ percentile | Disorders in general | Mortality | 0-25 days | Heat: 1.1 (0.5-1.6) Mild heat: 0.8 (0.4-1.3) Extreme heat: 0.3 (0.1- 0.4) | Heat: 1495,582 (680, 2175) Mild heat: 1088 (544, 1768) Extreme heat: 408 (136, 544) |
| 4 | Achebak et. al., 2019 | Spain | 37 years | TS (GLM + DLNM) | 4,576,600 | Heat; mean temperature > MMT | CVD (I00-I99) | Mortality | 0-21 days | Total: 1.74 (1.63-1.84)  Men: 1.13 (0.99-1.25) Women: 2.25 (2.12-2.37)   ≥60 years total: 1.84 (1.72-1.94)   ≥60 years men: 1.22 (1.06-1.36) ≥60 years women: 2.30 (2.15-2.43) 60-74 years total: 1.05 (0.88-1.19)  60-74 years men: 0.88 (0.69-1.03) 60-74 years women: 1.36 (1.14-1.55) 75-89 years total: 1.88 (1.73-2.01) 75-89 years men: 1.24 (1.04-1.40) 75-89 years women: 2.32 (2.13-2.47)  ≥ 90 years total: 2.61 (2.39-2.79) ≥ 90 years men: 1.76 (1.52-1.94) ≥ 90 years women: 3.03 (2.82-3.20) |  |
| 47 | Denpetkul and Phosri, 2021 | 65 provinces, Thailand | 8 years | TS (GLM + DLNM) | All-cause: 2,891,407  CVD: 403,450  Respiratory diseases: 264,672 | Heat; mean temperature > MMT | CVD (I00-I99) and respiratory diseases (J00-J99) | Mortality | 0-7 days 0-14 days 0-21 days | All-cause: 2.45 (1.39- 4.46)  CVD: 0.66 (−0.17- 1.47)  Respiratory diseases: 2.51 (0.15- 4.75) | 0-7 days: All-cause: 70,839  CVD: 2,663  Respiratory diseases: 6,643 |
| 72 | He et. al., 2022 | Shandong Province, China | 7 years | TS (GLM + DLNM) | Total:138,749 Ischemic stroke: 86,873 Hemorrhagic stroke: 51,876 | Heat; mean temperature > MMT | Stroke (I60-I69) | Mortality | 0-14 days | Total: 4.02 (3.08-4.79) Ischemic stroke: 3.01 (1.64-4.00) Hemorrhagic stroke: 3.86 (0.21-6.38) |  |
| 139 | Ma et al., 2020 | Jiangsu province, China | 4 years | TS (GLM + DLNM) | 4,903,520 | Heat; mean temperature > MMT | Endocrine diseases (E00-E99), mental disorders (F00-F99), nervous disorders (G00-G99), CVD (I00-I99), stroke (I60-I69), respiratory diseases (J00-J99) | Mortality | 0-21 days | All-cause: 3.49 (2.87- 4.00)  Endocrine diseases: 3.76 (2.30- 4.91)  DM: 4.61 (0.13- 7.13)  Mental disorders: 9.95 (4.25- 12.54)  Nervous disorders: 8.49 (3.56- 11.97)  Alzheimer's and dementia: 10.68 (3.75- 14.28)  CVD: 4.96 (4.19- 5.60)  IHD: 3.76 (3.01- 4.48)  MI: 3.31 (2.13- 4.29)  Stroke: 6.67 (4.34- 8.50)  Hypertensive diseases: 6.23 (4.11- 7.78)  HHD: 7.20 (1.83- 10.47)  Respiratory diseases: 4.93 (3.27-6.21)  COPD: 4.60 (2.84- 5.89) |  |
| 217 | Wen et. al., 2021 | Brazil | 16 years | CC (logistic regression + DLNM) | 2,726,886 | Heat; mean temperature > MMT | Genitourinary (N00-N99) | Morbidity (HA) | 0-7 days | Total heat-related renal diseases: 7.4 (5.2- 9.6) Glomerular diseases 6.4 (4.2- 8.6)  Renal tubulointerstitial diseases 7.7 (5.4- 9.9) Kidney failure 7.3 (5.2- 9.3) | Total heat-related renal diseases: 202093 (141554- 260594) Glomerular diseases: 20157 (13031- 27041) Renal tubulointerstitial diseases: 110391 (77378- 142246) Kidney failure: 71544 (51145- 91307) |
| 233 | Yang et. al., 2015 | 15 cities, China | 7 years | TS (DLNM) | 1,936,116 | Heat; mean temperature > MMT | CVD (I00-I99) | Mortality | 0-21 days | 1.3 (1.0-1.5) | 25170 (19361- 29042) |
| 234 | Yang et. al., 2016 | 16 cities, China | 7 years | TS (DLNM) | 788,783 | Heat; mean temperature > MMT | Stroke (I60-I69) | Mortality | 0-21 days | 1.4 (1.1- 1.7) | 11043 (8677- 13409) |
| 260 | Zhou et. al., 2022 | Hubei and Jiangsu, China | 5 years | CC (logistic regression + DLNM | 15,888 | Heat; mean temperature > MMT - Moderate heat; MMT < mean temperature < 97.5^th^ percentile - Extreme heat; mean temperature > 97.5^th^ percentile | Asthma (J45) | Mortality | 0-21 days | 3.3 (0.5- 5.7) | 525 (83- 898) |
| 201 | Tong et al., 2021 | Perth, Australia | 8 years | TS (DLNM) | 79,899 | Heat; mean temperature > MMT | Disorders in general | Morbidity (EDV) | 0-28 days | 4.63 (1.91- 7.19) | 3697 (1529- 5744) |
| 17 | Bao et al., 2019 | Shenzhen, China | 12 years | TS (DLNM) | Total: 142569  Male: 86,709  Female: 55,860  <40: 11,624 40-64: 71,493 ≥65: 59,452  CBI: 104,339  ICH: 30,656 | Heat; mean temperature > MMT | Stroke (I60-I69) | Morbidity (HA) | 0-7 days | Total stroke: 1.95 (0.63-3.20)  Male: 2.04 (0.43-3.38)  Female: 1.86 (0.10-3.35)  <40: 1.65 (−0.68-3.83)  40-64: 2.04 (0.53-3.44)  ≥65: 1.87 (0.02-3.49)  CBI: 2.32 (0.75-3.59)  ICH: 0.46 (−1.77- 2.28) | Total stroke: 677 (181-1118)  Male: 431 (127-699)  Female: 252 (27-436)  <40: 48 (−28 -105)  40-64: 360 (102-622)  ≥65: 266 (5-476)  CBI: 610 (209-975)  ICH: 31 (−104- 153 |
| 53 | Fang et al., 2021 | Beijing, China | 5 years | TS (GAM + DLNM) | Total respiratory hospital visits: 877,793  URI: 383,459  Bronchitis: 189,180  Pneumonia: 173,036  Rhinitis: 60,670  Asthma: 22,169  Age:  < 2 years: 229,149  2-5 years: 466,328  6-14 years: 182,316  Male: 511,009  Female: 366,784 | Heat; mean temperature > MMT | Respiratory diseases (J00-J99) | Morbidity (HA) | 0-7 days | Total respiratory hospital visits: 0.9 (-0.5- 2.1)  URI: 1.2 (-0.7- 2.9)  Bronchitis: 0.9 (-0.3- 2.1)  Pneumonia: 1.2 (0.2- 2.0)  Asthma: 0.1 (-1.8- 2.0)  Age:  < 2 years: 1.2 (-0.4- 2.6)  2-5 years: 0.7 (-0.7- 2.0)  6-14 years: 0.9 (-0.9- 2.4)  Male: 1.0 (-0.5- 2.3)  Female: 0.8 (-0.5- 2.0) | Total respiratory hospital visits: 7,900  Upper respiratory infections (URI): 4,602  Bronchitis: 1,703  Pneumonia: 2,076  Asthma: 22  Age:  < 2 years: 2,750  2-5 years: 3,264  6-14 years: 1,641  Male: 5,110  Female: 2,934 |
| 160 | Onozuka et. al., 2017 | Japan | 10 years | TS (DLNM) | 659,752 | Heat; mean temperature > MMT - Moderate heat; MMT < mean temperature < 97.5^th^ percentile - Extreme heat; mean temperature > 97.5^th^ percentile | CVD (I00-I99) | Morbidity (OHCA) | 0-21 days | Total heat-related: 0.29 (0.21- 0.35)  Moderate heat: 0.10 (0.06- 0.14)  Extreme heat: 0.20 (0.15- 0.23) | Total heat- related: 1913 (1385- 2310) Moderate heat: 660 (396- 924) Extreme heat: 1320 (990- 1517) |
| 163 | Pan et. al., 2019 | Hefei, China | 10 years | TS (GLM + DLNM) | 36,607 | Heat; mean temperature > MMT | Mental health (F00-F99) | Morbidity (HA) | 0 days | Total: 7.41 (3.97-10.86) Male: 10.80 (5.94-15.68)  Female: 4.13 (-0.56-8.83) Married: 6.74 (2.67-10.82) Non-married: 8.02 (1.69-14.36)  Age 0-39: 7.38 (3.55-11.21)  Age ≥40: 7.81 (1.35-14.29) | Total: 1076.1 Male: 771 Female: 301 Married: 699.3  Non-married: 332 Age 0-39: 751.9  Age ≥40: 338.7 |
| 164 | Park et al., 2022 | South Korea | 11 years | TS (DLNM) | 213,610 | Heat; mean temperature > MMT | CVD (I00-I99) | Morbidity (prevalence) | 0-14 days | Total: 0.9 (0.1- 1.5)  Hypertension comorbidity: 1.2 (-0.4-2.0)  DM comorbidity: 2.7 (0.4-3.7)  Heart disease comorbidity: 2.7 (-1.5-5.2)  <65 years: 1.1 (-2.6-3.3)  ≥65 years: 1.5 (0.2-2.4)  Male: 0.8 (-0.1-1.3)  Female: 1.6 (0.1-2.6) | Total: 1,888.1 (276.8- 3,130.7) Hypertension comorbidity: 724.6 (-223.2-1203.1)  DM comorbidity: 1,071.3 (150.0- 1484.8)  Heart disease comorbidity: 746.1 (-419.5-1443.6)  <65 years: 837.1 (-2032.1- 2575.6)  ≥65 years: 1,990.1 (267.4- 3195.4)  Male: 1,039.3 (-128.7- 1789.2)  Female: 1,306.8 (93.4-2045.4) |
| 189 | Singh et. al., 2021 | India | 3 years | Cohort study (semi-parametric regression model) | 461 | Heat; mean temperature > MMT | Digestive system (K00-K95) and skin diseases (L00-L99) | Morbidity (prevalence) |  | Diarrhea: 7.46 (5.55- 9.32) Skin disease: 6.51 (2.84- 9.96) | Diarrhea: 34 (26-43) Skin disease: 30 (13-46) |
| 209 | Wang et. al., 2022 | China | 4 years | TS (GLM + DLNM) | 23,921 | Heat; mean temperature > MMT Moderate heat; MMT < mean temperature < 97.5^th^ percentile - Extreme heat; mean temperature > 97.5^th^ percentile | CVD (I00-I99) | Morbidity (HA) | 0-21 days | Heat: 3.8 (-0.4-8.1) Moderate heat: 3 (-0.6-6.3) Extreme heat: 0.8 (0.1-1.4) | Heat: 904 (-105-1914) Moderate heat: 715 (-146-1497) Extreme heat: 187 (25-325) |
| 227 | Xu et. al., 2022 | China | 5 years | CC (DLNM) | 1,000,014 | Heat; mean temperature > MMT | CVD (I00-I99) and stroke (I60-I69) | Mortality | 0-21 days | Total CVD: 3.70 (3.4- 3.97) Chronic rheumatic heart diseases: 6.81 (2.33- 10.9) Hypertensive diseases: 5.76 (4.79- 6.63) Hypertensive heart disease: 5.5 (4.63-6.44) Hypertensive renal disease: 4.33 (1.07- 7.28) Ischemic heart diseases: 3.9 (3.47 -4.37) Myocardial infarction: 3.93 (3.29- 4.49) Chronic ischemic heart disease: 3.95 (3.28- 4.67) Pulmonary heart disease: 1.38 (−2.22- 4.35) Stroke: 3.07 (2.67- 3.48) Hemorrhagic stroke: 1.97 (1.33- 2.60) Ischemic stroke: 3.20 (2.60- 3.70) Sequelae of stroke: 4.21 (3.47-4.89) Sequelae of hemorrhagic stroke: 3.53 (1.26- 5.56) Sequelae of ischemic stroke: 4.21 (3.29- 5.11) | Total CVD: 37001 (34400- 39701) |
| 6 | Adegboye et al., 2019 | North wet tropical region, Australia | 11 years | TS (DLNM) | 8,922 | Heat; mean temperature > MMT | Respiratory diseases (J00-J99) | Morbidity (HA) | 0-8 weeks | 7.68 (−5.12- 32.31) |  |
| 119 | Liu et al., 2020 | Hong Kong, China | 11 years | TS (DLNM) | Total: 400279  Male: 220702  Female: 179577  Age:  0-59: 55757  60-74: 87174  75-84: 126944  ≥85: 130404  Disease:  DM: 4335  Mental and behavioral disorders: 8150  Disease of the circulatory system: 83050  Disease of heart: 45185  Cerebrovascular disease: 32887  Disease of the respiratory system: 90651  Pneumonia: 67986  Chronic lower respiratory diseases: 15791  Renal disease: 21570 | Heat; mean temperature > MMT  Extreme heat; mean temperature >99^th^ percentile | Endocrine diseases (E00-E99), mental health disorders (F00-F99), CVD (I00-I99), respiratory diseases (J00-J99), genitourinary diseases (N00-N99) | Mortality | 0-21 days | Heat:  Total: 0.05 (-0.11-0.24)  Male: 0.09 (-0.16-0.34)  Female: 0.32 (-2.65-3.18)  Age:  0-59: 0.00 (-0.63-0.27)  60-74: 0.77 (-0.43-1.89)  75-84: 0.21 (-0.10-0.52)  ≥85: 1.89 (-2.77-6.04)  Disease:  DM: 10.67 (-14.03-26.24)  Mental and behavioral disorders: 4.49 (0.54-7.84)  Disease of the circulatory system: 0.07 (-0.13-0.28)  Disease of heart: 0.03 (-0.24-0.28)  Cerebrovascular disease: 0.55 (-1.33-2.36)  Disease of the respiratory system: 0.06 (-0.14-0.26)  Pneumonia: 0.03 (-0.20-0.24)  Chronic lower respiratory diseases: 0.00 (-1.05-0.55)  Renal disease: 1.28(-1.05-3.42)  Extreme heat:  Total: 0.11 (-0.06-0.30)  Male: 0.16 (-0.10-0.40)  Female: 0.04 (-0.26-0.31)  Age:  0-59: 0.00 (-0.47-0.43)  60-74: 0.16 (-0.19-0.48)  75-84: 0.27 (-0.05-0.56)  ≥85: 0.03 (-0.33-0.38)  Disease:  DM: 0.47 (-1.40-1.86)  Mental and behavioral disorders: 0.22 (-0.98-1.22)  Disease of the circulatory system: 0.15 (-0.25-0.51)  Disease of heart: 0.08 (-0.44-0.55)  Cerebrovascular disease: 0.05 (-0.53-0.57)  Disease of the respiratory system: 0.15 (-0.26-0.53)  Pneumonia: 0.08 (-0.37-0.50)  Chronic lower respiratory diseases: 0.34 (-0.63-1.01)  Renal disease: 0.00 (-0.93-0.48) | Heat:  Total: 192.92  Male: 202.92  Female: 566.81  Age:  0-59: 0.00  60-74: 675.14  75-84: 267.85  ≥85: 2465.20  Disease:  DM: 462.42  Mental and behavioral disorders: 366.24  Disease of the circulatory system: 60.55  Disease of heart: 11.67  Cerebrovascular disease: 181.81  Disease of the respiratory system: 53.67  Pneumonia: 17.77  Chronic lower respiratory diseases: 0.00  Renal disease: 276.02  Extreme heat:  Total: 425.51  Male: 358.01  Female: 72.56  Age:  0-59: 1.23  60-74: 135.86  75-84: 346.41  ≥85: 37.94  Disease:  DM: 20.29  Mental and behavioral disorders: 17.68  Disease of the circulatory system: 121.24  Disease of heart: 34.29  Cerebrovascular disease: 17.53  Disease of the respiratory system: 138.37  Pneumonia: 57.51  Chronic lower respiratory diseases: 37.69  Renal disease: 0.00 |
| 37 | Crank et al., 2022 | Arizona, USA | 9 years | TS (DLNM) | 86,672 | Warm: between 50^th^ and 90^th^ percentile  Hot: between 90^th^ and 100^th^ percentile | Mental health (F00-F99) | Morbidity (HA) | 0-7 days | Warm: 1.50 (−3.78-5.61)  Hot: 0.28 (−1.18-1.78) |  |
| 46 | Deng et al., 2019 | Southwest area, China | 8 years | TS (DLNM) | Total: 89,467  Cause-specific:  CVD: 41,794  Heart: 17,793  Stroke: 22,589  Respiratory: 16,565  Age:  ≤64 years: 21,678  65-74: 20,072  ≥75 years: 47,717  Gender:  Male: 48,939  Female: 40,528  Marital status:  Married: 54,971  Non-married: 34,496 | Heat; ambient temperature > median temperature Extreme heat; ambient temperature > 97^th^ percentile | CVD (I00-I99), respiratory diseases (J00-J99), stroke (I60-I69) | Mortality | 0-28 days | Total: 0.67 (−2.44-3.64)  Cause-specific:  CVD: −0.21 (−5.04-4.33)  Heart: −1.23 (−8.59- 5.46)  Stroke: 0.49 (−6.18-6.15)  Respiratory: 1.93 (−5.08-7.90)  Age:  ≤64 years: 0.08 (−6.64-5.64)  65-74: −1.25 (−7.4-5.01)  ≥75 years: 1.53 (−2.96-5.09)  Gender:  Male: 0.49 (−3.82-4.31)  Female: 0.89 (−3.21-5.03)  Marital status:  Married: 1.50 (−2.67-4.83)  Non-married: −0.61 (−5.55-3.95) | Total: 599  Cause-specific:  Stroke: 111  Respiratory: 320  Age:  ≤64 years: 17  ≥75 years: 730  Gender:  Male: 237  Female: 361  Marital status:  Married: 825 |
| 229 | Xu et. al., 2019 | Brazil | 16 years | CC (GLM + linear lag model) | 238.320 | Hottest 4 months | Undernutrition (E00-E99) | Morbidity (HA) | 0-7 days | 15.6 (9.0- 21.4) | 37129 (21511- 51032) |
| 228 | Xu et. al., 2019 | Brazil | 16 years | CC (lag linear model) | 553351 | Hottest 4 months | Endocrine diseases (E00-E99) | Morbidity (HA) | 0-3 days | Total DM hospitalizations: 7.3% (3.5- 10.9) DM 1: 6.6% (2.0- 19.9) DM2: 11.5% (6.1- 38.5) | Total DM hospitalizations: 40,543 (19533- 60389)  DM1: 11380 (3399- 34168)  DM2: 4368 (2328- 14607) |
| 77 | Hu et. al., 2020 | Shenzhen, China | 5 years | TS (GLM + DLNM) | Total: 681,318 Accidents: 306,569 Suicide: 6642 Assault: 46,756 Internal causes: 297,004 | Heat: 1°C increment from 17°C-33°C | Disorders in general | Morbidity (Emergency ambulance calls) | 0-7 days | Total: 10.72 (8.01-13.45) accidents: 12.13 (8.20-15.74) suicide:13.76 (-6.10-28.13) assault: 21.91 (13.59-28.61) internal causes: 7.87 (4.71-10.54) |  |
| 111 | Li et. al., 2021 | 10 cities, China | 4 years | TS (GLM) | 1,499,721 | Heat; 1°C increase in daily Tmax | Disorders in general | Morbidity (Emergency ambulance calls) | 0-7 days | Total: 11.7 (11.2-12.3) Central region, subtropical climate and lower PCDI 17.8 (17.2- 18.4) Central region, subtropical climate and higher PCDI 6.98 (6.56- 7.40) Northern region with temperate climate 7.32 (6.87- 7.78) Southern region with subtropical climate 7.11 (6.54- 7.66) |  |
| 255 | Zhao et. al., 2019 | Brazil | 16 years | CC (GLM + DLNM) | 49,145,997 | Hottest 4 months | Disorders in general | Morbidity (HA) | 0-7 days | 6.2 (3.3- 9.1) | 3070360 (1600393- 4468577) |
| 126 | Lu et. al., 2021 | Queensland, Australia | 22 years | TS (GLM + DLNM) | Total: 238,427 Climate hot 47,693 Climate mild 104,041 Climate cold 86,693 IRSAD low 83,370 IRSAD middle 84,616 IRSAD high 70,419 Male 123,788 Female 114,639 Age 0-59 120,733 60-74: 61,773 ≥75: 55,921 Kidney disease 63,147 Renal failure 22,695 Urolithiasis 60,119 Urinary tract infection 61,038 Other 54,123 | Heat; 1°C increase in mean daily temperature | Genitourinary disorders (N00-N99) | Morbidity (HA) | 0-10 days | Total: 19.2 (17.2- 21.2) climate hot: 15.8 (11.9- 19.6) climate mild: 21.7 (18.3- 25.0) climate cold: 18.4 (14.9- 21.7) IRSAD low: 18.0 (14.4- 21.5) IRSAD middle: 22.4 (19.2- 25.5) IRSAD high: 17.1 (13.3- 20.7) men 22.2 (20.0- 24.3) women 15.8 (13.3- 18.2)  age 0-59: 15.2 (12.8- 17.6)  age 60-74: 22.3 (19.8- 24.7)  age >/ 75: 24.2 (21.7 26.6) kidney disease: 19.6 (16.8- 22.2) renal failure: 31.2 (28.5- 33.7) urolithiasis: 25.8 (23.3- 28.2) urinary-tract infection: 16.2 (13.3- 19.1) other renal diseases: 13.8 (11.0- 16.5) | Total: 45 700 (40 900- 50 400)  climate hot: 7540 (5650-9330) climate mild: 22 600 (19 000- 26 000) climate cold: 15 900 (12 900- 18 700) IRSAD low: 15 000 (12 000- 17 900) IRSAD middle: 19 000 (16 200- 21 600) IRSAD high: 12 000 (9360- 14 500)  men: 27 400 (24 700- 30 100) women: 18 100 (15 200- 20 800)  0–59: 18 400 (15 400- 21 200)  60–74: 13 700 (12 200- 15 200) >/75: 13 500 (12 100- 14 800)  kidney disease: 12 300 (10 600- 14 000) Renal failure 7060 (6450- 7640) Urolithiasis 15 500 (14 000- 16 900) Urinary-tract infection 9880 (8090- 11 600) Other renal diseases 7470 (5950- 8940) |
| 193 | Su et. al., 2020 | Guangzhou, China | 2 years | TS (GLM + DLNM) | Total: 9,851 before policy implementation: 4,450  after policy implementation: 5401 | Extreme heat; Tmax > 33°C | Work related issues | Morbidity (prevalence) | 0-7 days | Before implementation extreme heat related: 3.17 (1.27-4.77) after implementation: 1.52 (-0.36-3.27) |  |
| 246 | Zhang et. al., 2021 | Ningbo, China | 4 years | TS (GLM + DLNM) | Total: 129,897 male: 76,846 female: 53,051  age 0-3: 99,284  age 4-5: 22,331  age ≥6: 8282 | Heat; 16°C < mean temperature < 29°C | Infectious diseases (A00-B99) | Morbidity (Incidence) | 0-30 days | Total: 39.55 (30.91-45.51) male 36.61 (27.02-44.29) female 43.15 (34.21-48.94) age 0-3: 38.31 (29.88-44.79) age 4-5: 44.16 (34.93-51.28)  age ≥6: 43.74 (26.05-56.20) |  |
| 2 | Aboubakri et al., 2019 | Southeastern region, Iran | 18 years | TS (DLNM) | 46,200 | Heat; mean temperature > MMT  Mild heat: MMT < mean temperature < 99th  Extreme heat: mean temperature > 99^th^ percentile | Disorders in general | Mortality | 0-14 days | Heat:  Total: 2.23(0.34-3.87)  Male: 1.72(-0.83-4.05)  Female: 2.94(0.11-5.44)  <65 years: 0.7(-1.82-3.43)  ≥65 years: 3.73(1.26-5.82)  Mild heat:  Total: 2.02(0.3-3.69)  Male: 1.49(-0.71-3.59)  Female: 2.74(0.04-5.06)  <65 years: 0.63(-2.05-2.98)  ≥65 years: 3.38(1.04-5.34)  Extreme heat:  Total: 0.25(0.06-0.41)  Male: 0.22(-0.04-0.45)  Female: 0.27(-0.04-0.51)  <65 years: 0.06(-0.23-0.29)  ≥65 years: 0.44(0.15-0.69) |  |
| 27 | Chen et al., 2018 | China | 3 years | TS (GLM + DLNM) | 1,826,186 | Heat; mean temperature > MMT  Moderate heat: MMT < mean temperature < 97.5th  Extreme heat: mean temperature > 97.5^th^ percentile | CVD (I00-I99) and respiratory diseases (J00-J99) | Mortality | 0-21 days | Total: 2.71 (1.98- 2.98)  Temperate monsoon: 5.28 (4.11-6.41)  Subtropical monsoon: 1.11 (0.74-1.46)  Temperate continental: 6.57 (−55.68-48.62)  Alpine: 2.24 (−50.21-28.37)  Tropical monsoon: 0.37 (−7.05-10.42) |  |
| 61 | Fu et. al., 2018 | India | 13 years | CC (DLNM) | Total: 591,121 Stroke (30-69 years): 21,209 Ischemic heart diseases (30-69 years): 41,989 Respiratory diseases (30-69 years): 25,090 | Heat; mean temperature > MMT  Moderate heat: MMT < mean temperature < 97.5th  Extreme heat: mean temperature > 97.5^th^ percentile | CVD (I00-I99), respiratory diseases (J00-J99) and stroke (I60-I69) | Mortality | 0-21 days | Moderate heat:  total 0.2 (-0.1-0.5)  Age 0-29: 3.1 (−2.7-7.8)  age 30-69: 0.3 (-0.1-0.8) age≥70: 0.2 (-0.2-0.6) Stroke  age 30-69: 0.1 (-0.0-0.1) Ischemic heart disease age 30-69: 1 (-0.1-2) Respiratory diseases age 30-69: 1.1 (-2-3.9)  Extreme heat: total: 0.3 (0.1-0.4) Age 0-29: 0.4 (-0.1-0.7)  age 30-69: 0.3 (0.1-0.5)  age ≥70: 0.4 (0.2-0.6) Stroke  age 30-69: 0.4 (0.0-0.7) ischemic heart disease age 30-69: 0.2 (-0.2-0.5) respiratory diseases age 30-69: 0.4 (-0.1-0.8) |  |
| 161 | Ordanovich et. al., 2023 | Spain | 40 years | TS (GLM + DLNM) | 1979-1988: 3,014,169 1989-1998: 3,412,755 1999-2008: 3,747,522 2009-2018: 4,029,513 | Moderate heat; mean temperature < MMT < 95th  Extreme heat; mean temperature >95th | Disorders in general | Mortality | 0-21 days | Total: 1.38 (1.38-1.39) 1979-1988 moderate heat: 0.34 (0.23-0.46) extreme heat: 0.49 (0.41-0.56) 1989-1998 moderate heat: 0.89 (0.52-1.26) extreme heat: 0.75 (0.62-0.87) 1999-2008 moderate heat: 1.10 (0.39-1.77) extreme heat: 0.59 (0.47-0.71) 2009-2018 moderate heat: 0.64 (0.19-1.09) extreme heat: 0.49 (0.39-0.59) |  |
| 167 | Pascal et. al., 2023 | France | 46 years | TS (GLM + DLNM) | 5,499,200 | Heat; >90th percentile of mean temperature | Disorders in general | Mortality | 0-21 days | 0.19 (0.19-0.20) | 10,200 (9,544-10,741) |
| 207 | Vicedo-Cabrera et. al., 2021 | 43 countries | 28 years | TS (GLM + DLNM) | 29,936,896 | Heat; 99^th^ percentile of mean temperature during warm season | Disorders in general | Mortality | 0-10 days | 37 |  |
| 250 | Zhang et. al., 2019 | China | 7 years | TS (GLM + DLNM) | Total: 383674 CVD: 163,509 Respiratory diseases: 37,517 | Heat; mean temperature > MMT  Moderate heat: MMT < mean temperature < 97.5th  Extreme heat: mean temperature > 97.5^th^ percentile | CVD (I00-I99) and respiratory diseases (J00-J99) | Mortality | 0-21 days | Heat (all): total deaths: 1.73 (1.19- 2.27) CVD: 3.35 (2.64- 4.06) respiratory: 3.3 (2.20- 4.54) Moderate heat: total deaths: 1.02 (0.63- 1.42) CVD: 2.01 (1.52-2.49) respiratory: 2.13 (1.20- 3.06) Extreme heat: total deaths: 0.71 (0.54- 0.89) CVD: 1.35 (1.18-1.52) respiratory: 1.24 (1.01- 1.47) | Heat: Total mortality: 6638 (4566- 8709) Moderate heat: Total mortality: 3913 (2417- 5448) Extreme heat: Total mortality: 2724 (2072- 3184) |
| 261 | Scovronick et. al., 2018 | South Africa | 17 years | TS (GLM+DLNM) | 7,576,674 | Heat; mean temperature > MMT  Moderate heat: MMT < mean temperature < 97.5th  Extreme heat: mean temperature > 97.5^th^ percentile | Disorders in general | Mortality | 0-2 days | Total: 0.4 Total extreme heat related mortality: 0.13 (0.11- 0.17) |  |
| 59 | Ferreira et. al., 2019 | 6 cities, Brazil | 18 years | TS (GLM + DLNM) | 118,898 | Mild heat; MMT < mean temperature < 90^th^ percentile Moderate heat; 90^th^ percentile < mean temperature < 97.5^th^ percentile Extreme heat; 97^th^ percentile < mean temperature < highest temperature | CVD (I00-I99) | Mortality | 0-14 days | Mild heat: 0.2 (0.1-0.2) Moderate heat: 0.8 (0.6-1) Extreme heat: 0.8 (0.6-1) |  |
| 150 | Mascarenhas et. al., 2022 | Brazil | 20 years | TS (GLM + DLNM) | Belem: 21,629 Manaus: 13,036 Fortaleza: 30,733 Salvador: 32,799 Brasília: 18,894 Campo Grande 9,210  Rio de Janeiro: 168,480 São Paulo 152,919 Curitiba: 31,481 Porto Alegre: 52,552 | Mild heat; MMT < mean temperature < 90^th^ percentile Moderate heat; 90^th^ percentile < mean temperature < 97.5^th^ percentile Extreme heat; 97^th^ percentile < mean temperature < highest temperature | CVD (I00-I99) | Mortality | 0-14 days | 3.21 (3.13-3.29)  Mild heat Manaus: 3.1 (0.9-4.9) Brasília: 2.1 (0.2-3.9)  Rio de Janeiro 0.6 (0.1-1.1) Moderate heat Manaus: 2.9 (1.6-4) Brasília: 1.8 (0.8-2.8)  Rio de Janeiro: 1.1 (0.8-1.4)  São Paulo: 0.6 (0.4-0.8)  Porto Alegre: 1.3 (0.88-1.9) Extreme heat Manaus: 1.3 (0.8-1.8) Brasília: 0.7 (0.3-1) Rio de Janeiro 0.8 (0.6-0.9) São Paulo: 0.5 (0.4-0.7)  Porto Alegre: 1 (0.8-1.2) |  |
| 192 | Su et. al., 2021 | 5 cities, China | 4 years | TS (GLM + DLNM) | 12,132 | Heat; 97.5th percentile of weekly maximum temperature | Nervous system (G00-G99) | Mortality | 0-5 weeks | 3.65 (0.42-6.17) |  |
| 84 | Iñiguez et. al., 2020 | 52 cities, Spain | 25 years | TS (GLM + DLNM) | Mortality: CVD 732,851 respiratory diseases 262,493 HA:  CVD 504,230 respiratory diseases 472,544 | Heat; 97.5th percentile of mean temperature | CVD (I00-I99) and respiratory diseases (J00-J99) | Mortality and morbidity (HA) | 0-21 days | CVD mortality: 1.2 (0.1-2.2)  CVD HA: 0 (0-0) respiratory diseases mortality: 9.4 (3.1-15) respiratory diseases HA: 0.2 (-0.3-0.6) |  |
| 19 | Bernstein et al., 2022 | 47 childrens’ hospitals, USA | 3 years | TS (GLM + DLNM) | Total: 67,942 Male: 31,598, Female: 36,344 <15: 11,999  15-59: 31,004 ≥60: 24,939 | Heat; maximal temperature > MMT | Infectious diseases (A00-B99), digestive system (K00-K95), nervous system (G00-G99), skin diseases (L00-L99), endocrine diseases (E00-E99), CVD (I00-I99), respiratory diseases (J00-J99), mental health disorders (F00-F99), musculoskeletal system (M00-M99) and genitourinary diseases (N00-N99) | Morbidity (EDV) | 0-21 days | Moderate heat:  heat related illness: 28.3 (15.6- 33.8)  otitis media and externa: 12.0 (7.2- 15.3)  bacterial enteritis: 23.7 (12.1- 29.2)  infectious and parasitic diseases: 12.7 (10.6- 14.4)  blood and immune system disorders: 15.8 (10.0- 19.9)  nervous system diseases: 13.6 (5.9- 18.0)  skin and soft tissue infections: 16.6 (14.1- 18.6)  other skin and soft tissue diseases: 15.7 (12.5- 18.1)  other sign and symptoms: 10.5 (7.5- 12.8)  Endocrine nutritional and metabolic disease: 18.0 (7.7- 22.7)  CVD: 9.6 (-4.1- 15.8)  Digestive system diseases: 11.0 (7.2- 13.9)  All-cause: 11.0 (9.4- 12.6)  Mental, behavioral and neurodevelopmental disorders: 9.3 (5.6- 11.9)  Injury and poisoning: 17.0 (14.0- 19.3)  Genitourinary system diseases: 9.1 (5.9- 11.5)  External causes and other health factors: 11.2 (2.7- 15.8)  Respiratory system diseases: 8.1 (4.5- 10.7)  Musculoskeletal system diseases: 10.2 (4.3- 14.0)  Asthma: 10.9 (5.5- 14.6)  perinatal conditions: 6.3 (-1.6- 10.9)  suicidality and depression: 3.5 (-1.4- 6.6)  Extreme heat:  heat related illness: 2.6 (1.9- 2.9)  otitis media and externa: 1.5 (1.1- 1.7)  bacterial enteritis: 1.4 (0.8- 1.7)  infectious and parasitic diseases: 1.2 (1.0- 1.4)  blood and immune system disorders: 1.2 (0.9- 1.5)  nervous system diseases: 0.9 (0.5- 1.2)  skin and soft tissue infections: 1.2 (0.9- 1.3)  other skin and soft tissue diseases: 1.1 (0.7- 1.3)  other sign and symptoms: 0.9 (0.7- 1.0)  Endocrine nutritional and metabolic disease: 1.3 (0.5- 1.6)  CVD: 1.1 (0.6- 1.5)  Digestive system diseases: 0.8 (0.5- 0.9)  All-cause: 0.7 (0.6- 0.8)  Mental, behavioral and neurodevelopmental disorders: 0.7 (0.5- 0.8)  Injury and poisoning: 0.8 (0.6- 0.9)  Genitourinary system diseases: 0.5 (0.2- 0.7)  External causes and other health factors: 0.8 (0.4- 1.1)  Respiratory system diseases: 0.6 (0.4- 0.7)  Musculoskeletal system diseases: 0.4 (0.0- 0.6)  Asthma: 0.8 (0.5- 1.0)  perinatal conditions: 0.4 (-0.0- 0.7)  suicidality and depression: 0.3 (0.0- 0.5) | Moderate heat:  heat related illness: 8135 (4478- 9719)  otitis media and externa: 16241 (9750-  20670)  bacterial enteritis: 9843 (5010- 12119)  infectious and parasitic diseases: 34092 (28644- 38623)  blood and immune system disorders: 5210 (3282- 6562)  nervous system diseases: 9176 (4011- 12180)  skin and soft tissue infections: 16002 (13609- 17940)  other skin and soft tissue diseases: 14467 (11463- 16633)  other sign and symptoms: 78415 (56547- 96051)  Endocrine nutritional and metabolic disease: 4604 (1982- 5801)  CVD: 1758 (-757- 2896)  Digestive system diseases: 28722 (18812- 36387)  All-cause: 420745 (357890- 480468)  Mental, behavioral and neurodevelopmental disorders: 6477 (3941- 8349)  Injury and poisoning: 157737 (13034- 179733)  Genitourinary system diseases: 9806 (6345- 12459)  External causes and other health factors: 9384 (2274- 13216)  Respiratory system diseases: 47596 (26176-62729)  Musculoskeletal system diseases: 10503 (4391-14380)  Asthma: 12848 (6497- 17211)  perinatal conditions: 2922 (-730-5084)  suicidality and depression: 1155 (-460- 2202)  Extreme heat:  heat related illness: 760 (547- 837)  otitis media and externa: 1964 (1536- 2237)  bacterial enteritis: 599 (338- 720)  infectious and parasitic diseases: 3208 (2563- 3694)  blood and immune system disorders: 404 (289- 486)  nervous system diseases: 624 (310- 800)  skin and soft tissue infections: 1117 (884- 1278)  other skin and soft tissue diseases: 971 (616- 1189)  other sign and symptoms: 6379 (5042- 7360)  Endocrine nutritional and metabolic disease: 322 (126- 406)  CVD: 209 (101- 266)  Digestive system diseases: 2016 (1377- 2474)  All-cause: 27351 (23866- 30357)  Mental, behavioral and neurodevelopmental disorders: 460 (336- 563)  Injury and poisoning: 7206 (5485- 8479)  Genitourinary system diseases: 546 (186- 775)  External causes and other health factors: 710 (302- 936)  Respiratory system diseases: 3380 (2084- 4281)  Musculoskeletal system diseases: 441 (25- 655)  Asthma: 969 (571- 1218)  perinatal conditions: 199 (-6.4- 340)  suicidality and depression: 108 (12- 177) |
| 5 | Adegboye et al., 2019 | Afghanistan | 7 years | TS (GLM + DLNM) | Total: 67,942 Male: 31,598 Female: 36,344 <15: 11,999  15-59: 31,004 ≥60: 24,939 | Moderate heat; MMT < monthly mean temperature < 97.5th percentile Extreme heat; monthly mean temperature >97.5th percentile | Infectious disease: leishmania (A00-B99) | Morbidity (prevalence) | 0-12 months | Moderate heat:  Total: 1.5 (1.3-1.6)  Male: 1.0 (0.7-1.2)  Female: 0.8 (0.5-1.0)  <15: 1.7 (1.5-1.9)  15-59: −0.4 (−0.9-0.1)  ≥60: 2.1 (2.0-2.2)  Extreme heat:  Total: 1.7 (1.6-1.8)  Male: 1.2 (0.9-1.4)  Female: 1.0 (0.5-1.2)  <15: 1.9 (1.7-2.1)  15-59: −0.3 (−1.1-0.3)  ≥60: 2.5 (2.5-2.9) | Moderate heat:  Total: 1,019  Male: 316  Female: 291  <15: 204  ≥60: 524  Extreme heat:  Total: 1,155  Male: 379  Female: 363  <15: 228  ≥60: 623 |
| 31 | Chen et al., 2022 | Beijing, China | 4 years | CC (DLNM) | 18,500 | Moderate heat; MAT (22 °C) < mean temperature < 97.5^th^ percentile of temperature  Extreme heat; > 97.5^th^ percentile of temperature | Respiratory diseases (J00-J99) | Morbidity (HA) | 0-30 days | Moderate heat:  Total: 5.5 (3.0- 7.8)  Male: 3.1 (− 1.0- 6.6)  Female: 7.0 (4.3- 9.5)  19-64 years: 6.9 (4.0- 9.5)  ≥65 years: 3.3 (− 0.5- 6.5)  Extreme heat:  Total: 2.2 (1.3- 3.0)  Male: 1.3 (0- 2.4)  Female: 2.8 (1.8- 3.7)  19-64 years: 2.8 (1.8- 3.7)  ≥65 years: 1.3 (0.1- 2.4) | Moderate heat:  Total: 1017 (550- 1424)  Male: 233 (− 74- 496)  Female: 768 (477- 1035)  19-64 years: 763 (442- 1048)  ≥65 years: 239 (− 31- 473)  Extreme heat:  Total: 404 (247- 547)  Male: 95 (− 2- 181)  Female: 307 (202- 406)  19-64 years: 308 (196- 408)  ≥65 years: 94 (3- 174) |
| 147 | Martínez-Solanas et. al., 2018 | Spain | 20 years | TS (GLM + DLNM) | 15,992,310 | Moderate heat; MMT < mean temperature < 97.5^th^ percentile of temperature  Extreme heat; > 97.5^th^ percentile of temperature | Work related issues | Morbidity (incidence) | 0-4 days | Heat total: 2.40 (2.09-2.68) moderate heat: 2.24 (1.95-2.52) extreme heat 0.17 (0.16-0.18) |  |
| 171 | Qiu et. al., 2016 | Hong Kong, China | 8 years | TS (GLM + DLNM) | 9,7316 | Moderate heat; MMT < mean temperature < 99^th^ percentile of temperature  Extreme heat; > 99^th^ percentile of temperature | Respiratory diseases (J00-J99) | Morbidity (HA) | 0-21 days | Total: 2.02 (-0.15- 3.78) Moderate heat: 1.93 (0.23- 3.71) Extreme heat: 0.09 (0.02- 0.15) | Total: 3981 (385-7527) Moderate heat: 3801 (140,7541) Extreme heat: 168 (34-294) |
| 256 | Zhao et. al., 2019 | Dongguan, China | 5 years | TS (GLM + DLNM) | Total: 75,015 COPD: 11,068 Asthma: 28,059 Bronchiectasis: 7,991 | Moderate heat; MMT < mean temperature < 95^th^ percentile of temperature  Extreme heat; > 95^th^ percentile of temperature | Respiratory diseases (J00-J99) | Morbidity (OV) | 0-7 days | Total moderate heat: 7.5 (2.4-12.2) Total extreme heat: 0.6 (0.0-1.1) COPD moderate heat: 2.9 (-4.4-9) COPD extreme heat: 0.6 (-0.7-1.5) Bronchiectasis moderate heat: 8.8 (-3.3-18.8) bronchiectasis extreme heat: 1.2 (-0.3-2.4) asthma moderate heat: 7.8 (0.2 - 14.5) asthma extreme heat: 0.3 (-0.6-1.2) | Total moderate heat: 5599 (1896-9239) Total extreme heat: 446 (-11-868) COPD moderate heat: 317(-487-1008) COPD extreme heat: 63 (-71-172) Bronchiectasis moderate heat: 702 (-244-1463) bronchiectasis extreme heat: 97 (-28-195) asthma moderate heat: 2180 (26-4088) asthma extreme heat: 96 (-176-318) |
|  | Kim et. al., 2015 | Seoul, South Korea | 18 years | TS (GLM) | Total: 676,509 Accidental and external causes of morbidity and mortality: 80,033 transport accident: 22,220  all CVD: 169,882 ischemic heart disease: 32,257 hypertensive disease: 11,413 heart failure: 6,758 myocardial infarction: 27,288 stroke, cerebrovascular: 96,947 chronic ischemic heart disease: 4,871 sudden death: 8,678  all respiratory: 34,904 asthma: 6,748 COPD: 10,566 Pneumonia: 10,114 Endocrine, nutritional and metabolic: 30,685 DM: 28,123  All MH: 12,468 Organic MH: 8,015 Substance misuse: 3,504 Schizophrenia: 592 Self-harm: 25,476 Digestive system: 36,382 Nervous system: 10,295 Genitourinary: 10,552 Diseases of the blood and blood-forming organs and certain disorders involving the immune mechanism: 1,555 | Heat; mean temperature > 90^th^ percentile of maximum temperature | Mental health disorders (F00-F99), nervous system (G00-G99), CVD (I00-I99), respiratory diseases (J00-J99) and genitourinary diseases (N00-N99) | Mortality |  | Total: 1.17 accidental and external causes of morbidity and mortality: 1.35 transport accidents: 0.54  all CVD: 1.48 ischemic heart disease: 0.83 hypertensive disease: 1.46  heart failure: 0.46 myocardial infarction: 0.87 stroke, cerebrovascular: 1.84 chronic ischemic heart disease: 0.56 sudden death: 2.05 all respiratory: 0.86 asthma: 2.10 COPD: 0.3 Pneumonia: 1.16 Endocrine, nutritional and metabolic: 1.18  DM: 1.10  All MH: 1.83 Organic MH: 1.40 Substance misuse: 3.27 Schizophrenia: 0 Self-harm: -1.27 Digestive system: 0.57 Nervous system: 3.06 Genitourinary: 2.21 Diseases of the blood and blood-forming organs and certain disorders involving the immune mechanism: 0.74 | Total: 3,177 accidental and external causes of morbidity and mortality: 480 transport accidents: 53 all CVD: 975 ischemic heart disease: 105 hypertensive disease: 64 heart failure: 13 myocardial infarction: 93 stroke, cerebrovascular: 685 chronic ischemic heart disease: 11 sudden death: 65 all respiratory: 116 asthma: 54  COPD: 12 Pneumonia: 45 Endocrine, nutritional and metabolic: 135  hospitDM: 114  All MH: 87 Organic MH: 42 Substance misuse: 46 Schizophrenia: 0  Self-harm: -141 Digestive system: 83 Nervous system: 121 Genitourinary: 94 Diseases of the blood and blood-forming organs and certain disorders involving the immune mechanism: 5 |
| 104 | Lee et. al., 2017 | Japan, South Korea, Taiwan, Vietnam and Philippines | 42 years | TS (GLM + DLNM) | Non-accidental: 36,089,344 CVD: 13,317,378Respiratory diseases: 4,608,350 | Extreme heat; 97.5^th^ percentile < mean temperature < 100^th^ percentile | CVD (I00-I99) and respiratory diseases (J00-J99) | Mortality | 0-28 days | Non-accidental deaths: extreme hot 0.16 (0.14-0.18) CVD: extreme heat 0.26 (0.24-0.29) Respiratory diseases: extreme heat 0.26 (0.23-0.28) |  |
| 263 | Pattenden et. al., 2003 | Sofia, Bulgaria and London, England | 4 years | TS (GAM + DLNM) | Sofia: 44,701 London: 256,464 | Heat; mean temperature > 90^th^ percentile | Disorders in general | Mortality | 0-25 days | Total Sofia: 0.72 (0.47-0.96) Total London: 0.44 (0.33-0.56) |  |
| 146 | Martínez-Solanas and Basagaña, 2019 | Spain | 11 years | TS (GLM + DLNM) | 7,378,435 | Heat; maximum temperature >MMT  Moderate heat: MMT < maximum temperature < 97.5^th^ percentile Extreme heat: >97.5th percentile of maximum temperature | CVD (I00-I99), respiratory disease (J00-J99), nervous system (G00-G99), Mental health disorders (F00-F99) and endocrine disorders (E00-E99) | Mortality | 0-21 days | Heat: 0.99 (0.69-1.24) moderate heat: 0.38 (0.12-0.62) extreme heat: 0.67 (0.61-0.72) |  |
| 221 | Wu et. al., 2021 | China | 8 years | TS (GLM + DLNM) | 42,022 | Heat; > optimal temperature | Disorders in general | Morbidity (EAD) | 0-21 days | 2011: 4.9 (0.9- 8.3) 2013: 4.2 (1.8- 6.7) | 2011: 2059 (378- 3488) 2013: 1765 (756- 2815) |
| 33 | Cheng et. al., 2017 | Hefei, China | 7 years | TS (GLM + DLNM) | 12,717 | Mild heat; 18.4-31.2°C Extreme heat; >31.2°C | Infectious diseases (A00-B99) | Morbidity (incidence) | 0-9 days | Total heat: 18.74 (8.36-27.44) Moderate heat: 17.12 (7.34-25.32) Extreme heat: 2.07 (0.75-3.25) Urban: 26.87 (16.21-36.68) Rural: -1.9 (-25.03-16.05) |  |
| 101 | Lee et. al., 2018 | 6 cities, South Korea | 11 years | TS (GLM + DLNM) | Total: 166,579 <65: 115.494 ≥65: 51.085 anxiety: 18.771 schizophrenia: 23.685 depression: 35.095 dementia: 6.067 | Heat; 99th of mean temperature | Mental health disorders (F00-F99) | Morbidity (EDV) | 0-7 days | Total: 14.6 (8.8- 20.4) Schizophrenia: 19.2 (4-34) anxiety: 31.6 (16- 47.2) dementia: 20.5 (1.5- 39.5) depression: 11.6 (−0.3- 23.5)  <65: 12.8 (4.8-20.8) ≥65: 19.1 (8.8- 29.4) | Total: 1663.378 (1002.584- 2324.172) Schizophrenia: 191.424 (39.88- 342.968) anxiety: 466.1 (236- 696.2) dementia: 287.82 (21.06- 554.58) depression: 263.668 (−6.819- 534.155) <65: 976.64 (366.24- 1587.04) 65≥: 828.835 (428.995- 1228.675) |
| 156 | Nguyen et. al., 2022 | Hanoi, Vietnam | 5 years | TS (GLM + DLNM) | City center: 11,427 outer city: 12.403 citywide: 23,830 | Mild heat; 75-98^th^ percentiles of mean temperature extreme heat; 99-100^th^ of mean temperature | Respiratory diseases (J00-J99) | Morbidity (HA) | 0-21 days | Mild heat: center 0.17 (-1.72-1.74) outer: -0.14 (-1.88-1.30) citywide: 0.05 (-0.35-0.40)  extreme heat: center 0.35 (-0.06-0.61) outer: 0.32 (-0.09-0.58) citywide: 0.33 (0.24-0.39)  total heat center 0.52 (-1.73-2.30) outer: 0.19 (-1.91-1.86) citywide: 0.38 (-0.14-0.77) | Mild heat: center 18 (-195-195) outer: -6 (-219-172) citywide: 12 (-85-96) extreme heat:  center 40 (-6-69)  outer: 39 (-9-69)  citywide: 79 (58-94) |
| 244 | Zhan et. al., 2022 | Fujian, China | 7 years | TS (GLM + DLNM) | Total: 2,024,147 Hypertension 509,216 Ischemic heart disease 318,354 Acute ischemic heart disease 56,270 Chronic ischemic heart disease: 262,066 Stroke 435,579 Ischemic stroke 341,315 | Heat; apparent temperature of 97.5^th^ percentile | CVD (I00-I99) and stroke (I60-I69) | Morbidity (HA) | 0-28 days | Total: 13.56 (-86-28.63) Hypertension: -8.35 (-43.46-17.41) Ischemic heart disease: 19.46 (1.26-33.48) Chronic ischemic heart disease: 22.62 (6.69-34.74) Cerebrovascular disease: 19.55 (-7.05-38.59) stroke: 7.47 (-23.15-29.09) ischemic stroke 19.18 (-11.72-40.28) |  |
| 25 | Cao et al., 2021 | 10 cities, China, Korea and Taiwan | China: 4 years  Korea: 11 years  Thailand: 4 years | TS (GAM + DLNM) | Total: 1416091  Thailand: Chiang Mai: 34,195  Thailand: Bangkok: 183,275  Korea: Seoul: 375,082  Korea: Busan: 183,275  Korea: Daegu: 111,032  Korea: Incheon: 106,282  Korea: Gwangju: 57,216  Korea: Daejeon: 56,098  China: Tianjin: 245,847  China: Ningbo: 128,295 | Heat; apparent temperature > MMT | Disorder in general | Mortality | 0-21 days | Thailand: Chiang Mai: 2.67 (-4.51- 9.09)  Thailand: Bangkok: 8.61 (-3.74- 19.45)  Korea: Seoul: 0.28 (-0.15- 0.68)  Korea: Busan: 0.39 (-0.70- 1.44)  Korea: Daegu: 0.28 (-0.33- 0.81)  Korea: Incheon: 0.29 (-0.62- 1.34)  Korea: Gwangju: 0.30 (-0.21- 0.78)  Korea: Daejeon: 0.46 (-0.21- 1.03)  China: Tianjin: 0.46 (0.13- 0.72)  China: Ningbo: 1.30 (-0.92- 3.35)  Total: 1.18 (0.14- 2.15) | Thailand: Chiang Mai: 913  Thailand: Bangkok: 15,780  Korea: Seoul: 1,050  Korea: Busan: 715  Korea: Daegu: 311  Korea: Incheon: 308  Korea: Gwangju: 171  Korea: Daejeon: 258  China: Tianjin: 1,131  China: Ningbo: 1,668  Total: 16,710 |
| 54 | Fang et. al., 2023 | 335 locations, China | 12 years | TS (GLM + DLNM) | Total: 1,522,963 male: 889,586 female: 633,329 age 0-64: 418,538 age 65-84: 798,609 age ≥85: 305,814 CVD: 329,228 cerebrovascular: 281,476 respiratory: 207,805 urban 728,981 rural: 793,982 | Hot events; high mean temperature Dry-hot events; high temperature and low humidity  Wet-hot events; high temperature and high humidity | CVD (I00-I99) and respiratory diseases (J00-J99) | Mortality | 0-3 days | Hot events total: 9.43 (8.94-10.13) males: 6.90 (6.38-7.68) females: 12.65 (12.04-13.55) age 0-64: 4.44 (3.96-5.20)  age 65-84: 9.24 (8.75-9.97)  age ≥85: 16.29 (15.59-17.83)  CVD: 12.95 (12.82-13.09) cerebrovascular: 11.37 (11.24-11.51) respiratory: 11.31 (11.15-11.50) urban: 10.51 (9.91-11.26) rural: 8.12 (7.36-9.20) |  |
| 122 | Liu et. al., 2020 | Shenzhen, China | 5 years | TS (GLM + DLNM) | 85,833 | Mild heat; 75-95^th^ percentile of apparent temperature Moderate heat; 95-99^th^ percentile of apparent temperature Extreme heat; >99th percentile of apparent temperature | Mental health disorders (F00-F99) | Morbidity (EDV) | 0-10 days | Mild heat Total: 4.21 (1.74-6.30) Male: 4.21(1.67-6.41) Female: 5.05 (−1.00-9.37)  15-29 years old: 7.03 (3.38-9.95)  30-44 years old: 4.16 (1.22-6.65)  45-59 years old: 1.2 (−1.86-3.85) | Mild heat Total: 181 (73,273)  male: 153 (62,232) female: 183 (−5,45)  15-29 years old  30-44 years old: 117 (59,166)  45-59 years old: 77 (23,123) |
| 214 | Wang et. al., 2021 | China | 3 years | TS (GLM + DLNM) | 183,726 | Extremely high DTR; >99^th^ percentile Extremely low DTR <1^st^ percentile | CVD (I00-I99) and respiratory diseases (J00-J99) | Mortality | 0-21 days | Total non-accidental DTR mortality: 15.52 (6.97 21.85) low DTR: 1.04 (0.09- 1.68)  high DTR: 14.48 (5.77- 20.72) extreme low DTR: 0.11 (0- 0.18) extreme high DTR: 0.32 (0.22- 0.40) Total CVD DTR mortality: 15.27 (5.96- 21.40) low DTR: 0.59 (−0.49- 1.37)  high DTR: 14.68 (5.87- 21.00) extreme low DTR: 0.06 (−0.08- 0.15) extreme high DTR: 0.37 (0.21- 0.46) Total respiratory DTR mortality: 9.14 (−2.31- 17.51) low DTR: 0.07 (−0.63- 0.55) high DTR: 9.07 (−3.06- 0.55) extreme low DTR: 0% (−0.12- 0.07) extreme high DTR: 0.42 (0.27- 0.51) | Total non-accidental DTR mortality: 28514 (12806- 40144)  CVD DTR mortality: 28055 (10950- 39317) Respiratory DTR mortality: 16793 (−4244-32170) |
| 222 | Wu et. al., 2022 | Global | 47 years | TS (GLM + DLNM) | 36,420,000 | TV | Disorders in general | Mortality | 0-10 days | Total TV mortality: Q1: 0.70 (-0.33- 1.69) Q2: 1.34 (-0.14- 2.73) Q3: 1.99 (0.29- 3.57)  Q4: 2.73 (0.76- 4.50) | Total TV mortality: Q1: 254940 (-120186- 615498)  Q2: 488028 (-50988- 994266)  Q3: 724758 (105618- 1300194) Q4: 994266 (276792- 1638900) |
| 231 | Yan et. al., 2021 | China | 4 years | TS (GLM + DLNM) | Warm season: 12994 | TV | Respiratory diseases (J00-J99) | Morbidity (HA) | 0-1 / 0-2 / 0-3 / 0-4 / 0-5 / 0-6 / 0-7 days | TV0-1: 0.7 (-1.0- 2.3) TV0-2: 8.9 (-2.3- 18.7)  TV0-3: 4.0 (0.6- 7.1) TV0-4: 3.8 (0.3- 6.9) TV0-5: 6.3 (-6.3- 17.3)  TV0-6: 4.6 (-8.2- 15.6)  TV0-7: 2.0 (-12.0- 14.1) | 94 (-128- 296)  TV0-2: 1.157 (-294- 2433) TV0-3: 518 (74- 925) TV0-4: 493 (45- 900) TV0-5: 823 (-820- 2243) TV0-6: 597 (-1066- 2032) TV0-7: 265 (-1563- 1831) |
| 240 | Yi et. al., 2019 | China | 10 years | TS (GLM + DLNM) | 36,607 | TV | Mental health disorders (F00-F99) | Morbidity (HA) | 0-7 days | Total TV0-7: 2.25 (0.79- 3.75) | Total TV0-7: 823 (291- 1374) |
| 42 | Dang et. al., 2018 | Ho Chi Minh city , Vietnam | 4 years | TS (GLM + DLNM) | 101,897 | Heat; mean temperature > 75^th^ percentile | Disorders in general | Mortality | 0-21 days | 0.42 (0.11-0.73) |  |
| 102 | Lee et. al., 2018 | Canada, USA, Brazil, Colombia, UK, Ireland, Spain, Japan, South Korea and Australia | 42 years | TS (GLM + DLNM) | Total: 85,912,372 Canada: 2,989,901 USA: 22,896,409 Brazil: 3,435,502 Colombia: 956,539 UK: 1,275,786 Ireland: 1,058,215 Spain: 3,480,531 Japan: 3,611,897 South Korea: 1,727,642 Australia: 1,177,950 | DTR | Disorders in general | Mortality | 0-14 days | Overall: 2.5 (2.3-2.7) Canada: 2.7 (1.8-3.5)  USA: 3.2 (2.9-3.5) Brazil: 3.7 (2.6-4.9) Colombia: -1.5 (-5.1-2.1)  UK: 2.1 (1.6-2.7) Ireland: 0.2 (-0.2-1.4) Spain: 4.2 (3.5-4.9) Japan 2.7 (2.4-3) South Korea: 4.5 (3-5.9) Australia: 3.3 (1.1-5.3) |  |
| 262 | Lee et. al., 2020 | 20 countries | 31 years | TS (GLM + DLNM) | Total: 93,886,489 Canada: 2,734,629 USA: 22,690,332 Mexico: 2,169,253 Brazil: 3,435,535 Argentina: 688,061 Korea: 1,671,728 Japan: 30,746,547 Taiwan: 1,162,844 Thailand: 1,801,653 Australia: 1,177,950 Finland: 130,395 Estonia: 146,347 Ireland: 1,012,684 UK: 12,075,786 Moldova: 59,906 / Switzerland: 243,638 France: 1,197,555 Spain: 3,470,738 Portugal: 966,814 South Africa: 6,304,094 | DTR | Disorders in general | Mortality | 0-14 days | Canada: 2.4 (1.5-3.3)  USA: 3.0 (2.7-3.3) Mexico: 7.4 (4.6-10.1) Brazil: 3.4 (1.8-4.9) Argentina: 5.7 (2.6-8.8) Korea: 4.2 (2.7-5.9) Japan: 2.6 (2.2-2.9) Taiwan: 2.6 (0.2-4.8) Thailand: 3.7 (2.9-4.4) Australia: 3.3 (1.1-5.4) Finland: 2.4 (-2.8-7.2) Estonia: 1.6 (-1.4-4.7) Ireland: 0.2 (-1.2-1.7)  UK: 2.3 (1.7-2.9)  Moldova: 2.1 (-5.9-10.3)  Switzerland: 0.6 (-1.3-2.5)  France: 2.1 (0.8-3.3)  Spain: 4.2 (3.5-4.9) Portugal: 3.3 (0.7-5.8)  South Africa: 2.2 (1.3-3.0) |  |
| 117 | Liu et. al., 2022 | 115 cities, USA | 55 years | TS (GLM + DLNM) | 32,125,003 | Heat; temperature anomaly > 0 | Disorders in general | Mortality | 0 week (1st week of exposure) | 0.394 (0.332-0.451) |  |
| 205 | Urban et. al., 2022 | Czech republic | 38 years | TS (GLM + DLNM) | 213,313 | Warm season; may-september | Disorders in general | Mortality | 0-10 days | 1980s: 0.90 (0.46- 1.34) 1990s: 0.73 (0.37- 1.10) 2000s: 1.03 (0.58- 1.47) 2010s: 1.75 (1.32- 2.19) | 1980s: 61 (32- 89) 1990s: 43 (20- 65) 2000s: 53 (29- 74) 2010s: 86 (64- 107) |
| 216 | Wen et. al., 2023 | France | 3 years | CC (GLM + DLNM) | 1,681,619 | 1°C increase of TV (intra and interdays, 0-1d to 0-7d) | Disorders in general | Mortality | 0-1 / 0-2 / 0-3 / 0-4 / 0-5/ 0-6 / 0-7 days | TV Lag 0–1 deaths: 0.58 (-0.05- 1.20) TVLag 0–7: 2.16 (1.35- 2.97) | TVLag 0–1 deaths: 9690 (-924- 20221) TVLag 0–7: 36369 (22624- 49977) |
| 248 | Zhang et. al., 2019 | China, UK, USA | China: 7 years, US: 14 years, UK: 23 years | TS (DLNM) | Total 23,089,328 China 93,750 UK 7,573,716 USA 15,421,862 | 1°C increase of TV (intra and interdays, 0-1d to 0-10d) | Disorders in general | Mortality | 0-1 / 0-2 / 0-3 / 0-4 / 0-5/ 0-6 / 0-7 / 0-8 / 0-9 / 0-10 days | Total TV daily related mortality lag 07 China: 3.69 (0.51- 6.75) TV daily lag 0-7d UK: 1.14 (0.54- 1.74) TV daily USA: 2.57 (1.97- 3.16) Total TV hourly related mortality lag 07 China: 2.59 (0.10- 5.01) Tvhourly lag 0-7d UK: 0.98 (0.55- 1.42) Tvhourly USA: 1.67 (1.15- 2.18)  Total TV daily related mortality lag 0-10d China: 3.69 (0.51- 6.75) TV daily lag 0-10d UK: 1.31 (0.61- 2.01) TV daily USA: 2.83 (2.13- 3.53) Total TVhourly related mortality lag 010 China: 3.03 (0.36- 5.62) TV daily lag 0-7d UK: 1.07 (0.58- 1.57) TV daily USA: 1.84 (1.27- 2.40) |  |
| 252 | Zhang et. al., 2018 | Wales, England | 14 years | TS (DLNM) | 7,573,716 | Hourly TV | Disorders in general | Mortality | 0-10 days | 2.52 (2.27- 2.76) | 190,858 (171.923- 209035) |
| 239 | Yim et. al., 2021 | 13 cities, China | 2 years | CC | 303,670 | 1°C increase of TV (intra and interdays, 0-1d to 0-4d) | Cancer (C00-D48) | Mortality | 0-1 / 0-2 / 0-3 / 0-4 days | TV0-1: 3.59 (0.98- 6.29)  TV0-2: 4.74 (1.87- 7.65)  TV0-3: 4.29 (2.02- 6.65)  TV0-4: 3.96 (1.83- 6.17) | TV0-1: 10903 (2967- 19097)  TV0-2: 14395 (5690- 23218)  TV0-3: 13041 (6122- 20181)  TV0-4: 12040 (5553- 18740) |
| 242 | Yu et. al., 2021 | 45 cities, USA | 14 years | TS (DLNM) | 15,421,862 | TV (hourly and 0-1d) | CVD (I00-I99) and respiratory diseases (J00-J99) | Mortality | 0-7 days | Total mortality TV daily: 2.55 (1.94- 3.15) / TV hourly: 1.65 (1.14- 2.15) Non-accidental mortality: TVdaily: 2.4 (1.77-3.05) TVhourly: 1.57 (1.05- 2.08) Respiratory mortality TVdaily: 3.07 (1.11- 4.99) TVhourly: 1.89 (0.43- 3.34) Cardiorespiratory TVdaily: 2.5 (1.67 -3.42) TVhourly: 1.67 (0.97- 2.37)  CVD TVdaily: 2.43 (1.42- 3.43) TVhourly 1.63 (0.82- 2.43) | Total mortality TV daily: 393257 (299184- 485789) TV hourly: 254461 (175809- 331570) |
| 254 | Zhao et. al., 2018 | Brazil | 16 years | TS (linear regression model) | 147,959,243 | TV (0-1d) | Disorders in general | Morbidity (HA) | 0-1 day | 3.5 (3.1- 3.8) | 5,141,816 (4646015, 5635099) |
| 74 | He et. al., 2021 | Anqing ,China | 5 years | TS (GLM + DLNM) | 8,675 | High mean temperature; 99^th^ percentile High DTR; 75^th^ percentile  TCN; > 5^th^ | Musculoskeletal system (M00-M99) | Morbidity (HA) | TCN: 0-7 days Mean temperature 0-16 days DTR: 0-21 days | High mean Temperature: 14.93 (5.99-22.11) DTR: 4.19 (-27.68-29.18) TCN: 4.01(-1.49-9.63) | High mean Temperature: 1,294,893 DTR: 362,6718 TCN: 347,7306 |
| 80 | Huang et. al., 2020 | Hefei, China | 5 years | TS (GLM + DLNM) | 28,544 | TCN and DTR | Infectious diseases (A00-B99) | Morbidity (HA) | 0-7 days | TCN Overall: 2.79 (-1.42-6.73) DTR overall: 13.09 (5.47-19.90) | TCN Overall: 800  DTR overall: 3445 |
| 81 | Huang et. al., 2023 | 23 sites, China | 6 years | TS (GLM + DLNM) | Total: 96,092  Age 15-64: 67,004 ≥65: 28,045 Urolithiasis: 26,369 Renal failure: 22,544 Acute kidney injury: 717 Chronic kidney injury: 18,571 Urinary tract infections: 16,092 Lower urinary tract infection: 13,126 | Heatwave; mean temperature exceeding the 90^th^, 95^th^, or 97.5^th^ percentile for > 2, 3, or 4 consecutive days | Genitourinary diseases (N00-N99) | Morbidity (HA) |  | Total: 8.3 (48- 11.6) Male: 9.3 (5.5-13) Female: 5.8 (-0.9-12.2) Urolithiasis: 16.6 (9.1- 23.5) Renal failure: 3.5 (−5.9- 11.9) Chronic kidney disease: −1.5 (−12.2- 8.2) Urinary tract infections: 0.16 (−6.8- -6.6) Lower urinary tract infections: −0.9 (−17.9- 13.6) | Total: 1463 (844- 2059) Male: 954 (564- 1328) Female: 436 (-71- 909) 15-64: 1036 (481- 1565) 65≥: 380 (99- 645) Urolithiasis: 818 (449- 1156) |
| 135 | Ma et. al., 2021 | 2,669 counties, USA | 9 months | TS (GLM + DLNM) |  | Heat; mean temperature > 32.2°C | Infectious diseases (A00-B99) | Morbidity (prevalence) |  | 3.73 (3.66-3.76) |  |
| 105 | Lei et. al., 2020 | Shenzhen, China | 12 years | TS (GLM + DLNM) | Total: 142,569 male 88,709 female: 55,860 age <40: 11,624  age 40-64: 71,493  age ≥65: 59,452 education primary and below: 45,805 education junior high school: 56,388 education senior high school and above: 40,376 cerebral infarction: 104,339 ICH: 30,656 | DTR | Stroke (I60-I69) | Morbidity (HA) | 0-5 days | Summer: total 3.65 (1.81-5.53) male 3.90 (1.81-5.71) female: 3.23 (0.73-5.45)  age <40: 1.79 (-2.40-5.46) age 40-64: 3.59 (1.66-5.45)  age ≥65: 4.06 (1.56-6.52) primary and below: 4.13 (1.32-6.66) Junior high school: 4.26 (1.78-6.55) senior high school and above: 2.42 (0.13-4.52) cerebral infarction: 4.27 (2.26-6.10)  ICH 1.47 (-1.50-3.99) | Summer: total: 1276 (622-1880) male: 832 (420-1227) female: 438 (103-750) age <40: 52 (-74-159) age 40-64: 636 (260-972)  age ≥65: 581 (228-899) education primary and below: 473 (173-755) junior high school: 578 (239-900) senior high school and above: 240 (9-446) cerebral infarction: 1127 (570-1616)  ICH 99 (-90-283) |
| 118 | Liu et. al., 2022 | Jining, China | 7 years | TS (GLM + DLNM) | Total: 5,779 / male: 2,763 / female: 3,016 / <40: 2,922 / ≥40: 2,857 / married: 3,917 / unmarried: 1,862 | Heatwave; at least 2 days of mean temperature ≥ 95^th^ percentile EP ≥ 95^th^ percentile | Mental health disorders (F00-F99) | Morbidity (HA) | 0-14 days | Heatwave: total 0.03 (0.004-0.06) Male 0.03 (-0.01-0.06) Female 0.04 (-0.02-0.08) <40 years old 0.03 (-0.03-0.07) / ≥40 years old 0.04 (0.002-0.07) Married 0.04 (0.001-0.07 Unmarried 0.03 (-0.02-0.06) EP: Total 0.022 (-0.015- 0.06) Male 0.03 (-0.03-0.08) Female 0.02 (-0.03-0.06) / <40 years old -0.008 (-0.06-0.04) / ≥40 years old 0.05 (0.001-0.096) Married 0.015 (-0.011-0.038) Unmarried 0.03 (-0.094-0.142) | Heatwave: total 1.845 (0.220-3.329) male: 0.730 (-0.377-1.698) female: 1.102 (-0.439-2.464) <40: 0.747 (-0.830-2.110)  ≥40: 1.042 (0.049,1.910) married: 1.399 (0.014-2.636) unmarried: 0.489 (-0.291-1.175) EP: total 1.262 (-0.838-3.265) male: 0.829 (-0.801-2.321) female: 0.452 (-0.955-1.774) <40: -0.239 (-1.88-1.281) ≥40: 1.429 (0.038-2.733) married 0.575 (-0.411-1.495) unmarried: 0.549 (-1.744-2.65) |
| 243 | Zhan et. al., 2020 | China | 3 years | TS (DLNM) | 71,070 | TV | Respiratory disease (J00-J99) | Morbidity (HA) | 0-6 days | Total hourly COPD hospitalizations: 12.0 (6.5- 17.1) Total daily COPD hospitalizations: 15.4 (9.2- 21.1) | Total hourly COPD hospitalizations: 85000 (46000- 121000) Total daily COPD hospitalizations: 109000 (65000- 150000) |
| 253 | Zhao et. al., 2018 | Brazil | 16 years | CC (linear lag model) | 447,667 | TV (0-1d) | CVD (I00-I99) | Morbidity (HA) | 0-21 days | Total arrythmia hospitalizations: 8. (4.1- 11.5) | Total arrythmia hospitalizations: 35813 (18302- 51665) |
| 71 | Han et al., 2020 | Beijing, China | 8 years | TS (GAM) | Total: 340,833 Total: 60,698 (respiratory deaths), 280,135 (circulatory deaths) | Heat; > MMT PM2.5 | CVD (I00-I99) and respiratory diseases (J00-J99) | Mortality | 0-3 days | Heat:  Respiratory: 1.86 (0.79-2.86)  Circulatory: 1.14 (0.64-1.62)  PM2.5:  Respiratory: 5.45 (3.30-7.56)  Circulatory: 3.82 (2.79-4.83) | Heat:  Respiratory: 1130 (478-1734)  Circulatory: 3190 (1782-4551)  PM2.5:  Respiratory: 3308 (2000-4587)  Circulatory: 10 699 (7816-13 530) |
| 114 | Lin et. al., 2016 | 6 cities, China | 3 years | TS (GLM) | 316,305 | PM2.5; > WHO recommended concentration (25μg/m3) | Disorders in general | Mortality | 0-3 days | 3.79 (3.14-4.45) | 11,176 (9,261-13,120) |
| 89 | Jiao et. al., 2019 | China | 3 years | CC (DLNM) | 503,436 | PM2.5; > WHO recommended concentration (25μg/m3) | Infectious diseases (A00-B99) | Morbidity (EDV) | 0-10 days | Total mean PM2.5 related EDVs: 1.09 (0.69- 1.49) DECH related: 2.02 (1.42- 2.61) | Total mean PM2.5 related EDVs: 5034,36 5487 (3474- 7501)  DECH related: 10169 (7149- 13140) |
| 87 | Ji et. al., 2022 | Qingdao, China | 5 years | TS (GLM + DLNM) | 54,966 | PM2.5, PM10, SO2; WHO air quality guidelines, China grade II standard for air quality, 50% China grade II | Mental health disorders (F00-F99) | Morbidity (HA) | PM2.5: 0-6 days  PM10 0-5 days  SO2 0-5 days NO2 0-5 days CO 0-5 days | Total (all air pollutants): 12.41 (4.63-19.31) PM2.5: 3.19 (0.81-5.40) PM10: 3.56 (0.67-6.25)  SO2: 5.66 (3.15-7.66). | Total (all air pollutants): 1352 (504-2103) PM2.5: 348 (88-588) PM10: 388 (73-681) SO2: 616 (343-834) |
| 107 | Li et. al., 2022 | China (Guangzhou) | 5 years | TS (GLM + DLNM) | Total: 7,916 | Tropical cyclones;  Absence of tropical cyclones | Infectious diseases (A00-B99) | Morbidity (Incidence) | 0-4 weeks | Total: 6.31% (1.96–10.16%) | - |
| 138 | Ma et. al., 2021 | China (Chongqing region) | 12 years | TS (GLM + DLNM) | Total: 45,691  Males: 23,757  Females: 21,934 | Floods;  Absence of flood | Infectious diseases (A00-B99) | Morbidity (Incidence) | 0-7 days | Total: 1.10 (0.62-1.50) Male: 1.36 (0.80-1.92)  Female: 0.79 (0.22-1.35)  Age 0-4: 1.15 (0.15-1.46)  Age 5-14: 0.94 (-0.60-2.20)  Age 15-64: 1.10 (0.75-2.03)  Age >/65: 0.89 (-0.45-1.83) Student: 0.96 (0.53-2.63)  Farmer: 1.15 (-1.92-2.19)  Worker: 1.12 (0.16-2.69)  Child: 1.14 (0.22-1.56)  Other: 1.04 (0.43-1.73) | Total: 497 (294-605)  Male: 324 (187-453)  Female: 173 (40-296)  Age 0-4: 220 (34-284)  Age 5-14: 28 (-17-63)  Age 15-64: 211 (140-388)  Age >/65: 37 (-16-79)  Student: 42 (19-120)  Farmer: 22 (-38-44)  Worker: 43 (5-106)  Child: 227 (41-313)  Other: 161 (62-274) |
| 99 | Lan et. al., 2022 | China (Sichuan province) | 3 years | TS (GLM + DLNM) | Total: 124,602 | Flood; Absence of flood | Infectious diseases (A00-B99) | Morbidity (Incidence) | 0-14 days | Total: 0.25% (0.10-0.36) | Total: 310 (123-454) |
| 88 | Jiang et. al., 2022 | China (Suzhou city) | 3 years | TS (GLM + DLNM) | Total: 26,343  Male: 15,844  Female: 10,499  Age ≤ 18: 2198  Age 19-39: 8083  Age 40-64: 10,889  Age ≥ 65 years: 5173  First visit: 12,567  Multiple visits: 13,776 | Extreme precipitation (≥ 95th percentile); Absence of precipitation | Mental health disorders (F00-F99) | Morbidity (OV) | single-day lag 5 | Total: 5.00% (1.02 - 8.82)  Male: 3.94% (-0.43-8.13)  Female: 6.58% (1.33-11.56)  Age 0-18: 6.15% (-9.58-19.63)  Age 19-39: 4.45% (0.12-8.59)  Age 40-64: 3.06% (-0.09-6.12)  Age ≥65: 9.37% (1.11-16.94)  First visit: 4.92% (-0.66-10.2)  Multiple visits: 5.04% (1.01-8.9) | Total: 1318  Male: 625  Female: 691  Age 0-18: 135  Age 19-39: 360  Age 40-64: 334  Age ≥65: 485  First visit: 618  Multiple visits: 695 |
| 29 | Chen et al., 2022 | China (Beijing) | 6 years | TS (GLM + DLNM) | Total Acute Myocardial Infarction (AMI): 124,760 Male: 86,176 Female: 38,584 Age 20-64 years: 57,813 Age ≥ 65 years: 66,947 | Extreme precipitation;  ≥ 95th percentile of the cumulative daily precipitation | CVD (I00-I99) | Morbidity (HA) | 0-14 days | Total: 0.68 (0.20%, 1.12%) Male: 0.50 (−0.10%, 1.02%) Female: 1.06 (0.70%, 1.67%) Age 20-64 years: 0.49 (−0.24%, 1.12%)  Age ≥ 65 years: 0.82 (0.22%, 1.34%) | Total: 854 (244, 1395) Male: 435 (−85, 883) Female: 409 (271, 646) Age 20-64 years: 283 (−137, 646) Age ≥ 65 years: 551 (146, 895) |
| 249 | Zhang et. al., 2023 | Australia (New South Wales) | 4 years | Cohort study - cox proportional hazards regression models | Preterm births: 24,034  Low birth weight: 7,160 | Per interquartile range rise in PM2.5 due to wildfires | Diseases originating in the perinatal period (P00-P96) | Morbidity (Incidence) | - | Preterm births: 14.30 (12.22-16.32)  Low birth weight: 8.04 (3.26-12.43) | Preterm births: 4434 (3789-5060)  Low birth weight: 619 (251-957) |
| 241 | Yu et. al., 2021 | Brazil | 14 years | Difference-in-difference (GLM) | Total: 1502358 (Cancer hospitalizations) | PM 2.5;  nations lowest PM 2.5 | Neoplasms (C00-D48) | Morbidity (HA) | 0-1 years | Total cancer hospitalizations: 33.82% (14.97, 47.84) | Total cancer hospitalizations: 508097 (224903, 718728) |
| 224 | Wu et. al., 2023 | China | 4 years | TS (GLM + DLNM) | Total: 4028 (admissions) | NO2, O3, PM10 | Dermatopolymyositis (M33) | Morbidity (HA) | 0-7 days | Total NO2 related DM hospitalizations: 2.4% (0.6, 3.8)  Total O3 related DM hospitalizations: −1.5% (−3.9, −0.2)  Total PM10 related hospitalizations: 0.2% (−1.1, 1.0) | Total NO2 related DM hospitalizations: 96.5 (18.0, 151.0)  Total O3 related DM hospitalizations: −61.5 (−158.0, −10.0)  Total PM10 related hospitalizations: 8.4 (−45.0, 38.0) |
| 219 | Wu et. al., 2022 | China (Shandong Province) | 6 years | TS (GLM + DLNM) | Total: 2,193,954  Ischemic: 1,861,331  Hemorrhagic: 332,623 | 10ug/m3 daily increase in PM1, PM2.5, PM10;  Cross-validation coefficient of determination (CV-R2) of 0.83 and a root-mean-square error (RMSE) of 10.86 μg/m3 for PM1, CV-R2 of 0.91 and RMSE of 12.67μg/m3 for PM2.5, CV-R2 of 0.86 and RMSE of 24.34μg/m3 for PM10, CV-R2 of 0.84 and RMSE of 10.07μg/m3 for SO2, and CV-R2 of 0.87 and RMSE of 17.10μg/m3 for O3 on a daily basis | Stroke (I60-I69) | Morbidity (CDC (all clinics, etc.)) | 0-4 days | Total stroke: PM1: 6.9% (5.1-8.5)  PM2.5: 5.6% (4.2-6.8)  PM10: 5.6% (3.9-7.1)  Ischemic stroke: PM1: 7.6 (5.9, 9.1)  PM2.5: 6.0 (4.7,7.2)  PM10: 6.0 (4.3, 7.4)  Hemorrhagic stroke: PM1: 3.2 (−1.7,6.8)  PM2.5: 3.4 (−0.1, 6.1)  PM10: 3.1 ( −1.1, 6.2) | Total stroke: PM1: 32,406 (24,073, 39,543)  PM2.5: 26,022 (19,645, 31,684)  PM10: 26,127 (18,053, 33,164)  Ischemic: PM1: 30,096 (23,349, 35,874)  PM2.5: 23,881 (18,481, 28,660)  PM10: 23,676 (16,972, 29,516)  Hemorrhagic  PM1: 2281 (−1225,4850)  PM2.5: 2418 ( −71, 4354)  PM10: 2178 (−752, 4433) |
| 204 | Ugalde-Resano et. al., 2022 | Mexico | 4 years | TS (DLNM) | Total: 48891 (CVD-EDV) | PM10 (0-5d), PM2.5 (0-6d), O3 (0-5d), NO2 (0-4d), CO (0d);  Extended version of the counterfactual scenario | CVD (I00-I99) | Morbidity (EDV) | CEDV:  PM10: 0-5 days  PM2.5: 0-6 days  O3: 0-5 days  NO2: 0-4 days  CO: 0 day  High blood pressure: PM10: 0-5 days  PM: 2.5 days  NO2: 0-2 days  Cerebrovascular accident: O3: 0 day  CO: 0 day | Total CVD EDV:  PM10 Lag 0–5: 10.3% (2.5, 18.0)  PM2.5 Lag 0–6: 9.7% (2.3, 16.1)  O3 Lag 0–5: 10.3% (1.1, 19.5)  NO2 Lag 0–4: 11.0% (1.3, 20.0)  CO Lag 0: 5.7% (0.3, 10.7) | Total CVD EDV  PM10 Lag 0–5: 5032 (1235, 8792)  PM2.5 Lag 0–6: 4723 (1118, 7935)  O3 Lag 0–5: 5050 (548, 9555)  NO2 Lag 0–4: 5366 (641, 9776)  CO Lag 0: 2766 (145, 5254) |
| 185 | Sepandi et. al., 2021 | Iran | 4 years | TS (DLNM) | Total: around 69000 CVD EDV | CO, O3, No2, So2, Pm2.5;  25th percentile of each pollutant | CVD (I00-I99) | Morbidity (EDV) | 0-14 days | Total CO related CV EDVs: 0.054% (0.023, 0.083)  O3: 0.008% (-0.053, 0.060)  NO2: 0.04% (0.004, 0.081)  SO2: 0.004% (0.030, 0.033)  PM2.5: 0.035% (-0.010, 0.079) | - |
| 175 | Qu et al., 2019 | China (Shijiazhuang) | 4 years | TS (DLNM) | AECOPD: 9,358 | PM2.5, PM10, NO2, SO2, and CO;  75μg/m3 for PM2.5  150μg/m3 for PM10 150μg/m3 for SO2 80μg/m3 for NO2 4μg/m3 for CO | Respiratory diseases (J00-J99) | Morbidity (HA) | 0-7 days | PM2.5: 13 (5.5–20.4) PM10: 9.4 (− 1.3–18.4) SO2: 1.7 (− 10.9–12.6) NO2: 9.7 (− 3.4–21.2) CO: 8.8 (− 0.6–17.3) | PM2.5: 1,220 PM10: 879 SO2: 156 NO2: 905 CO: 828 |
| 116 | Liu et. al., 2019 | China (Shijiazhuang city) | 4 years | TS (GLM + DLNM) | Total: 4,045  Heating season: 1815  Non-heating season: 2230 | PM2.5, PM10, SO2, NO2, CO and O3;  Chinese National Ambient Air Quality Standards grade II | Respiratory diseases (J00-J99) | Morbidity (HA) | 0-7 days | Heating season  NO2: 10% (5.8-13.7)  SO2: 9.3% (5.2-12.7)  PM2.5: 8.6 (5.4-11.7)  PM10: 5.3 (1.4-8.9)  CO: 2.6 (-1.7-6.2) / O3: -3.5 (-5.2- -2.2)  Non-heating season  NO2: 7.3% (2.2-12.2)  SO2: 7.9% (54.5-11.6)  PM2.5: 8.3% (3.1-13.2)  PM10: 11.3% (4.9-17)  CO: 5.7% (2.9-8.8)  O3: 7% (-0.3-13.7) | - |
| - | Jaffe et. al., 2003 | USA | 6 years | TS (GLM) | Total: 5416 | Air pollutants (NO2, O3, PM10, SO) exposure | Respiratory diseases (J00-J99) | Morbidity (EDV) | - | Cincinnati NO2 0.72  O3 0.60  PM10 --  SO 4.20  Cleveland NO2 0.44  O3 0.11  PM10 1.32  SO 0.66  Columbus NO2 –  O3 0.57  PM10 3.62  SO 2.94 | - |
| 79 | Hu et. al., 2018 | China (Zhejiang) | 5 years | TS (GLM + DLNM) | Total: 1,008,515  CVD 335,348  Stroke 188,977  Respiratory 145,181  COPD 92,888 | Air pollutants (PM1, PM2.5, PM10) exposure | Respiratory diseases (J00-J99)  CVD (I00-I99)  Stroke (I60-I69) | - | 0-2 days | PM1: all cause 2.39 (1.28-3.48)  Age 0-64: 1.59 (0.23-2.92)  Age ≥65: 2.64 (1.27-3.97)  Female: 3.12 (1.21-4.98)  Male: 1.83 (0.83-2.82)  CVD: 2.86 (0.85-4.81)  Stroke: 2.87 (0.86-4.83)  Respiratory: 2.14 (0.00-4.22)  COPD: 2.83 (0.55-5.05)  PM2.5: all cause 2.53 (1.42-3.63)  Age 0-64: 1.73 (0.34-3.10)  Age ≥65: 2.78 (1.39-4.14)  Female: 3.32 (1.32-5.26)  Male: 1.93 (0.93-2.92)  CVD: 3.02 (0.94-5.04)  Stroke: 2.98 (0.89-5.01)  Respiratory: 2.22 (0.06-4,31)  COPD: 2.89 (0.58-5.13)  PM10: all cause 3.08 (1.95-4.19)  Age 0-64: 1.98 (0.53-3.40)  Age ≥ 65: 3.32 (1.89-4.72)  Female: 3.88 (1.83-5.88)  Male: 2.07 (1.03-3.08)  CVD: 3.54 (1.42-5.59)  Stroke: 3.46 (1.24-5.61)  Respiratory: 2.60 (0.65-4.49)  COPD 3.31 (1.16-5.40) | - |
| 40 | Dabrowieck et. al., 2022 | Poland (Tricity, Warsaw and Cracow) | 9 years | TS (GLM + DLNM) | Total: 31,919 | Air pollutants (PM2.5, PM10, NO2, SO2) exposure | Respiratory diseases (J00-J99) | Morbidity (HA) | 0-7 days | PM10: 4.52% (0.80%–8.14%); PM2.5: 3.74% (0.29%–7.11%); NO2: 16.4% (10.0%–21.8%); NO2: 2.50% (−0.75%–5.36%) | - |
| 30 | Chen et. al., 2022 | China (Hefei) | 6 years | TS (GLM + DLNM) | Total: 4501 | Air pollutants (PM2.5, NO2, O3) exposure | Diseases of the musculoskeletal system and connective tissue (M00-M99) | Morbidity (OV) | NO2: 0-10 / PM2.5 0-14 / O3 0-27 days | PM2.5: 1.67(0.30-2.07)  NO2: 5.16 (2.55-7.21)  O3: -6.38 (-14.53- -0.41) | PM2.5: 75 (12-93)  NO2: 232 (119-324)  O3: -287 (-644- -30) |
| 238 | Ye et. al., 2021 | Brazil | 16 years | TS (GLM + DLNM) | Total: 143586178 | Wildfire specific PM2.5 | Disorders in general | Morbidity (HA) | 0-1 days | Total HA: 0.53% (0.48, 0.58) | Total HA: 766091 (692740, 839301) |
| 73 | He et. al., 2018 | China (Hebei) | 1 year | Poisson age-period-cohort model (GAM) | Total: 30,590 | Air pollutant (PM2.5) exposure | Respiratory diseases (J00-J99) | Mortality | 10 years | Total: 8.3 (7.4-9.1)  Men: 5.7 (2.8-9.4)  Women: 16.7 (8.3-21.6)  Age 30-65: 6.5 (4.7-10.3)  Age ≥ 65: 9.1 (6.4-11.5) | Total: 2525 (2265-2780)  Men: 1165 (582-1942)  Women: 1667 (830-2167)  Age 30-65: 564 (403-888)  Age ≥ 65: 1995 (1396-2525) |
| 8 | Allen et. al., 2013 | Mongolia (Ulaanbaatar) | 1 year | TS (stepwise multiple linear regression model) | Cardiopulmonary disease: 2,154  Lung cancer: 117 | Air pollutant (PM2.5) exposure | Respiratory diseases (J00-J99)  CVD (I00-I99)  Neoplasms (C00-D48) | Mortality | - | Cardiopulmonary: 29 (12-43)  Lung cancer: 40 (17-56) | Cardiopulmonary: 578 (232–85)  Lung cancer: 45 (19–64) |
| 213 | Wang et al., 2020 | China (Wuhan) | 3 years | TS (GAM) | Total: 45,714 IHD: 9,495 Stroke: 17,504 | Air pollutants (PM2.5 and PM10) | CVD (I00-I99) | Morbidity (Hospitalization) | 0-7 days | PM2.5 highest at lag7 day:  CVD: 1.01% (0.67–1.34), no gender difference, age 0-64 years (1.05% (0.53-1.56)) more vulnerable than 65+ years (0.97% (0.53-1.42)) IHD: 1.10% (0.37–1.84)  Stroke: 1.01% (0.45–1.56) PM10 effect highest at lag 7 day: CVD: 0.48% (0.26–0.70), males (0.51% (0.22-0.79)) more vulnerable than females (0.44% (0.11-0.77)), age 0-64 years (0.57% (0.24-0.91)) more vulnerable than 65+ years (0.41% (0.11-0.69)) IHD: 0.58% (0.11–1.07) Stroke: 0.61% (0.25–0.96) | PM2.5: CVD: 1487 (1007, 1956)  IHD: 340 (119, 549) Stroke: 550 (245, 834) Males: 855 (777, 932) Females: 653 (335, 959) 0-64 years: 653 (339, 955) 65+ years: 1118 (1035, 1199) PM10: CVD: 983 (541, 1415) IHD: 249 (49, 441) Stroke: 465 (199, 722) Males: 460 (388, 531) Females: 390 (95, 675) 0-64 years: 497 (212, 773) 65+ years: 471 (393, 548) |
| 174 | Qiu et. al., 2018 | China | 2 years | TS (GAM) | Total: 10947  Dementia: 920  Schizophrenia: 3368 Depression: 1193 | Air pollutants (PM2.5, PM10, PMc) | Mental Health Disorders (F00-F99) | Morbidity (HA) | 0-6 days | Total Mental disorder HA for PM2.5: 9.53% (2.67, 15.58)  for PM10: 9.17% (2.91, 14.7)  for PMc: 6.1% (1.4, 10.32)  Dementia HA for PM2.5: 0% (0,0)  for PM10: 0% (0,0)  for PMc: 7.22% (0.63, 12.81)  Schizophrenia HA for PM2.5: 15.13% (1.83, 25.4)  for PM10: 15.32% (3.49, 24.56)  for PMc: 11.69% (3.41, 18.42)  Depression HA for PM2.5: 12.07% (0.1, 21.81)  for PM10: 0% (0, 0)  for PMc: 0% (0, 0) | Total Mental disorder HA for PM2.5: 1044 (293, 1705)  for PM10: 1004 (319, 1609)  for PMc: 668 (153, 1129)  Dementia HA for PM2.5: 0 (0,0)  for PM10: 0 (0,0)  for PMc: 66 (6118?)  Schizophrenia HA for PM2.5: 510 (62, 856)  for PM10: 516 (118, 827)  for PMc: 394 (115, 621)  Depression HA for PM2.5: 144  for PM10: 0 (0, 0)  for PMc: 0 (0, 0) |
| 173 | Qiu et al., 2020 | China | 2 years | TS (GAM) | Total: 4,407,601 | Air pollutants (PM2.5 and PM10) | Respiratory diseases (J00-J99)  CVD (I00-I99)  Diseases of the genitourinary system (N00-N99)  Diseases of the nervous system (G00-G99) | Morbidity (Hospitalization) | 0-1 days | Non-accidental hospitalization: 0.70 (0.35, 1.06) Circulatory diseases: 0.88 (0.37, 1.39) Respiratory diseases: 1.69 (1.12, 2.24) Endocrine diseases: 1.10 (0.49, 1.71) Nervous system diseases: 0.68 (0.12, 1.22) Genitourinary diseases: 0.81 (0.41, 1.20) | Non-accidental hospitalization: 32667 (16065, 49065) Circulatory diseases: 5771 (2421, 9063) Respiratory diseases: 15659 (10430, 20792) Endocrine diseases: 1469 (647, 2273) Nervous system diseases: 983 (180, 1771) Genitourinary diseases: 2974 (1526, 4401) |
| 172 | Qiu et. al., 2018 | China | 2 years | TS, over disperse poisson distribution | Total: 54966 | Air pollutants (PM2.5, PM10, SO2) | Respiratory diseases (J00-J99) | Morbidity (HA) | PM2.5: 0-6 days  PM10: 0-5 days  SO2: 0-5 days  NO2: 0-5 days  CO: 0-5 days | Total PM2.5 related COPD HA reference WHO: 7.33% (4.31, 10.34)  PM10: 6.26 % (3.66, 8.86)  SO2: 0.32% (0.19, 0.45) | Total PM2.5 related COPD HA reference WHO: 3987 (2348, 5625)  PM10: 3411 (1995, 4829)  SO2: 175 (103, 247) |

| 261 | Aboubakri et al., 2023 | Iran | 2008-2014 (7 years) 2015-2022 (8 years) | Time-series, GAM, DLNM | - | Heat: Tmean> MMT, extreme heat: T > 95th percentile, Comparison: MMT | All-cause | Mortality | 0-21 days | Strongest result: Baneh: heat: 0.38% (0.11, 0.52), extreme heat: 0.031% (-0.004, 0.052) | - |
| --- | --- | --- | --- | --- | --- | --- | --- | --- | --- | --- | --- |
| 262 | Corvetto et al., 2024 | Brazil | 2017-2021 (5 years) | Time-series, GLM, DLNM | Emergency departement visits: 256,406 | Moderate heat: 99th > Tmean > 90th percentile Extreme heat: Tmean ≥ 99th percentile  Comparison: 50th percentile | Mental health diseases | Morbidity (emergency departement visits) | 0-6 days | Total: moderate heat: 0.36% (-0.43, 1.13), extreme heat: 0.33% (0.16, 0.50) | Total: moderate heat: 931 (-), extreme heat: 859 (-) |
| 263 | He et al., 2023 | China | 2013-2019 (7 years) | Time-stratified case-crossover, conditional logistic regression model, DLNM | Total deaths: 236,987 | Heat: Tmean > MMT Comparison: MMT | Pneumonia | Mortality | 0-21 days | Influenza-related pneumonia: 1.31% (-0.22, 2.65), viral pneumonia: 1.79% (0.96, 2.60), bacterial pneumonia: 1.96% (1.25, 2.65) | - |
| 266 | Huang et al., 2023 | China | 2014-2019 (5 years) | Time-series, quasi-Poisson regression, DNLM | Hospital admissions in cold season: 2,350,223, hosital admissions in hot season: 2,415,112 | Temperture variability: inter-day (1 day) temperature difference. Comparion: 50th percentile of temperature variability (overal) | Non-specific, cause-specific | Morbidity (hospital admissions) | Cold season: 5-14 days, warm season: 0-10 days | Total: 2.05% (-0.90, 4.53); Age: <14y: -6.24% (-17.87, 1.86), 15-64y: 3.66% (-0.88, 6.94), >65y: 1.94% (-1.47, 4.49); Gender: male: 3.81% (0.52, 6.52), female: 0.52% (-1.89, 2.84); Disease groups: mental and behavioral nervous: 3.22% (0.25, 5.43), CVDs: 9.18% (2.03, 13.90), respiratory: 3.57% (-4.80, 9.74), digestive: 0.91% (-3.07, 3.57), musculoskeletal: 6.24% (-2.28, 10.69), genitourinary: 7.46% (1.48, 10.88) | - |
| 268 | Wang et al., 2023 | China | 2011-2020 (10 years) | Time-series, GLM, DLNM | Total new cases: 849,676 | PM2.5: Interquartile Range (IQR) increase of weekly average Comparison: minimum TB-risk PM2.5 | Tuberculosis | Morbidity (incidence) | 0-6 weeks | Total: 20.00% (6.82, 29.98) | Total: 169,595 (57,981, 254,744) |
| 269 | López-Bueno et al., 2023 | Spain | 2013-2018 (6 years) | Time-series, GLM | Hospital admission due to kidney-related diseases: 30,368 | Extreme heat: Tmax > 35.6°C Air pollution: PM10, PM2.5, NO2, O3  Comparison: Heat: 35.6°C, Air pollution: WHO 24h guidelines | Kidney-related issues | Morbidity (hospital admissions) | 0-5 days | Kidney diseases: extreme heat: 11% (8.6, 13.4); Acute kidney injury: extreme heat: 13% (10.2, 15.6), NO2: 2.1% (0.6, 3.7), O3: 16.8% (1.1, 29.9); Urolithiasis: extreme heat: 5.3% (1.6, 8.9), PM10: 9.5% (0.8, 17.5); Dysnatremia: extreme heat: 28.6% (25.6, 31.4), O3: 22.4% (2, 38.5); Hypovolaemia: extreme heat: 27% (22.8, 31) | - |
| 270 | Schulte et al., 2024 | Switzerland | 1998-2019 ( 20 years (warm season)) | Time-series, GLM, DLNM | Total emergency departement visits: 2,884,489 | Heat: Tmax > MMT Comparison: MMT | Non-external causes, external causes, cause-specific | Morbidity (emergency departement visits) | 0-7 days | Non-specific: 1.1% (0.7, 1.4), non-external cause: 0.3% (0.1, 0.6), external cause: 4.2% (3.5, 4.8); Cause-specific: Digestive system 0.0% (-0.1, 0.2), mental and behavioral disorders: 3% (2, 4.1), respiratory disorder: 0.4% (-0.4, 1.1), genitourinary system: 2.1% (0.7, 3.4), infectious diseases: 4.7% (3.3, 6), nervous system: 0.4% (0.0, 0.8), urinary tract infections: 1.2% (0.2, 2.3), kidney stones: 8.3% (5.6, 10.7), acute kidney injury: 8.2% (2, 13.2), dehydration: 29.7% (24.8, 33.7), chronic kidney disease: 7.1% (0.2, 12.7) | All-cause: 31,387 (21,567, 40,408), non-external cause: 7,906 (1,760, 13,849), external cause: 23,962 (19,901, 27,688) |
| 273 | Gonçalves et al., 2023 | Brazil | 2020-2021 (2 years) | Time-series, GLM, DLNM | Cases: 1,308,711, deaths: 37,263 deaths, severe cases: 104,875 | Increase of 1μg/m3 of PM2.5 | COVID-19 | Mortality + Morbidity (incidence) | 0-7 days | Total calculated cases: 4.24%, SD: 0.0377, deaths: 3.41%, SD: 0.1420, severe cases: 3.34%, SD: 0.0890 | - |
| 274 | Zhang et al., 2024 | Multi country (Australia, Brazil, Canada, Chile, New Zealand, Thailand, Taiwan) | 2000-2019 (Different periods) (20 years) | Time-series, GLM, DLNM | Hospitalizations due to diabetes (all types): 3,612,135 | Wildfire specific (WS) PM2.5 Comparison: Absence of WS PM2.5 | Diabetes (all) | Morbidity (hospital admissions) | 0-3 days | All-diabetes types: 0.67% (0.16, 1.18); Type 1 diabetes: 0.82% (-0.04, 1.67), type 2 diabetes: 1.02% (0.20, 1.81), malnutrition-related: -2.28% (-12.15, 5.55), other specified diabetes: 1.14% (-2.19, 3.60), unspecified diabetes: 0.48% (-0.66, 1.26) | Diabetes (all-cause): 23,544 (5459, 41,281), type 1 diabetes: 7270 (-390, 14,691), type 2 diabetes: 15,215 (2931, 27,094), malnutrition-related: -494 (-2638, 1205), other specified diabetes: 1447 (-2782, 4574), unspecified diabetes: 5148 (-7132, 13,576) |
| 275 | Wang et al., 2023 | China | 2015-2018 (4 years (warm season)) | Time-series, DLNM | Hospital admissions due to chronic kidney diseases: 768,129 | High temperature: daily Tmean during hot season No comparison, data per per 1° increase of daily mean temperature in hot season | Chronic kidney disease | Morbidity (hospital admissions) | 0-7 days | Total: 5.5% (-13.5, 20.7) | - |
| 276 | Qiu et al., 2023 | China | 2014-2017 (4 years) | Time-to-event design, cox proportional hazards regression, DLNM | Pre-term birth cases: 251,257 | Extreme heat: Tmean > 95th percentile Comparison: 50th percentile | Pre-term birth (<37 gestational weeks) | Morbidity (cases) | - | 3rd trimester: normal weight: 3.17% (2.58, 3.79), underweight: 3.14% (2.24, 4.18), oberweight/obese: 3.31% (2.01, 4.88) | 3rd trimester: normal weight: 351 (286, 420), underweight: 66 (47, 88), oberweight/obese: 69 (42, 103) |
| 277 | Wang et al., 2023 | China | 2014-2016 (3 years) | Time-series, GLM, DLNM | New infections due to diarrhea: 471,871 | Extreme heat: Tmean ≥ 97.5th percentile Comparison: 50th percentile | Infectious diarrhea | Morbidity (incidence) | 0-30 days | Total: 0.99% (0.57, 1.29) | - |
| 278 | Zhou et al., 2023 | China | 2000-2020 (21 years) | Time-stratified case-crossover, DLNM | Hospitalizations due to urolithiasis: 118,180 | Wet-bulb globe temperature (WBGT) > MMT Comparison: MMT | Urolithiasis | Morbidity (hospital admissions) | 0-7 days | 2010-2019: 10.1% (6.6, 13.2) | - |
| 280 | Yang et al., 2023 | Multi country (35 countries) | 2000-2016 (Different periods) (17 years) | Time-series, GLM, DLNM | All-cause deaths: 47,600,000 | Floods: days with flood Comparison: non-flood days | All-cause, CVDs, respiratory | Mortality | 0-60 days | CVDs: 0.18% (-), respiratory: 0.41% (-) Calculated mean and SD without extreme values:  All-cause: −0.019% (SD: 0.070), CVDs: 0.017% (SD: 0.18), respiratory: 0.041% (SD: 0.38) | - |
| 281 | Zhu et al., 2024 | China | 2013-2018 (6 years) | Time-stratified case-crossover, cox proportional regression | Total deaths: 6,685,146 | PM2.5, O3, NO2, SO2, CO, temperature, humidity PM2.5, O3, NO2, SO2, CO: No comparison, data per per 10ug/m3 increase; temperature, humidity: NA | All-cause-deaths, CVDs, respiratory diseases, pneumonia | Mortality | Air pollution: 0-3 days, others: 0-21 days | Joint exposures: all-cause: 16.65% (16.43, 16.87), CVD: 22.72% (22.41, 23.03), respiratory diseases: 24.82% (24.17, 25.47), pneumonia: 33.03% (31.80, 34.26), females: 21.32% (20.98, 21.66), males: 14.61% (14.32, 14.90); Single exposures: temperature: 11.16% (10.65, 11.67), relative humidity: 1.24% (1.03, 1.45), NO2: 4.08% (3.71, 4.45), O3: 2.84% (2.46, 3.22), PM2.5: 2.55% (2.30, 2.81), SO2: 4.49% (4.17, 4.80), CO: 0.85% (0.34, 1.37), | - |
| 282 | Zhai et al., 2023 | China | 2019-2023 (5 years) | Time-series, GLM, DLNM | Respiratory hospitalizations: 16,736 | TV: Interday 0-7d and Intraday 0-7d Comparison: SD of Tmin and Tmax (current day, previous 7 days) | Respiratory diseases | Morbidity (hospital admissions) | 0-7 days | Total: 0.013% (0.017, 0.394); Gender: female: 0.216% (0.074, 0.444), male: 0.209% (-0.058, 0.437); Age: 0-5y: 0.366% (0.220, 0.596), ≥65y: 0.081% (0.011, 0.156) | Total: 220 (-) |
| 283 | Tao et al., 2023 | China | 2011-2015 (5 years) | Time-series, Poisson Generalized Additive Model, DLNM | Outpatient cases for cardiovascular diseases: 729,409 | Hot Day Excess (HDE) and Hot Night Excess (HNE),  Comparison: population-weighted temperature thresholds | CVDs | Morbidity (outpatient visits) | 0-6 days | Total: hot day excess (HDE): 4.63% (2.44, 6.93), hot night excess (HNE): 15.70% (7.59, 25.01); Gender: male: HDE: 0.17%, HNE: 10.48%, female: HDE: 6.66%, HNE: 8.70%;  Age <45y: HDE: 0.01%, HNE: 17.70%, 46-65y: HDE: 0.05%, HNE: 8.00%, ≥66: HDE: 5.87%, HNE: 9.57% | Total: 38,111; Hot day excess: 5,542, hot night excess: 32,569 |
| 284 | Chen et al., 2024 | China | 2013-2017 (5 years) | Time-stratified case-crossover, conditional logistic regression | Hospital admissions for chronic kidney diseases: 3,490,416 | PM2.5 and PM10 exposure (single and cumulative lagged exposure at lag02 and lag03) Comparison: WHO guidelines (daily) | Chronic kidney disease | Morbidity (hospital admissions) | 0-4 days | Total: PM2.5: 2.83% (1.92, 3.74), PM10: 3.46% (2.33, 4.58) | - |
| 285 | Zhou et al., 2024 | China | 2018-2022 (5 years) | Time-stratified case-crossover, quasi-Poisson regression | Asthma hospitalizations: 54,134 | Sand-Dust Storms (SDS) - related PM10 Comparison: difference between mean PM10 of day and background value > 150 μg/m3 | Asthma | Morbidity (hospital admissions) | 0-2 days | Total: 1.64% (1.06, 2.18); Gender: male: 0.64% (-), female: 1.02% (-); Age: school-aged: 2.66% (-), adults: 1.03% (-) | Total: 885 (575, 1180) |
| 286 | Huang et al., 2024 | Multi country (Canada, South Korea, New Zealand, Taiwan, Thailand, Viet Nam) | 2000-2019 (20 years) | Time-series, quasi-Poisson regression, DLNM | Infectious disease hospitalizations: 2,2 00,000 | Tropical cyclones (days ≥ 34 knots) Comparison: NA | All infectious diseases, intestinal infectious diseases, sepsis, dengue | Morbidity (hospital admissions) | 0-60 days | Total: 0.72% (0.40, 1.01), intestinal: 0.33% (0.15, 0.49), sepsis: 1.31% (0.57, 1.95), dengue: 0.63% (0.10, 1.04) | - |
| 287 | Wang et al., 2024 | China | 2019-2021 (3 years) | Time-series, quasi-Poisson regression | Clinic visits due to upper respiratory tract infections: 934,180 | PM2.5, PM10, SO2, NO2 Comparison: average pollutant concentrations | Upper respiratory tract infection (URTI) | Morbidity (cases) | 0–5 days | Total: PM2.5: 6.5% (5.5, 7.6), PM10: 4.5% (3.7, 5.2), SO2: 9.8% (3.8, 16.3), NO2: 9.8% (8.5, 11.1) | - |
| 288 | Chen et al., 2024 | Multi country (43 countries globally) | 2000-2016 (17 years) | Time-series, quasi-Poisson regression | - | Wildfire-related O3 Comparison: daily concentration of ambient O3 | All-cause, CVDs, respiratory | Mortality | 0-2 days | All-cause: 0.58% (0.31, 0.85), CVD: 0.41% (-0.15, 0.91%), respiratory: 0.86% (0.18, 1.51) | All-cause: 31,606 (17,038, 46,027), CVD: 5,249 (-1,244, 11,620), respiratory: 4,657 (999, 8,206) |
| 290 | Feng et al., 2024 | Multi country (Bangladesh, China, Australia) | 2011-2017 (7 years) | Time-stratified case-crossover, logistic regression, DLNM | - | Hot, cold nights: 95th, 5th percentiles of daily minimum temperature Comparison: MMT | Pneumonia | Morbidity (hospitalization) | - | Hot nights: Australia: 21.2% (11.8, 28.1), Bangladesh: 15.2% (4.1, 23.8), China: 2.7% (0.4, 4.7) | - |
| 291 | Zhang et al., 2024 | China | 2013-2020 (8 years) | Time-stratified case-crossover, conditional logistic regression, DLNM | Deaths due to Alzheimer's Disease and other dementias: 399,036 | Heatwaves Comparison: daily mean temperatures for consecutive days | Alzheimer’s Disease and other dementias | Mortality | 0-7 days | Mildest heatwave: 12.281% (10.555, 14.015), most severe heatwave: 31.460% (28.724, 34.124) | - |
| 292 | Zhang et al., 2024 | China | 2010-2015 (6 years) | Time-series, quasi-Poisson, GAM | Stroke hospital admissions: 179,963 | Compound Drought and Hot Events (CDHEs) Comparison: 75th percentile | Strokes | Morbidity (hospital admissions) | 0-3 days | Hot event and high-severity drought: 24.40% (1.86, 50.20) | - |
| 293 | Zhan et al., 2024 | China | 2015-2021 (7 years) | Time-stratified case-crossover, quasi-Poisson regression | Mental disorder deaths: 1,685, dementia deaths: 1,199 | PM1, PM1-2.5, PM2.5, PM10, PM2.5-10 Comparison: 13, 9, 19, 33, 12 μg/m3 | Mental disorder and dementia-related diseases | Mortality | 0-3 days | Total: PM1: 5.55% (-), PM1-2.5: 6.49% (-), PM2.5: 7.68% (-), PM10: 10.66% (-), PM2.5-10: 15.11% (-) | - |
| 294 | Guo et al., 2023 | China | 2014-2021 (6 years) | Time-series, GAM | Total deaths: 4,276,989 | PM1, PM2.5, PM10, O3 No comparison, data per per 10 µg/m³ increase | All-cause, CVDs, respiratory | Mortality | 0-1 days | PM1: All-cause: 1.37% (1.22, 1.52), CVDs: 1.44% (1.25, 1.63), respiratory: 1.63% (1.25, 2.01); PM2.5: All-cause: 0.60% (0.52, 0.67), CVDs: 0.63% (0.53, 0.72), respiratory: 0.70% (0.51, 0.89); PM10: All-cause: 0.36% (0.31, 0.41), CVDs: 0.37% (0.30, 0.43), respiratory: 0.50% (0.38, 0.62); O3: All-casue: 0.80% (0.73, 0.87), CVDs: 0.85% (0.75, 0.96), respiratory: 1.15% (0.94, 1.36) | - |
| 296 | Zhao et al., 2024 | China | 2014-2019 (6 years) | Time-series, DLNM, conditional quasi-Poisson regression | Injury hospitalizations: 174,713 | Heatwaves  Comparison: temperature threshold ≥3 days ≥90th percentile | Injury related | Morbidity (hospital admissions) | 0-7 days | Total: 6.54% (3.94, 9.01) | Total: 361,447/year |
| 297 | Xu et al., 2023 | China | 2015-2020 (6 years) | Time-stratified case-crossover, conditional logistic regression | Myocardial infarction deaths: 202,678 | Heatwaves, PM2.5 Comparison: heatwave: T > 90th, 92.5th, 95th, 97.5th percentile for at least 2, 3, 4 days Comparison: Heatwaves: non-heatwave days, no comparison for PM2.5, data per per 10 µg/m³ increase | Myocardial infarction | Mortality | 0-1 days | Total: heatwaves: 1.18%-1.74% (-), joint exposure (heatwaves + PM2.5): 2.8% (-) | - |
| 299 | Jiang et al., 2023 | China | 2017-2019 (3 years) | Time-stratified case-crossover, conditional Poisson regression | Type 2 Diabetes hospitalizations: 92,381 | PM2.5, PM10, NO2, SO2, CO Comparison: WHO air quality standard | Diabetes (type 2) | Morbidity (hospital admissions) | 0-6 days | Total: NO2: 3.39% (2.26, 4.54), PM10: 0.33% (0.04, 0.62), PM2.5: 0.76% (0.35, 1.16), SO2: 12.68% (8.14, 17.42), CO: 79.00% (9.81, 129.18) | Total: NO2: 786 (-), PM10: 323 (-), CO: 2,127 (-) |
| 301 | Li et al., 2023 | China | 2016-2021 (6 years) | Time-series, Poisson regression, DLNM | Total deaths: 258,045 | Humidex (combined temperature and humidity index) Comparison: minimum mortality humidex (MMH) | CVDs | Mortality | 0-21 days | Total: 21.59% (18.12, 24.59), low humidex: 20.16% (16.72, 23.23) | - |
| 302 | Stafoggia et al., 2023 | Italy | 2016-2019 (chronic pollution) (4 years) 2003-2015 (heat exposure) (13 years) | Time-series, random forest (air pollution), DLNM (temperature) | - | Chronic PM2.5 and NO2 exposure, acute high summer temperatures Comparison: PM2.5: 10 μg/m3 and 5 μg/m3, NO2: 40 μg/m3 and 10 μg/m3, acute high summer temperatures: (75th to 99th percentile) | All-cause, CVDs, respiratory | Mortality | 0-21 days | PM2.5 threshold: 10 μg/m3: All-cause: 6.7% CVD: 2.2% Respiratory diseases: 0.4%  PM2.5 threshold: 5 μg/m3: All-cause: 11.7% CVD: 3.9% Respiratory diseases: 0.7%  NO2 threshold: 40 μg/m3: All-cause: 0.1% Respiratory diseases: 0.0%  NO2 threshold: 10 μg/m3: All-cause: 5.0% Respiratory diseases: 0.3%  Heat in 2015: All-cause: 2.3 (1.5-2.9) | PM2.5 threshold: 10 μg/m3: All-cause: 41,208 (22,083-59,330) CVD: 13,487 (11,233-16,725) Respiratory diseases: 2,662 (852-4,471)  PM2.5 threshold: 5 μg/m3: All-cause: 72,083 (38,974-102,888) CVD: 24,038 (20,079-29,680) Respiratory diseases: 4,638 (1,502-7,701)  NO2 threshold: 40 μg/m3: All-cause: 642 (376-914) Respiratory diseases: 37 (13-61)  NO2 threshold: 10 μg/m3: All-cause: 30,661 (18,187-43,109) Respiratory diseases: 1,587 (544-2,572)  Heat in 2015: All-cause: 14,521 (9,870-18,975) |
| 303 | Zhang et al., 2023 | China | 2016-2019 (4 years) | Time-series, quasi-Poisson regression, DLNM | Total influenza cases: 1,042,308 | Air pollutants (PM2.5, PM10, SO2, NO2, CO, O3) No comparison, data per per 10 µg/m³ increase | Influenza | Morbidity (incidence) | 0-14 days | Total: PM2.5: 4.46% (2.43, 6.43), PM10: 5.03% (2.33, 7.56), SO2: 5.36% (3.12, 7.58), NO2: 24.88% (18.02, 31.67), CO: 23.22% (17.56, 28.61) O3 during spring: 10.00% (4.76, 14.95) and summer: 3.65% (0.50, 6.59) | - |
| 307 | Boudreault et al., 2024 | Canada | 1996-2019 (24 years) | Time-series, DLNM, quasi-Poisson | - | Heat: T > MMT  Extreme heat: T > 95th percentile Comparison: MMT | All-cause | Mortality + Morbidity (various*) *hospital admissions, emergency departement visits, ambulacne transports, calls | 0-8 days | Heat: mortality: 2.1% (1.1, 3.1); Hospital admissions: 0.1% (0.1, 0.1), emergency departement visits: 2.3% (2.1, 2.6), ambulance transports: 2.7% (1.7, 3.6), calls: 2.3% (1.4, 3.2)  Extreme heat: mortality: 0.9% (0.6, 1.2); Hospital admissions: 0.1% (0.0, 0.1), emergency departement visits: 0.4% (0.4, 0.4), ambulance transports: 0.6% (0.5, 0.6), calls: 0.5% (0.4, 0.6) | Mortality: heat: 470, extreme heat: 200; |
| 310 | Mendrinos et al., 2024 | USA | 2015-2022 (8 years) | Time-series, Poisson regression, DLNM | Healthcare visits: 16,873,213 | Maximum daily heat index (HI), Comparison: 25.7°C | Non-specific | Morbidity (emergency departement visits) | 0-2 days | Total: 4.3% (4.2, 4.4); Rural: 3.7% (3.5, 3.9), non-rural: 3.1% (3.0, 3.2); Age: 0-64y: 4.2% (4.0, 4.3), >65y: 3.1% (2.9, 3.4) | - |
| 311 | Jiang et al., 2024 | China | 2014-2019 (6 years) | Time-series, Poisson regression, DLNM | Acute upper respiratory infections: 87,186 | Diurnal temperature range (DTR), Temperature variation (TV), Temperature change between neighboring days (TCN) Comparison: DTR, TV: 5% of the distribution, TCN: 0°C | Acute upper respiratory infections | Morbidity (outpatient hospital visits) | 0-7 days | Total: DTR: 24.26% (15.46, 32.05), TV0-1: 23.10% (15.59, 29.20), TV0-7: 19.24% (13.90, 24.63), TCN (below 0°C): 3.42% (1.60, 5.14) | - |
| 312 | Sharma et al.; 2024 | India | 2002-2018 (17 years) | Time-series, DLNM, Poisson regression | Total deaths: 684,142 | Maximum daily temperature (Tmax): T > 85th percentile Comparison: 26°C | All-cause | Mortality | 0-21 days | Total: Tmax: 3.58% (3.20, 3.96) | - |
| 313 | Qing et al., 2024 | China | 2016-2020 (5 years) | Time-series, GAM, DLNM | Respiratory diseases: 94,952 (outpatients), 72,410 (inpatients) | Moderate heat: 97.5th > Tapparent > 75th percentile extreme heat: > 97.5th percentile Comparison: optimum apparent temperature | Respiratory diseases | Morbidity (outpatient visits, HA) | 0-21 days | Moderate heat: outpatient: 6.28% (0.08, 10.48), inpatient: 4.17% (0.38, 7.88) Extreme heat: outpatient: 0.21% (1.06, 0.91), inpatient: 1.02% (0.39, 1.44) | - |
| 314 | Min et al., 2024 | Multi-country (Canada, Japan, Portugal, South Korea, Taiwan, UK) | 1987-2018 (32 years) | Time-series, quasi-Poisson regression | Acute kidney injury-related deaths: 41,379 | Air pollution (PM2.5, O3, NO2), Comparison: WHO guideline | Acute kidney injury (AKI) | Mortality | 0-28 days | Total: PM2.5: 1.9% (-), O3 (warm season): 6.3% (-), NO2: 5.2% (-) | - |
| 315 | Thawonmas et al., 2024 | Japan | 1973-2015 (42 years) | Time-stratified case-crossover, DLNM | Suicide cases: 1,049,592 | Moderate heat: 90th > T > 50th percentile Comparison: Minimum suicide temperature (23.1°C) / MMT | Suicide | Mortality | 0-3 days | Moderate heat: 9.9% (9.4, 10.4) | - |
| 316 | Tao et al., 2024 | China | 2011-2015 (5 years) | Time-series, DLNM | Outpatient visits due to mental disorders: 1,200,000 | Hot night excess (HNE), Hot day excess (HDE) Comparison: HNE: Tmin95th - HDE: Tmax95th | Mental disorders | Morbidity (outpatient visits) | 0-6 days | Hot night: 16.91% (10.11, 24.56), hot day: 7.24% (4.99, 9.59) | - |
| 317 | Tang et al., 2024 | China | 2013-2018 (6 years) | Time-stratified case-crossover, Difference-in-Differences (DID) | Respiratory disease deaths: 1,034,226 | Maximum Daily 8-hour Average Ozone (MDA8 O3) No comparison, data per per 10 µg/m³ increase | Respiratory | Mortality | Short-term: 0-3 days, long-term: annual exposure | Short-term: 3.00% (2.03, 3.95), long-term: 29.45% (26.86, 31.95) | Short-term: 30,790 deaths/year, long-term: 302,254 deaths over 6 years. |
| 319 | Wen et al., 2024 | Thailand | 2013-2019 (7 years) | Time-series, DLNM, Poisson regression | Outpatient admissions: 878,513,460, inpatient admissions: 32,616,600 | Moderate heat: 97.5th percentile > T > MMT  Extreme heat: T > 97.5th percentile Comparison: MMT | Non-cause, cause-specific | Morbidity (outpatient admissions) | 0-21 days | 1. Extreme heat (outpatient): 1.44% (1.36, 1.49), Extreme heat (inpatient): 0.96% (0.86, 1.02) 2. Moderate heat (outpatient): 6.71% (5.80, 7.41), Moderate heat (inpatient): 4.50% (3.62, 5.19) | - |
| 324 | Dimitrova et al., 2024 | Multi country: (29 low- and middle-income countries) | 2001-2019 (Different periods) (19 years) | Time stratified case-crossover, logistic regression, DLNM | Neonatal deaths: 40,073 | Heat: T > MMT Comparison: MMT | Neonatal deaths | Mortality | 0-2 days | Total: Heat: 1.5% (0.2, 2.6); Climate change attributable neonatal deaths: 32% (country range: 19-79%) | - |
| 329 | Tobias et al., 2024 | Central and South America (13 countries) | 1997-2018 (Different periods) (22 years) | Time-series, GLM, DLNM | Total (calculated) deaths: 9,975,723 | Heat: T > MMT Comparison: MMT | All-cause mortality | Mortality | 0-21 days | Total calculated: 10.40%, SD: 0.4812 | - |
| 330 | Scovronick et al., 2024 | Multi-country (33 countries) | 1969-2018 (Different periods) (50 years) | Time-series, GLM, DLNM | Total deaths: 88,483,994 | Heat: T > MMT Comparison: MMT | All causes, CVDs, respiratory | Mortality | 0-21 days | Results presented for people > 30y. Highest results for each diease group: all-cause: >75y: 0.46% (0.43, 0.48), CVDs: >75y: 0.54% (0.51, 0.57), respiratory: 45-59y: 1.22% (0.88, 1.50), non-cardiorespiratory: 30-44y: 0.78% (0.50, 1.01) | - |
| 331 | Ascaso et al., 2024 | Spain | 2013-2018 (6 years) | Time-series, GLM | Hospitalizstions due to bacterial foodborne diseases: 5,091 | Heat: T > MMT (12°C / 34°C) per 1°C increase Comparison: 12°C (whole year), 34° (summer months) | Bacterial foodborne diseases | Morbidity (hospital admissions) | 0-3 days | Whole year: 1.84% (1.02, 2.66), summer months: 4.17% (0.75, 7.46) | - |
| 336 | Xia et al., 2024 | China | 2016-2021 (6 years) | Time-series, quasi-Poisson, DLNM | Total deaths: 751,930 | Heat: T > MMT Comparison: MMT | Non-accidental | Mortality | 0-25 days | Total: 1.06% (0.76, 1.33) | - |
| 337 | Niu et al., 2023 | China | 2013-2018 (6 years) | Time-series, GAM, DLNM | - | Air health index - mix of Heat: T > MMT, PM1, PM2.5, PM10 Comparison: Heat: MMT, Air pollution: 0 μg/m3 | All-cause | Mortality | - | Joint exposure: 5.31% (4.58, 5.91) | Joint exposure: 188,246 (162,396, 209,533) |
| 340 | Huang et al., 2023 | China | 2013-2018 (6 years) | Time-series, DLNM | - | Heatwave (2+ or days with temperature > 90th percentile) Comparison: Non-heatwave days | Drowning | Mortality | - | Total: 11.4% (10.0, 12.9) | - |
| 354 | Bai et al., 2024 | China | 2013-2017 (5 years) | Time-stratified case-crossover, conditional logistic regression | Schizophrenia admissions: 817,296 | PM2.5, PM10, NO2, SO2, CO (absolute and APIN) Comparison: WHO guideline APIN: Air pollution increases between neighboring days | Schizophrenia | Morbidity (hospital admissions) | 0-5 days | Total: PM2.5: 2.37% (0.88, 3.88), PM10: 2.95% (1.46, 4.47), NO2: 4.61% (2.93, 6.32), SO2: 2.16% (0.59, 3.76), CO: 2.02% (0.39, 3.68) | - |
| 359 | Xu et al., 2024 | China | 2019-2021 (3 years) | Time stratified case-crossover, conditional logistic regression | Outpatient visits due to anxiety disorders: 126,112 | O3, PM2.5 Comparison: WHO guidelines | Anxiety Disorders | Morbidity (outpatient visits) | 0-3 days | Total: PM2.5: 3.47% (2.76, 4.16), O3: 2.62% (1.49, 3.71), PM2.5 + O3: 3.18% (3.38, 3.97), O3 + PM2.5: 2.26% (0.60, 3.86) | Total: PM2.5: 4376 (3486, 5250), O3: 1111 (634, 1575), PM2.5 + O3: 4016 (3006, 5008), O3 + PM2.5: 959 (252, 1637) |
| 360 | Yang et al., 2024 | China | 2013-2017 (5 years) | Time-series, GAM, DLM | Total hospitalizations: 23,492 | 30 different definitions of heatwaves divided into Low intensity heatwave, middle intense heatwave, high intensity heatwave | Urolithiasis | Morbidity (hospital admissions) | 0-7 days | Low intensity heatwave: 6.22% (1.43, 10.17), middle intense heatwave: 3.34% (1.10, 7.80), high intensity heatwave: 0.71% (0.00, 2.25) | Low intensity heatwave: 435 (100, 711), middle intense heatwave: 233 (77, 545), high intensity heatwave: 49 (0, 157) |
| 361 | Yang et al., 2024 | China | 2013-2017 (5 years) | Time-series, GLM, DLNM | Total hospitalizations: 23,492 | Heat: Tmean > 50th percentile (warm season) Comparison: 50th percentile | Urolithiasis | Morbidity (hospital admissions) | 0-5 days | Total: 7.85% (3.64, 11.44) | Total: 425 (207, 632) |
| 362 | Chen et al., 2023 | China | 2015-2020 (6 years) | Time-series, GLM, DLNM | Total hospitalizations: 23,492 | Heat: Tmean > MMT (25.3) Comparison: MMT | Atopic dermatitis | Morbidity (outpatient visits) | 0-7 days | Total: 3.0% (0.5, 5.0) | Total: 322.1 (45.9, 538.7) |
| 365 | Yezli et al., 2023 | Saudi Arabia | 2006-2015 (10 years) | Time-series, GLM, DLNM | Total deaths: 37,178 | Heat: T > MMT  Comparison: MMT | Non-accidental mortality | Mortality | 0-25 days | Total: Heat: 5.6% (−3.8, 13.2) | - |
| 371 | Xue et al., 2024 | Global | 1998-2019 (22 years) | Case-control, cox regression | Survival status of: 1,612,565 children | Anomolous precipitation: Precipitation anomaly deviation form mean Comparison: Long-term mean precipitation | All-cause (children) | Mortality | - | Total: -0.39 % (-1.49, 0.73); Weather phenomena: El Niño: - 3.45% (-1.84, -5.09), La Niña 1: 5.14% (3.70, 6.62), La Niña 2: 0.78% (− 1.17, 2.40) | - |
| 376 | Fatima et al., 2025 | Australia | 2005-2018 (14 years) | Time-series, quasi Poisson regression | Occupational injuries: 953,741 | Heatwave: T (3d) > 95th percentile Comparison: NA | Occupational injuries | Morbidity (cases) | 7-10 days | Total: 0.41% (SD: 0.33); Regions: Adelaide: 0.16% (-0.48, 0.69), Brisbane: 0.25% (-0.62, 0.98), Melbourne: 0.35% (-0.37, 0.92), Sidney: 0.89% (-0.15, 1.52) | - |
| 377 | Li et al., 2024 | 30 European countries | 2014–2023 (10 years) | Time-series, quasi-Poisson regression, DLNM | Total deaths (approx.): 1,000,000 | Heatwave: T(7d) > 90th percentile Comparison: MMT | All-cause | Mortality | 7 days | Before COVID-19: 0.492% (0.488, 0.496); Since COVID-19: 1.276% (1.266, 1.285) | - |
| 380 | Wei et al., 20223 | China | 2016-2021 (6 years) | Time-series, GLM, DLNM | Hospitalizations due to myocardial infarction: 24,489 | O3 No comparison, data per per 10 µg/m³ increase | Myocardial infarction | Morbidity (hospital admissions) | 0-7 days | Total: 11.66% (7.66, 15.40) | Total: 2856 (1878, 3768) |
| 389 | Jingesi et al., 2024 | China | 2013-2019 (7 years) | Time-series, quasi-Poisson regression, DLNM | Emergency departement visists due to CVDs: 64,890 | Heat: UCTI (heat index) > OET Moderate heat: UCTI 97.5th - OET Extreme heat: UTCI > 97.5th percentile Comparison: OET  EAD: Emergency ambulance dispatches UTCI: Universal termal climate index  OET: Optimum equivalent temperature (Minimum risk EAD) | CVDs | Morbidity (emergency departement visits) | 0-21 days | Total: heat: 0.39% (− 1.11, 1.79), extreme heat: 0.10% (− 0.19, 0.39), moderate heat: 0.29% (− 0.89, 1.45) | Total: heat: 255 (−742, 1176), extreme heat: 68 (−128, 258), moderate heat: 187 (−580, 918) |
| 391 | Jiang et al., 2024 | China | 2013-2019 (7 years) | Time-series, quasi-Poisson regression, DLNM | Ostheoathritis outpatient visits: 18,351,795 | PM2.5, PM10, NO2, SO2, 8h-O3, CO Comparison: WHO 24h-guidline (15 μg/m3, 45 μg/ m3, 25 μg/m3, 40 μg/m3, 100 μg/m3, 4 mg/m3) | Osteoathritis | Morbidity (outpatient visits) | - | Total: PM2.5: 2.16% (1.45, 2.85), PM10: 0.58% (0.05, 1.09), NO2: 3.83% (2.88, 4.74), SO2: 0.97% (0.15, 1.75), O3: 2.83% (1.43, 4.17), CO: 5.11% (4.10, 6.07) | - |
| 403 | Psistaki et al., 2024 | Greece | Athens: 1999–2019 (21 years) Thessaloniki: 1999–2018 (20 years) Cyprus: 2004–2019 (16 years) | Time series, Poisson regression, DLNM | Deaths due to CVDs: 323,648 (Athens), 81,448 (Thessaloniki), 31,620 (Cyprus) | Heat: T > MMT Comparison: MMT | CVDs | Mortality | 0-21d | Total: 2.94% (SD: 0.94%); Athens: 3.73%, SD: +- 0.15, Thessaloniki: 2.59%, SD: +- 0.59, Cyprus: 2.19%, SD: +- 1.01 |  |
| 404 | Lin et al., 2024 | USA | Los Angeles County: 2015–2022 (8 years) Clark County: 2015–2022 (7.5 years) | Time-series, quasi-Poisson regression, DLNM | Deaths due to people experiencing homelessness: 8,648 | Heat: T > MMT Moderate heat: 90th percentile > T > MMT Extreme heat: T > 90th percentile Comparison: MMT | All-cause | Mortality | 7 days | Clark County: heat: 49.4% (28.1, 62.2), moderate heat: 31.5% (10.4, 44.7), extreme heat: 24.7% (18.6, 28.3); Los Angelos County: heat: 5.2% (−0.2, 10.1), moderate heat: 2.9% (−1.3, 7), extreme heat: 2.2% (0.8, 3.5) | - |
| 406 | Rhamati et al.,2024 | Iran | 2017-2022 (6 years) | Time-series, DLNM | - | PM2.5 (satellite-based) High: 90th percentile, very high: 90-95th percentile, extreme: > 95th percentile Comparison: WHO guideline | Respiratory diseases, CVDs | Morbidity (hospital admissions) | - | CVDs: high: -0.09% (-0.21, 0.01), very-high: -0.02% (-0.06, 0.01), extreme: 0% (-0.05, 0.04); Respiratory diseases: high: 0.11% (0.04, 0.18), very-high: 0.05% (0.02, 0.07), extreme: 0.02% (-0.03, 0.05) | - |
| 408 | Kang et al., 2024 | Multi country (47 low- and middle-income countries) | 2000-2017 (18 years) | Case-control, generalized mixed-effects model | - | Dust-related PM2.5, Comparison: life-course averaged exposure | Anemia in children <5 years | Morbidity (prevalence) | - | Year 2000: 16.12% (14.60, 17.50); Year 2017: 16.73% (15.19, 18.16) | - |
| 414 | Xu et al., 2024 | China | 2015-2017 (3 years) | Time-series, Poisson regression | - | PM1 No comparison, data per per 10 µg/m³ increase | Non-specific, COPD, respiratory infections | Morbidity (hospital admissions) | 0-2 days | Total: 0.11% (0.01, 0.22) | Total: 6644 (351, 12,917) |
| 426 | Li et al., 2023 | China | 2016-2021 (6 years) | Time-series, DLNM | Hospital admissions due to urolithiasis: 5,956 | NO2 No comparison, data per per 10 µg/m³ increase | Urolithiasis | Morbidity (hospital admissions) | 0-4 days | NO2: 5.75% (0.60, 10.63); Gender: males: 4.40% (-2.35, 10.63), females: 3.19% (0.10, 6.10); Age: <65y: 4.58% (-1.01, 9.83), ≥65y: 18.96% (5.12, 30.80); Warm season: 16.81% (3.75, 28.11) | NO2: 342.47 (35.74, 633.12); Gender: males: 173.23 (-92.52, 418.50), females: 64.41 (2.02, 123.16); Age: <65y: 242.14 (-53.40, 519.71), ≥65y: 126.84 (34.25, 206.05) |
| 431 | Wen et al., 2024 | Multi country (47 Countries) | 1972–2020 (varies by country) (49 years) | Time-series, quasi-Poisson regression, DLNM | Total deaths: 126,600,000 | TV: Interday 0-7d and Intraday 0-7d Comparison: Minimum level TV | All-cause, CVDs, respiratory | Mortality | 0-7 days | All-cause: inter-day TV0–7: 0.35% (0.29, 0.41), intra-day TV0–7: 1.45% (1.31, 1.60); CVD: inter-day TV0–7: 0.66% (0.56, 0.76), intra-day TV0–7: 1.57% (1.35, 1.79); Respiratory: inter-day TV0–7: 0.44% (0.28, 0.60), intra-day TV0–7: 1.57% (1.18, 1.96) | Total: inter-day TV0–7: 17,120 (14,170, 20,068), intra-day TV0–7: 76,598 (69,023, 84,160) |
| 432 | Wu et al., 2024 | China | 2016-2020 (5 years) | Time-stratified case-crossover, DLNM | Total suicides: 9,642 | Extreme heat: T > 95th percentile Comparison: Lowest temperature | Suicides | Mortality | 0-10 days | Total: 31.7% (18.0, 43.2) | Total: 38 (22, 52) |
| 434 | López-Bueno et al., 2024 | Spain | 2013-2018 (6 years) | Time-series, quasi Poisson regression | Alcoholic liver diseases: 5,563 | Heat: T > MMT Comparison: MMT | Alcoholic liver disease | Morbidity (hospital admissions) | - | Total: 6.72% (0.43, 12.61) | - |
| 444 | Janos et al., 2024 | Czech Republic | 1987–2019 (All-cause mortality) (33 years)  1994–2019 (Cardiovascular & Respiratory mortality) (26 years) | Time-series, quasi-Poisson regression, DLNM | Total deaths: 3,717,972 | Moderate heat: 97.5th > T > MMT Extreme heat: T > 97th Comparison: MMT | All-cause, CVDs, respiratory | Mortality | 0-21 days | Total: moderate heat: 0.14% (0.11, 0.16), extreme heat: 0.23% (0.18, 0.27); Males: moderate heat: 0.03% (0.01, 0.05), extreme heat: 0.09% (0.05, 0.13); Females: moderate heat: 0.28% (0.23, 0.32), extreme heat: 0.36% (0.30, 0.41); Eldery (>65y): moderate heat: 0.02% (0.01, 0.03), extreme heat: 0.07% (0.05, 0.10) | Total: moderate heat: 153 (123, 183), extreme heat: 253 (203, 304) |
| 469 | Wu et al., 2023 | China | 2018–2021 (4 years) | Time-series, DLNM, GLM | Total dermatomyositis outpatient visits: 4,028 | NO2, O3, PM10 Comparison: 50th percentile of air pollutants | Dermatomyositis | Morbidity (outpatient visits) | 0-7 days | Total: NO2: 2.4% (0.6, 3.8), O3 :− 1.5% (− 3.9, −0.2), PM10: 0.2% (− 1.1, 1.0) | Total for NO2: 96.5 (18.0, 151.0), for O3: − 61.5 (− 158.0, − 10.0), for PM10: 8.4 (− 45.0, 38.0) |
| 470 | Zhao et al., 2023 | China | 2014–2019 (6 years) | Time-series, DLNM | Total deaths: 288,551 | Heat: Tmean > MMT, moderate heat: 97.5th > Tmean > MMT, extreme heat: Tmean > 97.5th percentile Warm season only Comparison: MMT | All-cause, Respiratory, CVDs | Mortality | 0-3 days | Total: from 2.9% (0.0, 6.5) to 20.6% (10.7, 29.6) Calculated: Total: 9.86% (7.21) | - |
| 476 | Ji et al., 2024 | China | 2015–2019 (5 years) | Time-series, GAM | Acute aortic dissections: 2,111 | PM2.5, PM10, O3 Comparison: WHO guidelines, China air quality primary standard | Acute aortic dissection | Morbidity (hospital admissions) | 0-3 days | WHO reference: Total: 22.4% (17.2, 27.4), PM2.5: 13.0% (10.5, 15.4), PM10: 6.1% (4.7, 7.4), O3: 3.3% (1.9, 4.5);  China reference: Total: 11.7% (8.5, 14.5), PM2.5: 3.6% (2.8, 4.2), PM10 4.8% (3.7, 5.8), O3: 3.3% (1.9, 4.5) | WHO reference: Total: 474 (363, 578), PM2.5: 275 (221, 326), PM10: 129 (100, 156), O3: 70 (42, 96);  China reference: Total: 246 (181, 307), PM2.5: 75 (60, 89), PM10 101 (79, 122), O3: 70 (42, 96) |
| 477 | Li et al., 2024 | Hong Kong | 2017-2022 (6 years) | Time-series, DLNM | Upper gastrointestinal bleeding attendences: 31,577 | PM2.5, NO2, O3 (warm season only) Comparison: 50th percentile of air pollutants | Upper gastrointestinal bleeding | Morbidity (emergency departement visits) | 0-7 days | Total: PM2.5: 0.44% (0.02, 0.84); Gender: male: 0.50% (0.04, 0.94), female: 0.38% ( 0.07, 0.83); Age: ≥65y: 0.46% (0.03, 0.88), <65y 0.32% (0.36, 0.97); Other: warm season: -0.56% (-1.05, -0.09), before COVID‐19: 0.82% ( 0.16, 1.78), during COVID‐19: 0.60% (0.34, 1.86);   NO2: 1.25% (0.69, 1.78); Gender: male: 1.42% (0.83, 1.98), female: 1.06% (0.42, 1.64); Age: ≥65y: 1.77% (0.92, 2.54), <65y: 1.17% (0.59, 1.73); Other: warm season: -0.48% (-1.36, 0.33), before COVID‐19: 1.82% (0.90, 2.74), during COVID‐19: 0.66% (-0.87, 1.97);   O3: 0.11% (-0.89, 2.64); Gender: male: 0.88% (-1.79, 2.94), female: 1.27% (-1.74, 3.51); Age: ≥65y: -0.19% (-3.21, 2.14), <65y: 2.35% (0.30, 4.32); Other: warm season: 0.05% (-3.55, 2.70), before COVID-19: 1.02% (-0.03, 1.16), during COVID-19: 0.42% (-1.15, 1.78) | Total: PM2.5: 1216 (55, 2327) Gender: male: 663 (52, 1240), female: 556 (-102, 1194) Age: ≥65y: 1105 (72, 2113), <65y: 112 (-127, 339) Other: warm season: -862 (-1282, -111), before COVID‐19: 756 (-112, 1674), during COVID‐19: 593 (-246, 1346)  NO2: 3453 (1903, 4907) Gender: male: 2059 (1194, 2865), female: 1389 (555, 2157) Age: ≥65y: 2831 (1430, 4170), <65y: 620 (322, 891) Other: warm season: -743 (-1666, 402), before COVID‐19: 3023 (1563, 4314), during COVID‐19: 477 (-629, 1425)  O3: 304 (-2468, 7298) Gender: male: 1153 (-2359, 3867), female: 1831 (-2512, 5071) Age: ≥65y: -467 (-7743, 5173), <65y: 590 (-105, 1516) Other: warm season: 77 (-4337, 3293), before COVID-19: 1466 (-108, 1862), during COVID-19: 304 (-832, 1288) |
| 486 | Borg et al., 2023 | Australia | 2005–2018 (14 years) | Time-series, DLNM | Occupational injuries: 1,208,004 | Heatwave: Tmean > 95th percentile for 3 days Comparison: NA | Occupational illnesses and injuries (OII) | Morbidity (cases) | 0-10 days | Total: 0.129% (0.107, 0.165); Gender: male: 0.135% (0.109, 0.176), female: 0.122% (0.053, 0.202); Age:15-29y: 0.165% (0.124, 0.222), 30-49y: 0.120 (0.075, 0.177), 50-75y: 0.113%(0.071, 0.166) | - |
| 487 | Hu et al., 2023 | China | 2007-2019 (13 years) | Time-series, DLNM | Myocardial infarction cases: 416,894 | Moderate heat: 97.5th > T > MMT, extreme heat: T > 97.5th percentile Comparison: MMT | Myocardial infarction (fatal, non-fatal) | Mortality + Morbidity (cases) | 0-31 days | Moderate heat: 0.9% (0.4, 1.3), non-fatal: -0.3% (-1.0, 0.4), fatal: 1.7% (1.2, 2.3); Extreme heat: 0.5% (0.4, 0.7), non-fatal: 0% (-0.3, 0.3), fatal: 0.9% (0.7, 1.1) |  |
| 489 | He et al., 2023 | China | 2015–2020 (6 years) | Time-series, DLNM | Total outpatient vistits: 35,700 | PM2.5, PM10, SO2, CO, O3 (warm season only) Comparison: Chinese National Ambient Air Quality Standards | Osteoarthritis | Morbidity (outpatient visits) | 0-7 days | Total: PM2.5: 2.33% (−0.94, 4.95), PM10: 2.70% (−2.86, 6.98), SO2: 1.38% (− 7.49, 7.50), CO: −1.23% (−4.3, 1.2), O3: −1.12% (−6.49, 3.22) | Total: PM2.5: 833 (−337, 1768), PM10: 963 (−1022, 2491), SO2: 493 (−2673, 2676), CO: −439 (−1534, 427), O3: −400 (−2317, 1149) |
| 498 | Ngyuen et al., 2024 | Vietnam | 2007-2019 (13 years) | Time-stratified case-crossover, conditional logistic regression | Hospital admissions (children): 302,345 | PM2.5, NO2 Comparison:  PM2.5: WHO (5 ug/m3), QCVN (25 ug/m3) NO2: WHO (10 ug/m3), QCVN (40 ug/m3) | Respiratory diseases (children) | Morbidity (hospital admissions) | - | PM2.5: WHO-guideline: 4.3% (3.5, 5.2), QCVN-guideline: 2.3% (1.8, 2.7); NO2: WHO-guideline: 2.6% (2.0, 3.2), QCVN-guideline: 0.2% (0.2, 0.3) | Total: PM2.5: WHO-guideline: 1619 (1299, 1935), QCVN-guideline: 853 (684, 1020), NO2: WHO-guideline: 962 (742, 1181), QCVN-guideline: 78 (60, 96) |
| 505 | Psistaki et al., 2023 | Greece | 1999–2018 (20 years) | Time-series, quasi-Poisson regression, DLNM | Cardiorespiratory deaths: 72,123 | Moderate heat: 99th percentile > T > MMT  Extreme heat: T > 99th percentile Comparison: MMT | Cardiorespiratory | Mortality | 0-21 days | Total: extreme heat: 0.7% (0.52, 0.88), moderate heat: 4.96% (3.16, 6.66); Males: extreme heat: 0.69% (0.45, 0.95), moderate heat: 5.38% (2.52, 7.87); Females: extreme heat: 0.72% (0.46, 0.97), moderate heat: 4.60% (1.96, 6.71); Eldery (>65y): extreme heat: 0.78% (0.59, 0.96), moderate heat: 5.55% (3.66, 7.41) | - |
| 532 | Jiang et al., 2024 | China | 6 years (2013–2018) | Time-series, GLM, DLNM | Total drowning deaths: 19,668 | Temperature + humidity compound: dry-hot days, wet-hot days Comparison: wet-non-hot days | Drowning | Mortality | 0-2 days | Dry-hot days: 23.83% (21.67, 26.99); Wet-hot days: 11.32% (9.64, 13.48) | _ |

AF, attributable fraction. TS, time series. CC, case-crossover. GLM, general linear model. DLNM, distributed lag non-linear model. GAM, generalized additive model. MMT, minimum mortality/morbidity temperature. Y, yes. N, no. CVD, cardiovascular diseases. HA, hospital admission. EDV, emergency department visit. CBI, cerebral infarction. ICH, intracerebral hemorrhage. URI, upper respiratory infections. DM, diabetes mellitus. PCDI, per capita disposable income. IRSAD, index of relative socio-economic advantage and disadvantage. OV, outpatient visits. EAD, emergency ambulance dispatches. COPD, chronic obstructive pulmonary disease. DTR, diurnal temperature range. TV. Temperature variability. TCN, temperature change between neighboring days. WHO, world health organization. d, day. DECH, daily excessive hours. AECOPD, acute exacerbation of COPD.

## S7. Excluded studies

| Study ID | Exclusion reason | Authors, Year | Country | Period of study | Start data | End date | Climate variable |
| --- | --- | --- | --- | --- | --- | --- | --- |
| 1 | Wrong outcome | Abdolahnejad et. al., 2018 | Iran (1 city) | 2 years | 2013 | 2014 | NO2, SO2, O3 |
| 7 | Quality assessment | Al-Hemoud et. al., 2019 | Kuwait | 4 years | 2014 | 2017 | PM2.5 |
| 11 | No total population data | Armstrong et al., 2017 | 11 countries | 10-40 years | 1972 | 2012 | Heat |
| 12 | Methodology not clear | Arranz et al., 2014 | Spain (1 city) | 10 years | 1998 | 2008 | PM2.5, PM10, O3 |
| 13 | Wrong study design | Arregodéc et al., 2023 | Colombia (1 Region) | 9 years | 2011 | 2019 | PM2.5, PM10 |
| 14 | No confidence interval | Åström et al., 2018 | Sweden (1 county) | 112 years | 1901 | 2013 | Heat |
| 15 | Wrong measure (Population attributable fraction) | Asl et. al., 2017 | Iran (1 city) | 1 year | Mar 14 | Mar 15 | PM10, PM2.5, NO2, SO2, O3 |
| 20 | Quality assessment | Bodor et. al., 2022 | Romania | 10 years | 2009 | 2018 | PM2.5, PM10 |
| 21 | Wrong measure (Population attributable fraction) | Bonyadi et al., 2016 | Iran (1 City) | 1 year | Dez. 12 | Dez. 13 | PM2.5 |
| 23 | Methodology not clear | Bouchriti et. al., 2023 | Marocco (1 city) | 1 year | 2016 | 2016 | O3 |
| 32 | No confidence interval | Cheng et al., 2020 | Vietnam (Hanoi) | 9 years | 2008 | 2016 | Heatwave |
| 34 | No confidence interval | Cheng et al., 2018 | Australia ( 5 cities) | 10 years | 2000 | 2009 | Warm season |
| 35 | No confidence interval | Cheng et al., 2018 | China and Australia (12 cities) | 6 years | 2010 | 2015 | Warm season |
| 38 | Quality assessment | Cui et el., 2016 | China (1 city) | 4 years | 2011 | 2014 | Heat |
| 41 | No confidence interval | Dąbrowiecki et al., 2023 | Poland | 8 years | 2011 | 2018 | PM10, PM2.5, NO2, SO2 |
| 43 | Data from a third article | De Marco et al., 2018 | Italy (1 City) | 2 years | 2015 | 2016 | PM2.5 |
| 45 | No total population data | de'Donato et al., 2018 | Italy (23 cities) | 18 years | 1999 | 2016 | Extreme heat |
| 49 | No total population data | Díaz et al., 2006 | Spain (Madrid) | 12 years | 1986 | 1997 | Maximal temperature |
| 51 | Wrong outcome | Doherty et al., 2009 | UK | 3 years | 2003 (without 2004) | 2006 | Heat, O3 |
| 57 | Data from a third article | Fenech et. Aquilina, 2019 | Malta (3 cities) | 6 years | 2010 | 2015 | PM2.5, NO2 |
| 58 | Data not in usable format | Fenech et. al., 2020 | Malta (1 city) | 8 years / 10,5 months | 2008 / 2020 | 2017 / Okt. 20 | NO2, O3 |
| 60 | Wrong outcome | Folkerts et al., 2020 | Netherlands | 18 years | 1995 | 2017 | Heat |
| 64 | Wrong measure (Population attributable fraction) | Goudarzi et. al., 2015 | Iran (1 city) | 1 year | 2009 | 2009 | PM10 |
| 65 | Projections only | Gu et al., 2020 | China (1 City) | 10 years | 2009 | 2018 | Heat |
| 66 | Wrong study design | Guo et al., 2016 | China | 9 years | 2001 | 2009 | PM2.5 |
| 68 | Methodology not clear | Hadei et al., 2017 | Iran (10 Cities) | 4 years | 2013 | 2016 | PM2.5 |
| 69 | Wrong measure (Population attributable fraction) | Hajizadeh et. al., 2020 | Iran (1 city) | 6 years | 2014 | 2019 | PM2.5 |
| 70 | Methodology not clear | Haley et al., 2009 | USA (1 State) | 5 years | 2001 | 2005 | PM2.5 |
| 76 | Data from a third article | Ho et. al., 2022 | Taiwan (1 city) | 7 years | 2013 | 2019 | PM2.5 |
| 78 | Projections only | Hu et al., 2019 | China (1 Province) | 7 years | 2009 | 2015 | Heat |
| 85 | Methodology not clear | Isaifan et. al., 2023 | 16 countries | 1 year | 2012 | 2012 | PM2.5 |
| 86 | No confidence interval | Jaffe et. al., 2003 | USA (3 cities) | 6 years | 1991 | 1996 | NO2, O3, PM10, SO2 |
| 91 | Data from a third article | Kaiser et al., 2004 | USA | 3 years | 1995 | 1997 | PM10 |
| 92 | Quality assessment | Kephart et al., 2022 | 6 Countries | 8-14 years | 2008 | 2015 | Heat |
| 93 | Wrong measure (Population attributable fraction) | Kermani et al., 2018 | Iran (1 City) | 10 years | 2005 | 2014 | O3, NO2, SO2, PM10, PM2.5 |
| 95 | No confidence interval | Kim et. al., 2014 | South Korea (Seoul) | 18 years (only May to Sep.) | 1992 | 2009 | Heat |
| 100 | No total population data | Lee et. al., 2021 | Switzerland and South Korea | 21 years (only May to Sep.) | 1995 | 2015 | Heat |
| 106 | Wrong measure (Population attributable fraction) | x | Iran (1 City) | 1.5 years | Nov. 17 | Mär. 19 | PM10 |
| 109 | Methodology not clear | Li et al., 2016 | China (Guangzhou) | 5 years | 2007 | 2011 | PM10, SO2, NO2 |
| 110 | Data from a third article | Li et al., 2010 | United Arab Emirates | 1 year | 2017 | 2017 | PM2.5, P10, O3 |
| 121 | Wrong measure (Population attributable fraction) | Liu et. al., 2021 | China (4 provinces) | 4 years | 2014 | 2017 | PM2.5 |
| 123 | No total population data | López-Bueno et al., 2019 | Spain (Madrid) | 10 years | 2000 | 2009 | Heatwave |
| 124 | No total population data | López-Bueno et al., 2020 | Spain (Madrid) | 4 years | 2010 | 2013 | Heatwave |
| 131 | No confidence interval | Luque Fernández et. al., 2009 | Zambia (Lusaka) | 4 years | 2003 | 2006 | Heat |
| 133 | Wrong measure (Population attributable fraction) | Luo et. al., 2020 | China (3 plateaus) | 4 years | 2015 | 2018 | PM10, PM2.5, SO2, O3, NO2, CO, |
| 135 | Data not in usable format | Ma et. al., 2021 | USA | 9 months | Mar 20 | Dec 20 | Temperature deviation |
| 139 | Wrong exposure | Ma et al., 2020 | China (1 Province) | 4 years | 2014 | 2017 | Heat |
| 140 | Wrong measure (Population attributable fraction) | Mai et al., 2021 | China | - | 2011 | 2015 | PM2.5 |
| 142 | Wrong measure (Population attributable fraction) | Malakootian et. al., 2020 | Iran (1 city) | 2 years | 2016 | 2017 | PM2.5, NO2, O3 |
| 149 | Wrong measure (Population attributable fraction) | Marzouni et al., 2017 | Iran | 1 years | 2012 | 2012 | PM10 |
| 151 | No total population data | Maté et. al., 2010 | Spain (1 city) | 3 years | 2003 | 2005 | PM2.5 |
| 158 | No confidence interval | Näyhä et. al., 2007 | Finnland | 6 years | Jan. 00 | Dez. 05 | Heat |
| 162 | Wrong outcome | Ortiz et al., 2017 | Spain | 10 years | 2000 | 2009 | PM10, PM2.5 |
| 165 | Methodology not clear | Parliari et. al., 2022 | Greece (Thessaloniki) | 11 years | 2006 | 2016 | Heat |
| 168 | Methodology not clear | Pascal et. al., 2018 | France | 11 years | 2000 | 2010 | Heat |
| 170 | Data not in usable format | Pozzer et. al., 2018 | Italy (1 city) | 14 years | 2000 | 2005 | PM2.5, PM10 |
| 176 | Data not in usable format | Ruiz-Páez et. al., 2023 | Spain (1 city) | 6 years | 2013 | 2018 | Heawave |
| 180 | Methodology not clear | Salvador et al., 2019 | Spain (4 Provinces) | 10 years | 2000 | 2009 | O3, NO2, PM10 |
| 184 | No confidence interval | Scovronick et Armstrong, 2012 | South Africa (1 city) | 20 years | 1996 | 2015 | Heat |
| 187 | Quality assessment | Shartova et. al., 2019 | Russia (1 city) | 18 years | 1999 | 2016 | Heat |
| 188 | Missing data | Shrikhande et. al., 2023 | India (4 districts) | 10 years | 2011 | 2020 | Heat |
| 190 | Methodology not clear | Stafoggia et al., 2009 | Italy (Rome) | 19 years | 1987 | 2005 | Summer |
| 191 | Quality assessment | Su et al., 2019 | China | 4 years | 2014 | 2017 | Heat |
| 194 | Methodology not clear | Sun et al., 2019 | China (Hong Kong) | 17 years | 2000 | 2016 | Heat |
| 198 | Wrong exposure | Tian et. al., 2016 | Hong Kong | 8 years | Jan. 05 | Dez. 12 | Cold |
| 202 | Methodology not clear | Trasande et al., 2016 | USA | 1 year | 2010 | 2010 | PM2.5 |
| 203 | Wrong measure (Population attributable fraction) | Uccelli et. al., 2ß16 | Italy | 5 years | 2008 | 2012 | PM10 |
| 210 | No confidence interval | Wang et. al., 2021 | China (270 cities) | 3 years | 2014 | 2016 | Heat |
| 211 | Wrong outcome | Wang et. al., 2020 | China | 3 years | 2000 / 2013 | 2004 / 2015 | PM2.5 |
| 215 | Methodology not clear | Wang et. al., 2020 | China (18 sites) | 4 years (only Jun. to Aug.) | 2014 | 2017 | Heat |
| 220 | No total population data | Wu et. al., 2019 | China (1 city) | 11 years | 2006 | 2020 | O3 and NO2 |
| 223 | No confidence interval | Wu et. al., 2022 | China (Beijing) | 3 years | 2013 | 2015 | PM2.5 |
| 225 | Data not in usable format | Xi et. al., 2020 | US | 5 years | 2008 | 2013 | Wildfire specific PM2.5 |
| 226 | Methodology not clear | Xu et. al., 2022 | China | 4 years | 2016 | 2019 | PM2.5, PM10, SO2, CO, NO2, O3 |
| 232 | Data from a third article | Yang et. al., 2022 | Global | 1 year | 2019 | 2019 | PM2.5 |
| 236 | No total population data | Yang et. al., 2020 | China (16 Cities) | 7 years | 2007 | 2013 | PM10 |
| 247 | Wrong exposure | Zhang et. al., 2022 | China (30 Cities) | 4 years | 2016 | 2019 | Heat |
| 251 | No total population data | Zhang et. al., 2018 | US (106 communities) | 14 years | 1987 | 2000 | Heat |
| 257 | Data not in usable format | Zheng et. al., 2022 | China (8 cities of Jiangsu province) | 5 years | 2015 | 2019 | Heat |
| 264 | Wrong exposure | Achebak et al., 2023 | Spain (Madrid and Barcelona) | 14 years | 2006 | 2019 | Non-optimal temperature (> or <MMT) during summer |
| 265 | Wrong exposure | Xu et al., 2023 | China | 18 years | 2011 | 2018 | Cooking fuel-related polution |
| 267 | Wrong metric (excess deaths) | Liu et al., 2023 | China | 3 years | 2013 | 2015 | TV |
| 271 | Wrong metric (Population attributable fraction) | Cleland et al., 2023 | USA (120 metropolitan areas) | 18 years | 2000 | 2017 | Heat and urban heat |
| 272 | Wrong metric (Population attributable fraction) | Thilakaratne et al., 2022 | USA (California) | 9 years | 2008 | 2016 | PM2.5 |
| 279 | Preprint | Harris et al., 2024 | Peru | 1 extreme precipitation event in 2023 | _ | _ | Extreme precipitation |
| 539 | Wrong metric (Population attributable fraction) | Shrestha et al., 2024 | Canada | 21 years | 2001 | 2021 | Heat |
| 289 | Preprint | Fernandez et al., 2024 | Multi-country | 23 years | 1996 | 2018 | Temperature |
| 295 | Wrong study design | Zhou, L et al.; 2024 | China | 20 years | 2000 | 2019 | Heat |
| 298 | Wrong metric (Population attributable fraction) | Liu, C et al.; 2023 | 10 countries | 27 years | 1994 | 2020 | Air pollution (PM and ozone) |
| 306 | Wrong study design | Ai et al.; 2024 | China | 10 years | 2000 | 2010 | Temperature |
| 309 | Wrong metric (Excess deaths) | Sun et al; 2024 | China | 9 years | 2013 | 2021 | Air pollution (O3) |
| 320 | Wrong metric (Population attributable fraction) | Wang et al.; 2024 | China | 7 years | 2013 | 2019 | Heatwave, hot nights |
| 321 | Wrong metric (Population attributable fraction) | Arsenovic et al., 2023 | Serbia | 15 years | 2001 | 2015 | Heat |
| 322 | Wrong metric (Population attributable fraction) | Rau et al., 2024 | USA | 6 years | 2016 | 2021 | Heatwave |
| 325 | Wrong metric (Excess deaths) | Huang et al., 2023 | Multi-country (14 countries) | 40 years | 1980 | 2019 | Tropical cyclones |
| 326 | Wrong metric (Excess deaths) | Quick, M, 2024 | Canada | 21 years | 2000 | 2020 | Heat |
| 327 | Wrong metric (No attributable fraction) | Picciotto et al., 2024 | USA (California) | 12 years | 2007 | 2018 | Wildfire-related PM2.5 |
| 328 | Third study data | Tsai et al., 2023 | Taiwan | 15 years | 2006 | 2020 | PM2.5 |
| 332 | Wrong exposure | Ji et al.; 2024 | China | 6 years | 2015 | 2020 | Humidity |
| 334 | Wrong metric (No attributable fraction) | Qian et al.; 2024 | China | 17 years | 2005 | 2021 | Air pollution (PM) |
| 335 | Wrong language | Wang, D et al; 2024 | China | 8 years | 2013 | 2020 | Air pollution (PM and ozone) |
| 338 | Wrong metric (Population attributable fraction) | Chatterjee, D et al.; 2023 | India | 1 year | 2019 | 2019 | Air pollution (PM) |
| 339 | Wrong language | Di Blasi, C et al.; 2023 | Italy | 10 years | 2006 | 2015 | Heat |
| 341 | Wrong metric (Population attributable fraction) | Li et al.; 2023 | China | 7 years | 2013 | 2019 | Air pollution (PM, O3) |
| 341 | Wrong metric (Population attributable fraction) | Li, J et al.; 2023 | China | 7 years | 2013 | 2019 | Air pollution (PM and ozone) |
| 342 | Wrong metric (No attributable fraction) | Madani, N.A et al.; 2023 | United States | 9 years | 2010 | 2018 | Air pollution (VOC) |
| 343 | Wrong metric (No attributable fraction) | Hajat, S et al.; 2023 | United Kingdom | 9 years | 2011 | 2010 | Temperature |
| 344 | Quality assessment | Han et al., 2024 | China (Zibo City) | 5 years | 2015 | 2019 | Daily concentrations of O3, PM10, and PM2.5 in comparison to the WHO’s 2021 standards |
| 345 | Wrong outcome | Pan et al., 2023 | Global | 30 years | 1990 | 2019 | Air pollution |
| 346 | Third study data | Yu et al., 2024 | Global | 30 years | 1990 | 2019 | PM2.5 |
| 347 | Wrong metric (No attributable fraction) | Wang et al.; 2023 | China | 15 years | 2004 | 2018 | Humidity, rainfall, air pollution (PM) |
| 350 | Wrong metric (No attributable fraction) | Zhao et al., 2023 | China | 2 years | 1999 | 2020 | Air pollution (O3) |
| 351 | Wrong metric (No attributable fraction) | Achebak et al.; 2024 | Spain | 16 years | 2004 | 2019 | Temperature and humidity |
| 352 | Wrong metric (No attributable fraction) | Amubieya et al.; 2024 | United Stated | 12 years | 2005 | 2016 | Air pollution (PM) |
| 353 | Wrong metric (No attributable fraction) | Alahmad et al.; 2024 | Kuwait | 3 years | 2017 | 2019 | Heat and dust |
| 355 | Wrong metric (No attributable fraction) | Borroni et al.; 2024 | Italy | 3 years | 2020 | 2022 | Air pollution (PM) and temperature |
| 356 | Third study data | Tunesi et al., 2024 | Italy (Milan) | 1 year | 2019 | 2019 | Annual levels of NO2, PM10, PM2.5 above the WHO's guidelines |
| 356 | Wrong study design | Tunesi et al.; 2024 | Italy | 1 year | 2019 | 2019 | Air pollution (PM, NO2) |
| 357 | Quality assessment | Wang et al., 2023 | China (Sichuan Basin) | 5 years | 2015 | 2019 | Heat |
| 358 | Projections only | Yang et al., 2024 | Taiwan | 13 years | 2011 | 2023 | Heat |
| 363 | Wrong metric (Population attributable fraction) | Yin et al., 2024 | China (306 cities) | 29 years | 1986 | 2014 | Heat |
| 364 | Wrong metric (No attributable fraction) | Hundessa et al., 2024 | Global | 20 years | 2000 | 2019 | Heat |
| 366 | Wrong metric (No attributable fraction) | Song et al.; 2025 | China | 4 years | 2017 | 2020 | Air pollution (PM) |
| 366 | Wrong metric (Population attributable fraction) | Song and Hao, 2023 | China (11 cities on the Fenwei Plain) | 7 years | 2014 | 2020 | O3 |
| 367 | Wrong metric (No attributable fraction) | Zhu et al., 2023 | sub-Saharan Africa | 30 years | 1985 | 2014 | Heat |
| 368 | Wrong metric (No attributable fraction) | Chen et al., 2024 | China | 6 years | 2015 | 2020 | Long term exposure to PM1 |
| 370 | Preprint | Geldsetzer et al.; 2024 | US | 27 years | 1990 | 2016 | Air pollution (PM) |
| 372 | Wrong metric (No attributable fraction) | Yang et al.; 2023 | China | 3 years | 2014 | 2016 | Air pollution (PM) |
| 373 | Wrong metric (Population attributable fraction) | Liao et al; 2023 | China | 1 year | 2019 | 2019 | Air pollution (PM) |
| 374 | No access | Liu et al.; 2023 | China | - | - | - | Air pollution (various) |
| 378 | No access | Zhang et al.; 2024 | China | - | - | - | Air pollution (PM) |
| 381 | Wrong outcome | Zorzenao et al., 2025 | Brazil | 1.5 years | Mar 2021 | Oct 2022 | Air pollution (PM) |
| 382 | Wrong metric (No attributable fraction) | Hu et al.; 2024 | Mexico | - | - | - | Air pollution, temperature |
| 383 | Wrong exposure | Kashyap et al; 2024 | India | 1 year | - | - | - |
| 384 | No access | Ma et al.; 2024 | China | 5 years | 2013 | 2017 | Air pollution (NO2) |
| 385 | Wrong study design | Zhu et al.; 2024 | China | 16 years | 2006 | 2021 | Heatwave |
| 387 | Wrong metric (Population attributable fraction) | Bryan et al.; 2023 | US | 1 year | 2016 | 2016 | Air pollution (PM) |
| 388 | Wrong metric (No attributable fraction) | Yang et al.; 2024 | Taiwan | 10 years | 2012 | 2021 | Air pollution (PM) |
| 390 | No access | Yang et al.; 2024 | China | - | - | - | Heat, Heatwave |
| 390 | Quality assessment | Alwadi et al.; 2024 | Jordan | 19 years | 2000 | 2018 | Heat |
| 392 | Wrong metric (Population attributable fraction) | Maji et al.; 2024 | US | 1 year | 2022 | 2022 | Air pollution (PM, O3) |
| 393 | Wrong metric (No attributable fraction) | Liu et al.; 2024 | China | 7 years | 2014 | 2020 | Air pollution (PM) |
| 394 | Wrong metric (No attributable fraction) | Li et al.; 2024 | China | 7 years | 2013 | 2019 | Air pollution (various) |
| 395 | Wrong metric (Population attributable fraction) | Zhu et al.; 2024 | Iran | 6 years | 2013 | 2018 | Air pollution (O3) |
| 396 | Wrong metric (Population attributable fraction) | Jiang et al.; 2024 | China | 1 year | 2016 | 2016 | Air pollution, water pollution |
| 397 | Wrong exposure | Wu et al.; 2024 | China | 8 years | 2015 | 2022 | - |
| 398 | Wrong metric (Population attributable fraction) | Wang et al.; 2023 | China | 9 years | 2010 | 2018 | Air pollution (NO2) |
| 399 | Wrong metric (No attributable fraction) | Lee et al.; 2023 | US | 17 years | 2000 | 2017 | Air pollution (PM) |
| 400 | Wrong metric (Population attributable fraction) | Leili et al; 2023 | Iran | 1 year | 2018 | 2018 | Air pollution (PM) |
| 402 | Third study data | He et al.; 2024 | China | 12 years | 2006 | 2017 | Air pollution (PM) |
| 405 | Wrong metric (No attributable fraction) | Tajudin, M et al.; 2024 | Malaysia | 11 years | 2005 | 2015 | Air pollution (PM) |
| 407 | Wrong outcome | Chen et al.; 2024 | China | 7 years | 2014 | 2020 | Temperature |
| 409 | Wrong metric (Population attributable fraction) | de Bont, J et al.; 2024 | India | 12 years | 2008 | 2019 | Air pollution (PM) |
| 410 | Wrong study design | Min, Y et al.; 2024 | China | _ | 2011, 2013, 2015, 2018 | _ | Air pollution (PM, OM, NO3-, NH4+) |
| 411 | Wrong metric (Population attributable fraction) | Xu, C et al.; 2024 | China | 3 years | 2013 | 2015 | Air pollution (PM, O3) |
| 412 | Wrong metric (Population attributable fraction) | Sharma, A et al.; 2024 | Taiwan | 14 years | 2005 | 2018 | Air pollution (PM) |
| 413 | Wrong metric (Excess deaths) | Alahmad, B et al.; 2024 | Multi-country | 41 years | 1979 | 2019 | Temperature |
| 415 | Wrong metric (No attributable fraction) | López-Bueno, J; 2023 | Spain (Getafe) | 14 years 10 months | 01 January 1999 | 31 October 2013 | heat |
| 416 | Wrong outcome | Li, Y; 2023 | Australia (Adelaide) | 10 years | 01 January 2012 | 31 December 2021 | heat |
| 419 | Wrong metric (No attributable fraction) | Zhou et al.; 2023 | China | 7 years | 2013 | 2019 | Air pollution (PM) |
| 420 | Wrong metric (No attributable fraction) | Shen et al.; 2023 | China | 7 years | 2014 | 2020 | Air pollution (PM, NO2, SO2, CO, O3) |
| 421 | Wrong metric (No attributable fraction) | Mebrahtu et al; 2023 | United Kingdom | 4 years | 2018 | 2021 | Air pollution (PM and NO2) |
| 422 | Wrong metric (Population attributable fraction) | Wang et al.; 2023 | China | 5 years | 2013 | 2017 | Air pollution (PM, NO2, SO2) |
| 423 | Wrong metric (No attributable fraction) | Wang et al.; 2023 | China | 6 years | 2014 | 2019 | Air pollution (PM, ozone) |
| 425 | Wrong metric (Population attributable fraction) | Lin et al.; 2023 | China | 7 years | 2014 | 2019 | Air pollution (PM) |
| 427 | Wrong metric (No attributable fraction) | Osborne et al.; 2024 | Australia | 28 years | 1992 | 2019 | Heatwave |
| 428 | Wrong metric (Population attributable fraction) | Wang et al.; 2024 | China | 16 years | 2005 | 2018 | Air pollution (NO2) |
| 429 | Wrong metric (Population attributable fraction) | Zhu et al.; 2024 | China | 9 years | 2010 | 2018 | Air pollution (O3) |
| 430 | Duplication | Zhao et al.; 2024 | China | 5 years | 2015 | 2019 | Air pollution (PM, O3) |
| 430 | Wrong metric (Population attributable fraction) | Zhao et al.; 2024 | China, Hong Kong | 5 years | 2015 | 2019 | Air pollution (PM, O3) |
| 433 | Wrong metric (No attributable fraction) | Nawsherwan et al.; 2024 | Multi country (BRICS) | 30 years | 1990 | 2019 | Air pollution (PM) |
| 435 | Wrong metric (No attributable fraction) | Hang et al.; 2024 | China | 6 years | 2013 | 2018 | Heatwave, ozone |
| 437 | Wrong metric (No attributable fraction) | Cheng et al.; 2024 | China | 5 years | 2015 | 2019 | Temperature |
| 438 | Wrong metric (No attributable fraction) | Li et al.; 2024 | China | 6 years | 2015 | 2020 | Air pollution (PM) |
| 439 | Wrong metric (No attributable fraction) | Kang et al.; 2024 | South Korea | 10 years | 2011 | 2020 | Heatwave |
| 440 | Wrong metric (No attributable fraction) | Xu et al.; 2024 | China | 4 years | 2016 | 2019 | Air pollution (PM) |
| 441 | Wrong metric (Population attributable fraction) | Li, Y; 2023 | China (Jiangsu province) | 5 years | 2016 | 2020 | PM2.5 |
| 442 | Wrong metric (No attributable fraction) | Ma, Y; 2023 | United Kingdom | 15 years 2,5 months | 2006 | 16 March 2021 | PM2.5, PM10, NO2, NOX |
| 443 | Wrong metric (No attributable fraction) | Ma, Y; 2023 | China (Jilin province) | 2 years | 01 January 2015 | 31 December 2016 | PM2.5, O3 |
| 443 | Wrong metric (Population attributable fraction) | Ma et al.; 2023 | China | 2 years | 2015 | 2016 | Air pollution (PM , O3) |
| 445 | Wrong metric (Population attributable fraction) | Yang, B et al.; 2023 | China | 3 years | 2017 | 2019 | Air pollution (PM, O3, CO) |
| 446 | Wrong metric (Population attributable fraction) | Yuan, C et al.; 2023 | China | 16 years | 2000 | 2015 | Air pollution (PM) |
| 447 | Wrong metric (Population attributable fraction) | Zha, Q et al.; 2023 | China | 6 years | 2016 | 2021 | Temperature |
| 448 | Wrong metric (Population attributable fraction) | Zhang, F et al.; 2023 | China | 20 years | 2001 | 2020 | Air pollution (PM) |
| 449 | Wrong metric (Population attributable fraction) | Zhu, C et al.; 2023 | China | 7 years | 2014 | 2020 | Air pollution (PM and ozone) |
| 450 | Wrong metric (Population attributable fraction) | Bozzani, A et al.; 2024 | Italy | 7 years | 2012 | 2018 | Air pollution (PM) |
| 451 | Wrong metric (Population attributable fraction) | Chen, J et al.; 2024 | Denmark, England, Norway and Rome | 18 years | 2000 | 2017 | Air pollution (PM, O3, NO2) |
| 452 | Wrong metric (No attributable fraction) | Chen, L et al.; 2024 | China | 14 years | 2008 | 2021 | Air pollution (fine particulate matter) |
| 453 | Wrong metric (No attributable fraction) | Cromar, K et al.; 2024 | United States | 3 years | 2018 | 2020 | Air pollution (Wildfires) |
| 454 | Wrong metric (No attributable fraction) | Dos Santos, D.M et al.; 2024 | Brazil | 19 years | 2000 | 2018 | Heatwave |
| 455 | Wrong metric (Population attributable fraction) | Xue, Y et al.; 2023 | China | 1 year | 2015 | 2015 | Air pollution (PM) |
| 456 | Wrong metric (No attributable fraction) | de Bont, J et al.; 2024 | India | 13 years | 2008 | 2019 | Heatwave |
| 457 | Wrong metric (No attributable fraction) | Downward, G.S; 2024 | Bangladesh, India, Iran, Japan, South Korea, and Taiwan | 18 years | 1991 | 2008 | Air pollution (PM) |
| 458 | Wrong metric (No attributable fraction) | Zhu et al.; 2024 | China | 3 years | 2019 | 2021 | Heat |
| 459 | Wrong metric (No attributable fraction) | Zhuang et al.; 2024 | China | 6 years | 2013 | 2018 | Air pollution (PM) |
| 460 | Wrong metric (No attributable fraction) | Zhou et al.; 2024 | China | 3 years | 2010 | 2012 | Air pollution (PM) |
| 461 | Wrong metric (Population attributable fraction) | Zhou et al.; 2023 | China | 4 years | 2016 | 2019 | Air pollution (PM) |
| 462 | Wrong metric (No attributable fraction) | Zhou et al; 2024 | China | 7 years | 2013 | 2020 | Temperature |
| 463 | Wrong metric (No attributable fraction) | Zhao et al.; 2024 | China | 7 years | 2015 | 2021 | Temperature |
| 464 | Wrong metric (No attributable fraction) | Zhang et al.; 2024 | China | 3 years | 2017 | 2019 | Air pollution (PM, NO2, SO2, O3) |
| 465 | Wrong metric (No attributable fraction) | Zhang et al.; 2024 | United Kingdom | 5 years | 2006 | 2010 | Air pollution (PM and NO2) |
| 466 | Wrong metric (No attributable fraction) | Zhang et al.; 2024 | China | 3 years | 2016 | 2018 | Temperature and humidity |
| 467 | Wrong metric (No attributable fraction) | Zhang et al.; 2024 | United Kingdom | 6 years | 2006 | 2010 | Air pollution (PM, NO2, O3) |
| 468 | Wrong metric (No attributable fraction) | Zhang et al.; 2024 | United Kingdom | 5 years | 2006 | 2010 | Air pollution (ozone) |
| 471 | Data not in usable format | Dawson, L; 2024 | Australia (Victoria) | 7 years | 01 January 2014 | 31 December 2020 | PM2.5, CO, NO2, O3, SO2 |
| 472 | Wrong metric (No attributable fraction) | Fan, Y; 2024 | China (Anhui province) | 3 years | 2016 | 2018 | Heat, PM2.5, SO2, O3 |
| 473 | Wrong metric (No attributable fraction) | Fang, B; 2024 | China (Shanghai) | 18 years | 2003 | 2020 | PM2.5, PM10 |
| 474 | Wrong metric (No attributable fraction) | Fang, F; 2024 | USA (Los Angeles) | 5 years | 1999 | 2004 | PM2.5 |
| 475 | Wrong metric (No attributable fraction) | Feng, H; 2024 | China (Beijing) | 4 years | 2008 | 2011 | PM2.5 |
| 479 | Wrong metric (No attributable fraction) | Yang, X; 2024 | China (Sichuan province) | 3 years | 01 January 2017 | 31 December 2019 | PM2.5, PM10, NO2 |
| 480 | Wrong metric (No attributable fraction) | Young, R; 2024 | USA | 87 years | 1930 | 2015 | Tropical Cyclones |
| 481 | Dublication | Zhang et al., 2024 | Multi country (7 countries) | 20 years | 2000 | 2019 | Air pollution (wildfire PM) |
| 482 | Wrong metric (No attributable fraction) | Zhao, K; 2024 | China (Shandong province) | 7 years | 2013 | 2019 | PM2.5, O3 |
| 483 | Wrong metric (No attributable fraction) | Zhou, J; 2024 | China (Shanghai) | 10 years | 2008 | 2017 | PM2.5 |
| 484 | Wrong metric (No attributable fraction) | Lin, C; 2023 | China (Sichuan province) | 2 years | 01 January 2018 | 31 December 2019 | PM2.5, PM10, CO, NO2, SO2 |
| 485 | Projections only | Liu et al., 2023 | China | 1 year | 2000 | 2000 | Heatwave |
| 488 | Wrong metric (No attributable fraction) | Wang, Y; 2023 | USA (several states) | 15 years | 2002 | 2016 | PM2.5, NO2, O3 |
| 490 | Wrong metric (No attributable fraction) | Fu, Z et al.; 2023 | United Kingdom | 5 years | 2006 | 2010 | Air pollution (PM, O3) |
| 491 | Wrong metric (No attributable fraction) | Liu, T et al.; 2024 | China | 6 years | 2015 | 2020 | Temperature |
| 492 | Wrong metric (No attributable fraction) | Bui, L et al.; 2023 | China | 1 year | 2019 | 2019 | Air pollution (PM) |
| 493 | Wrong metric (No attributable fraction) | Chu, B et al.; 2023 | United States | 1 year | 2021 | 2021 | Temperature |
| 494 | Wrong metric (No attributable fraction) | Rerolle et al.; 2023 | Bangladesh | 30 years | 1988 | 2017 | Floods |
| 495 | Wrong metric (No attributable fraction) | Ilie et al.; 2023 | Romania | 3 years | 2020 | 2022 | Air pollution (PM, NO2, VOCs) |
| 496 | Wrong metric (Population attributable fraction) | Ren et al.; 2023 | China | 5 years | 2014 | 2018 | Heat |
| 497 | Wrong exposure | Beck et al.; 2024 | Multi country (Europe) | 1 year | 2022 | 2022 | - |
| 499 | No access | Maleki et al.; 2024 | Iran | - | - | - | Heat |
| 500 | Methods unclear | Li et al.; 2023 | China | - | - | - | Heat |
| 501 | Wrong exposure | Wagatsuma et al.; 2024 | Japan | 20 years | 2000 | 2019 | Temperature, humidity |
| 504 | Wrong metric (No attributable fraction) | Gong et al., 2024 | China | 8 years | 2013 | 2020 | O3 and heat |
| 506 | Duplication | Li et al., 2025 | Hong Kong | 6 years | 2017 | 2022 | Air pollution (PM, NO2, O3) |
| 507 | Foreign language | Perčič et al., 2024 | Romenia | 5 years | 2013 | 2017 | Heatwave |
| 508 | Wrong metric (No attributable fraction) | Guo et al., 2024 | Hong Kong | 20 years | 2000 | 2019 | Heat |
| 509 | Wrong metric (No attributable fraction) | Ham et a., 2024 | South Korea | 16 years | 2006 | 2021 | PM2.5 |
| 510 | Wrong metric (No attributable fraction) | Jiang et al., 2024 | China | 7 years | 2013 | 2019 | PM2.5 and PM2.5-10 |
| 511 | Wrong metric (No attributable fraction) | Lin et al., 2024 | USA | 14 years | 2005 | 2018 | Multiple air pollutants |
| 512 | Wrong metric (No attributable fraction) | Liu et al., 2024 | China | 7 years | 2013 | 2019 | O3 |
| 513 | Wrong metric (No attributable fraction) | Ma et al., 2024 | China | 5 years | 2015 | 2019 | PM and heat index |
| 514 | Wrong metric (No attributable fraction) | Mano et al., 2014 | Japan | 5 years | 2015 | 2019 | Heat |
| 514 | Wrong metric (No attributable fraction) | Min et al., 2024 | South Korea | 7 years | 2015 | 2021 | PM2.5 and O3 |
| 515 | Wrong metric (No attributable fraction) | Ni and Shi, 2023 | China | 6 years | 2014 | 2019 | PM |
| 516 | Wrong metric (No attributable fraction) | Niu et al., 2024 | China | 4 years | 2018 | 2021 | Multiple air pollutants |
| 517 | Wrong metric (No attributable fraction) | Pan et al., 2024 | China | 4 years | 2017 | 2020 | Heatwave and PM2.5 |
| 518 | Wrong metric (No attributable fraction) | Park et al., 2024 | South Korea | 14 years | 2006 | 2019 | Heat |
| 519 | Wrong metric (No attributable fraction) | Peng et al., 2024 | China | 1 year | 2019 | 2020 | O3 |
| 520 | Wrong metric (No attributable fraction) | Poobunjirdkul et al., 2024 | Thailand | 4 years | 2018 | 2021 | PM 2.5 |
| 521 | Wrong metric (No attributable fraction) | Shi et al., 2024 | China | _ | _ | _ | PM 2.5 |
| 522 | Wrong metric (No attributable fraction) | Tajudin et al., 2024 | Japan | 7 years | 2012 | 2018 | PM 2.5 and O3 |
| 523 | Wrong metric (No attributable fraction) | Tran et al., 2024 | Vietnam | 3 years | 2019 | 2021 | PM 2.5 |
| 524 | Wrong metric (No attributable fraction) | Martin et al., 2024 | Canada | 19 years | 2001 | 2019 | Heat |
| 525 | Wrong metric (No attributable fraction) | Uttajug et al., 2024 | Thailand | 4 years | 2018 | 2021 | Fire-related PM2.5 |
| 526 | Foreign language | Wang et al., 2024 | China | 5 years | 2014 | 2018 | PM 2.5 |
| 527 | Wrong metric (No attributable fraction) | Wang et al., 2024 | China | 7 years | 2009 | 2015 | PM |
| 528 | Wrong metric (No attributable fraction) | Wicki et al., 2024 | Switzerland | 14 years | 2003 | 2016 | Heat |
| 529 | Wrong metric (No attributable fraction) | Wu et al., 2024 | China | 3 years | 2017 | 2019 | Multiple air pollutants |
| 530 | Wrong metric (No attributable fraction) | Xu et al., 2024 | Estonia, Norway, Denmark, Sweden, Iceland | 14 years | 2010 | 2023 | Multiple air pollutants |
| 531 | No access | Shen et al.; 2024 | China | - | - | - | Air pollution (PM) |
| 533 | Wrong metric (No attributable fraction) | Hertzog et al., 2024 | Australia | 20 years | 2001 | 2019 | PM 2.5 |
| 534 | Wrong outcome | Liu et al., 2024 | China | 5 years | 2015 | 2019 | Multiple air pollutants |
| 536 | Wrong metric (Population attributable fraction) | Peng et al.; 2024 | China | 9 years | 2011 | 2019 | Air pollution (PM) |
| 537 | Wrong exposure | Yang et al.; 2023 | United States | 9 years | 2011 | 2019 | Temperature |
| 538 | Wrong study design | Chitu et al.; 2023 | Romania | 21 years | 1999 | 2019 | Temperature |
| 539 | No access | Tobias, A et al.; 2023 | Spain | 27 years | 1990 | 2016 | Temperature |
| 540 | No access | Ding et al; 2024 | China | 3 years | 2013 | 2015 | Temperature, air pollution (PM) |
| 541 | No access | Zhai et al.; 2024 | China | _ | _ | _ | Temperature, humidity and wind speed |
| 542 | No access | Zhai et al., 2024 | China | _ | _ | _ | _ |
| 543 | No access | Talukder et al., 2024 | Australia | 12 years | 2010 | 2021 | Heat |
| 544 | Duplicate (already included in the first round) | Crank et al., 2023 | USA | 9 years | 2006 | 2014 | Heat |
| 323 | No relevant data available | Zheng et al.; 2023 | China | 6 years | 2014 | 2019 | Temperature variability |
| 369 | No relevant data available | Geldsetzer et al.; 2024 | US | 27 years | 1990 | 2016 | Air pollution (PM) |
| 375 | Wrong metric (No attributable fraction) | Song et al.; 2025 | China | 3 years | 2017 | 2020 | Air pollution (various) |
| 386 | Wrong outcome | Maleki et al.; 2023 | Iran | 2 years | 2020 | 2022 | Air pollution (various) |
| 349 | Data from another study | Tunesi et al., 2024 | Italy (Milan) | 1 year | 2019 | 2019 | Air pollution (PM) |
| 401 | Duplication | Li, J et al.; 2023 | China | 7 years | 2013 | 2019 | Air pollution (PM and ozone) |
| 478 | Wrong outcome | Wen et al.; 2025 | Multi country (7 countries) | 34 years | 1986 | 2019 | Temperature, humidity |
| 503 | Wrong outcome | Feng et al.; 2024 | China | 4 years | 2008 | 2011 | Air pollution (PM) |
| 417 | Wrong metric (No attributable fraction) | Ma et al.; 2023 | China | 2 years | 2015 | 2016 | Air pollution (PM, O3) |
| 436 | Wrong metric (No attributable fraction) | Bai et al. | China | 20 years | 2001 | 2019 | Air pollution (PM) |
| 535 | Wrong metric (No attributable fraction) | Li et al.; 2023 | China | 3 years | 2015 | 2019 | Air pollution (PM) |
| 502 | Duplication | Aboubakri et al., 2023 | Iran | 18 years | 2005 | 2022 | Heat |

## S8. Quality assessment

### Time series or case crossover

| Study ID | Author | 1. item: quality of exposure (e.g., temperature) measurement (0-1 points). | 2. item: verification of the outcome (0-1 points). | 3. item: level of adjustment for confounding (0-3 points). | Grade | Inclusion |
| --- | --- | --- | --- | --- | --- | --- |
| 2 | Aboubakri et al. | 1 | 1 | 3 | high | x |
| 4 | Achebak et. al. | 1 | 1 | 2 | moderate | x |
| 5 | Adegboye et al. | 1 | 1 | 1 | moderate | x |
| 6 | Adegboye et al. | 1 | 1 | 2 | moderate | x |
| 7 | Al-Hemoud et. al. | 1 | 0 | 2 | low | - |
| 8 | Allen et. al. | 1 | 1 | 1 | moderate | x |
| 17 | Bao et al. | 1 | 1 | 3 | high | x |
| 19 | Bernstein et al. | 1 | 1 | 3 | high | x |
| 20 | Bodor et. al. | 1 | 0 | 1 | low | - |
| 25 | Cao et al. | 1 | 1 | 3 | high | x |
| 27 | Chen et al, | 1 | 1 | 2 | moderate | x |
| 29 | Chen et al. | 1 | 1 | 3 | high | x |
| 30 | Chen et. al. | 1 | 1 | 3 | high | x |
| 31 | Chen et al. | 1 | 1 | 3 | high | x |
| 33 | Cheng et. al. | 1 | 1 | 2 | moderate | x |
| 37 | Crank et al. | 1 | 1 | 1 | moderate | x |
| 38 | Cui et el. | 1 | 1 | 0 | low | - |
| 40 | Dabrowieck et. al. | 1 | 1 | 3 | high | x |
| 42 | Dang et. al. | 1 | 1 | 3 | high | x |
| 46 | Deng et al. | 1 | 1 | 2 | moderate | x |
| 47 | Denpetkul et Phosri | 1 | 1 | 2 | moderate | x |
| 53 | Fang et al. | 1 | 1 | 3 | high | x |
| 54 | Fang et. al. | 1 | 1 | 3 | high | x |
| 59 | Ferreira et. al. | 1 | 1 | 3 | high | x |
| 61 | Fu et. al. | 1 | 1 | 2 | moderate | x |
| 63 | Gasparrini et. al. | 1 | 1 | 3 | high | x |
| 71 | Han et al. | 1 | 1 | 2 | moderate | x |
| 72 | He et. al. | 1 | 1 | 3 | high | x |
| 73 | He et. al. | 1 | 1 | 3 | high | x |
| 74 | He et. al. | 1 | 1 | 3 | high | x |
| 77 | Hu et. al. | 1 | 1 | 3 | high | x |
| 79 | Hu et. al. | 1 | 1 | 3 | high | x |
| 80 | Huang et. al. | 1 | 1 | 3 | high | x |
| 81 | Huang et. al. | 1 | 1 | 3 | high | x |
| 82 | Huber et. al. | 1 | 1 | 2 | moderate | x |
| 83 | Ingole et. al. | 1 | 1 | 1 | moderate | x |
| 84 | Iñiguez et. al. | 1 | 1 | 3 | high | x |
| 86 | Jaffe et. al. | 1 | 1 | 3 | high | x |
| 87 | Ji et. al. | 1 | 1 | 3 | high | x |
| 88 | Jiang et. al. | 1 | 1 | 3 | high | x |
| 89 | Jiao et. al. | 1 | 1 | 2 | moderate | x |
| 92 | Kephart et al. | 1 | 1 | 0 | low | - |
| 95 | Kim et. al. | 1 | 1 | 3 | high | x |
| 99 | Lan et. al. | 1 | 1 | 3 | high | x |
| 101 | Lee et. al. | 1 | 1 | 3 | high | x |
| 102 | Lee et. al. | 1 | 1 | 3 | high | x |
| 104 | Lee et. al. | 1 | 1 | 3 | high | x |
| 104.5 | Lee et. al. | 1 | 1 | 3 | high | x |
| 105 | Lei et. al. | 1 | 1 | 3 | high | x |
| 107 | Li et. al. | 1 | 1 | 3 | high | x |
| 111 | Li et. al. | 1 | 1 | 3 | high | x |
| 114 | Lin et. al. | 1 | 1 | 3 | high | x |
| 116 | Liu et. al. | 1 | 1 | 3 | high | x |
| 117 | Liu et. al. | 1 | 1 | 1 | moderate | x |
| 118 | Liu et. al. | 1 | 1 | 3 | high | x |
| 119 | Liu et al. | 1 | 1 | 3 | high | x |
| 122 | Liu et. al. | 1 | 1 | 3 | high | x |
| 126 | Lu et. al. | 1 | 1 | 3 | high | x |
| 136 | Ma et al. | 1 | 1 | 1 | moderate | x |
| 138 | Ma et. al. | 1 | 1 | 3 | high | x |
| 146 | Martínez-Solanas and Basagaña | 1 | 1 | 3 | high | x |
| 147 | Martínez-Solanas et. al. | 1 | 1 | 3 | high | x |
| 148 | Martínez-Solanas et al. | 1 | 1 | 2 | moderate | x |
| 150 | Mascarenhas et. al. | 1 | 1 | 2 | moderate | x |
| 156 | Nguyen et. al. | 1 | 1 | 2 | moderate | x |
| 160 | Onozuka et. al. | 1 | 1 | 2 | moderate | x |
| 161 | Ordanovich et. al. | 1 | 1 | 2 | moderate | x |
| 163 | Pan et. al. | 1 | 1 | 2 | moderate | x |
| 164 | Park et al. | 1 | 1 | 3 | high | x |
| 167 | Pascal et. al. | 1 | 1 | 2 | moderate | x |
| 167.5 | Pattenden et. al. | 1 | 1 | 3 | high | x |
| 169 | Petkova et. al. | 1 | 1 | 1 | moderate | x |
| 171 | Qiu et. al. | 1 | 1 | 3 | high | x |
| 172 | Qiu et. al. | 1 | 1 | 1 | moderate | x |
| 173 | Qiu et al. | 1 | 1 | 3 | high | x |
| 174 | Qiu et. al. | 1 | 1 | 3 | high | x |
| 175 | Qu et al. | 1 | 1 | 3 | high | x |
| 184 | Scovronick et. al. | 1 | 1 | 2 | moderate | x |
| 185 | Sepandi et. al. | 1 | 1 | 2 | moderate | x |
| 186 | Sera et. al. | 1 | 1 | 1 | moderate | x |
| 191 | Su et al. | 1 | 1 | 0 | low | - |
| 192 | Su et. al., | 1 | 1 | 2 | moderate | x |
| 193 | Su et. al. | 1 | 1 | 3 | high | x |
| 201 | Tong et al. | 1 | 1 | 2 | moderate | x |
| 204 | Ugalde-Resano et. al. | 1 | 1 | 1 | moderate | x |
| 205 | Urban et. al. | 1 | 1 | 1 | moderate | x |
| 207 | Vicedo-Cabrera et. al. | 1 | 1 | 3 | high | x |
| 208 | Videco-Cabrera et. al. | 1 | 1 | 2 | moderate | x |
| 209 | Wang et. al. | 1 | 1 | 3 | high | x |
| 213 | Wang et al. | 1 | 1 | 3 | high | x |
| 214 | Wang et. al. | 1 | 1 | 1 | moderate | x |
| 216 | Wen et. al. | 1 | 1 | 2 | moderate | x |
| 217 | Wen et. al. | 1 | 1 | 2 | moderate | x |
| 219 | Wu et. al. | 1 | 1 | 3 | high | x |
| 221 | Wu et. al. | 1 | 1 | 3 | high | x |
| 222 | Wu et. al. | 1 | 1 | 2 | moderate | x |
| 224 | Wu et. al. | 1 | 1 | 1 | moderate | x |
| 227 | Xu et. al. | 1 | 1 | 2 | moderate | x |
| 228 | Xu et. al. | 1 | 1 | 1 | moderate | x |
| 229 | Xu et. al. | 1 | 1 | 2 | moderate | x |
| 230 | Xu et. al. | 1 | 1 | 2 | moderate | x |
| 231 | Yan et. al. | 1 | 1 | 2 | moderate | x |
| 233 | Yang et. al. | 1 | 1 | 2 | moderate | x |
| 234 | Yang et. al. | 1 | 1 | 1 | moderate | x |
| 238 | Ye et. al. | 1 | 1 | 1 | moderate | x |
| 239 | Yim et. al. | 1 | 1 | 2 | moderate | x |
| 240 | Yi et. al. | 1 | 1 | 1 | moderate | x |
| 241 | Yu et. al. | 1 | 1 | 2 | moderate | x |
| 242 | Yu et. al. | 1 | 1 | 1 | moderate | x |
| 243 | Zhan et. al. | 1 | 1 | 2 | moderate | x |
| 244 | Zhan et. al. | 1 | 1 | 3 | high | x |
| 246 | Zhang et. al. | 1 | 1 | 2 | moderate | x |
| 248 | Zhang et. al. | 1 | 1 | 2 | moderate | x |
| 250 | Ying et. al. | 1 | 1 | 3 | high | x |
| 252 | Zhang et. al. | 1 | 1 | 2 | high | x |
| 253 | Zhao et. al. | 1 | 1 | 2 | moderate | x |
| 254 | Zhao et. al. | 1 | 1 | 1 | high | x |
| 255 | Zhao et. al. | 1 | 1 | 2 | moderate | x |
| 256 | Zhao et. al. | 1 | 1 | 3 | high | x |
| 260 | Zhou et. al. | 1 | 1 | 3 | high | x |
| 262 | Corvetto et al. | 1 | 1 | 2 | moderate | x |
| 263 | He et al. | 1 | 1 | 3 | high | x |
| 266 | Huang et al. | 1 | 1 | 2 | moderate | x |
| 268 | Wang et al. | 1 | 1 | 1 | moderate | x |
| 269 | López-Bueno et al. | 1 | 1 | 2 | moderate | x |
| 270 | Schulte et al. | 1 | 1 | 2 | moderate | x |
| 273 | Gonçalves et al. | 1 | 1 | 3 | high | x |
| 274 | Zhang et al. | 1 | 1 | 2 | moderate | x |
| 275 | Wang et al. | 1 | 1 | 2 | moderate | x |
| 276 | Qiu et al. | 1 | 1 | 3 | high | x |
| 277 | Wang et al. | 1 | 1 | 1 | moderate | x |
| 278 | Zhou et al. | 1 | 1 | 2 | moderate | x |
| 280 | Yang et al. | 1 | 1 | 3 | high | x |
| 281 | Zhu, Q | 1 | 1 | 2 | moderate | x |
| 282 | Zhai, C | 1 | 1 | 2 | moderate | x |
| 283 | Tao, J | 1 | 1 | 2 | moderate | x |
| 284 | Chen, J | 1 | 1 | 2 | moderate | x |
| 285 | Zhou, E | 1 | 1 | 2 | moderate | x |
| 286 | Huang, W | 1 | 1 | 1 | moderate | x |
| 287 | Wang, H | 1 | 1 | 2 | moderate | x |
| 288 | Chen, G | 1 | 1 | 2 | moderate | x |
| 290 | Feng, Y | 1 | 1 | 2 | moderate | x |
| 291 | Zhang, R | 1 | 1 | 2 | moderate | x |
| 292 | Zhang, H | 1 | 1 | 2 | moderate | x |
| 293 | Zhan, Z.Y | 1 | 1 | 2 | moderate | x |
| 294 | Guo, J | 1 | 1 | 2 | moderate | x |
| 296 | Zhao, C | 1 | 1 | 2 | moderate | x |
| 297 | Xu, R | 1 | 1 | 2 | moderate | x |
| 299 | Jiang, W | 1 | 1 | 2 | moderate | x |
| 300 | Requia, W.J | 0 | 1 | 2 | low | - |
| 301 | Li, Y | 1 | 1 | 2 | moderate | x |
| 302 | Staffogia, M | 1 | 1 | 3 | high | x |
| 303 | Zhang, R | 1 | 1 | 3 | high | x |
| 304 | Lai, H | 0 | 1 | 1 | low | - |
| 305 | Gao, Y | 1 | 1 | 0 | low | - |
| 307 | Boudreault, J | 1 | 1 | 2 | moderate | x |
| 308 | Hu, J | 1 | 1 | 0 | low | - |
| 310 | Mendrinos | 1 | 1 | 2 | moderate | x |
| 311 | Jiang, F | 0 | 1 | 3 | low | - |
| 312 | Sharma, A | 1 | 1 | 2 | moderate | x |
| 313 | Qing, M | 1 | 1 | 3 | high | x |
| 314 | Min, J | 1 | 1 | 1 | moderate | x |
| 315 | Thawonmas, R | 1 | 1 | 2 | moderate | x |
| 316 | Tao, J | 1 | 1 | 2 | moderate | x |
| 317 | Tang, W | 1 | 1 | 2 | moderate | x |
| 318 | Wu, C | 0 | 1 | 2 | low | - |
| 319 | Wen, B | 1 | 1 | 2 | moderate | x |
| 324 | Dimitrova et al. | 1 | 1 | 3 | high | x |
| 329 | Tobías et al. | 1 | 1 | 1 | moderate | x |
| 330 | Scrovonick et al. | 1 | 1 | 1 | moderate | x |
| 331 | Ascaso et al. | 1 | 1 | 3 | high | x |
| 336 | Xia, Y | 1 | 1 | 2 | moderate | x |
| 337 | Niu, S | 1 | 1 | 2 | moderate | x |
| 340 | Huang, Z | 1 | 1 | 2 | moderate | x |
| 344 | Han et al. | 0 | 1 | 3 | low | - |
| 348 | Li, W | 0 | 1 | 3 | low | - |
| 354 | Bai, L | 1 | 1 | 2 | moderate | x |
| 357 | Wang et al. | 0 | 1 | 2 | low | - |
| 359 | Xu et al. | 1 | 1 | 3 | high | x |
| 360 | Yang et al. | 1 | 1 | 3 | high | x |
| 361 | Yang et al. | 1 | 1 | 3 | high | x |
| 362 | Chen et al. | 1 | 1 | 2 | moderate | x |
| 365 | Yezli et al. | 1 | 1 | 3 | high | x |
| 376 | Fatima | 1 | 1 | 3 | high | x |
| 377 | Li | 1 | 1 | 2 | moderate | x |
| 379 | Alwadi | 0 | 1 | 2 | low | - |
| 380 | Wei et al. | 1 | 1 | 2 | moderate | x |
| 389 | Jingesi | 1 | 1 | 3 | high | x |
| 391 | Jiang | 1 | 1 | 3 | high | x |
| 404 | Lin | 1 | 1 | 2 | moderate | x |
| 406 | Rhamati, S | 1 | 1 | 2 | moderate | x |
| 414 | Xu, J | 1 | 1 | 2 | moderate | x |
| 418 | Mei, F | 0 | 1 | 0 | low | - |
| 424 | Li, X | 0 | 1 | 2 | low | - |
| 426 | Li et al. | 1 | 1 | 3 | high | x |
| 431 | Wen | 1 | 1 | 2 | moderate | x |
| 432 | Wu | 1 | 1 | 2 | moderate | x |
| 434 | López-Bueno | 1 | 1 | 3 | moderate | x |
| 444 | Janos | 1 | 1 | 2 | moderate | x |
| 469 | Zheng-Dong et al. | 1 | 1 | 2 | moderate | x |
| 470 | Zhao et al. | 1 | 1 | 3 | high | x |
| 476 | Ji et al. | 1 | 1 | 3 | high | x |
| 477 | Li et al. | 1 | 1 | 3 | high | x |
| 486 | Borg et al. | 1 | 1 | 2 | moderate | x |
| 487 | Hu et al. | 1 | 1 | 2 | moderate | x |
| 489 | He et al. | 1 | 1 | 2 | moderate | x |
| 498 | Ngyuen | 1 | 1 | 3 | high | x |
| 505 | Psistaki | 1 | 1 | 2 | moderate | x |
| 532 | Jiang et al. | 1 | 1 | 3 | high | x |

### Cohort and case-control studies

| Study ID | Author | 1. Selection (0-4 points) | 2. Comparability (0-2 points) | 3. Outcome (0-3 points) | Grade | Inclusion |
| --- | --- | --- | --- | --- | --- | --- |
| 249 | Zhang et al. | 1. Representativeness: truly representative.  2. Selection of the non-exposed: no description of the derivation of the non-exposed cohort.  3. Ascertainment of exposure: secure record.  4: Demonstration that outcome of interest was not present at the start of the study: yes.  Result: 3 | 1. Comparability of cohorts on the basis of the design or analysis controlled for confounders: temporal trends, seasonality, sex, maternal age, previous pregnancy, number of births, medical condition, smoking status, and remoteness of maternal residence.  Result: 2 | 1. Assessment of outcome: record linkage.  2. Follow up long enough: yes.  3. Adequacy of follow-up: complete follow-up.  Result: 3 | high | x |
| 189 | Singh et al. | 1. Representativeness: truly representative.  2. Selection of the non-exposed: drown from the same communities.  3. Ascertainment of exposure: structured interview.  4: Demonstration that outcome of interest was not present at the start of the study: not applicable.  Result: 3 | 1. Comparability of cohorts on the basis of the design or analysis controlled for confounders: seasonality, time trends, socioeconomic, and anthropometric variables.  Result: 2 | 1. Assessment of outcome: independent blind assessment.  2. Follow up long enough: yes.  3. Adequacy of follow-up: not available – 100%?  Result: 2 | high | x |
| 276 | Qiu et al. | 1. Representativeness: truly representative.  2. Selection of the non-exposed: drown from the same communities.  3. Ascertainment of exposure: structured interview.  4: Demonstration that outcome of interest was not present at the start of the study: yes.  Result: 4 | 1. Comparability of cohorts on the basis of the design or analysis controlled for confounders: season of delivery, maternal age, delivery mode, newborn gender, history of adverse pregnancy outcomes, active smoking, husband smoke and alcohol drinking status during the early stage of pregnancy.  Result: 2 | 1. Assessment of outcome: record linkage.  2. Follow up long enough: yes.  3. Adequacy of follow-up: subject lost less than 20%.  Result: 2 | high | x |
| 371 | Xue et al. | 1. Case definition adequate? Yes, with independent validation.  2. Representativeness of the cases: all cases in the HDS.  3. Selection of controls: from the same family.  4: Definition of control: yes.  Result: 4 | 1. Comparability of cases and control: for previous injury and for age.  Result: 2 | 1. Ascertainment of exposure: interview not blinded to case/control status.  2. Same method of ascertainment for cases and controls: yes.  3. Non-response rate: same for both groups.  Result: 2 | high | x |
| 408 | Kang et al. | 1. Case definition adequate? Yes, with independent validation.  2. Representativeness of the cases: all eligible cases in the sample.  3. Selection of controls: from the same source population.  4: Definition of control: yes.  Result: 4 | 1. Comparability of cases and control: for previous injury and for age.  Result: 2 | 1. Ascertainment of exposure: structured injury data,  2. Same method of ascertainment for cases and controls: yes.  3. Non-response rate: no information on how that differ between cases and controls.  Result: 2 | high | x |

### Quasi-Experimental Studies

JBI Critical Appraisal Checklist

| Study author and (ID) | Su et al. (193) |
| --- | --- |
| 1. Is it clear in the study what is the ‘cause’ and what is the ‘effect’ (i.e. there is no confusion about which variable comes first)? | Yes |
| 2. Were the participants included in any comparisons similar? | Yes |
| 3. Were the participants included in any comparisons receiving similar treatment/care, other than the exposure or intervention of interest? | Yes |
| 4. Was there a control group? | No |
| 5. Were there multiple measurements of the outcome both pre and post the intervention/exposure? | Yes |
| 6. Was follow up complete and if not, were differences between groups in terms of their follow up adequately described and analyzed? | Yes |
| 7. Were the outcomes of participants included in any comparisons measured in the same way? | Yes |
| 8. Were outcomes measured in a reliable way? | Yes |
| 9. Was appropriate statistical analysis used? | Yes |
| Score (total) | Good |
| Papers are considered Good, Fair or Poor by the authors, guided by this quality tool. The JBI provides no cut off point, but does guide authors into a better decision making. Poor studies are excluded. | The quality is good and the study will be included. |

## S9. Narrative results: results synthesis of morbidity and mortality.

| **Table 1a – Descriptive results: AFs of climate-related mortality for 9 subgroup diseases.** | |
| --- | --- |
| Risk factor AF in % (SD / CI) | Risk factor AF in % (SD / CI) |
| All-cause mortality (A00–Z99)   \| Heat  T_mean_ > MMT \| 1.60% (SD: 1.46)^1-15^  3.58% (3.20; 3.96)^16^  0.7% (SD: 0.29)^17-19^  0.64% (0.19; 1.09)^20^  0.45% (SD: 0.49)^5,21,22^  0.70% (SD: 0.29)^15,20^  0.72% (SD: 1.30)^6,9,21-24^  0.3% (0.1; 0.4)_elderly_^169^  0.11% (-0.06; 0.30)^18^  1.54% (SD: 1.68)^25,26^  4.76% (SD: 5.23)^23,26-32^  2.09% (SD: 0.62)^31,33^  3.79% (3.14; 4.45)^34^  1.89% (SD: 1.12)^35-37^  1.72% (SD: 1.92)^35,37^  4.08% (3.71; 4.45)^36^  0.85% (0.34; 1.37)^36^  1.82% (SD: 1.44)^36,37^  0.58% (0.31; 0.85)^38^  −0.39% (-1.49; 0.73)^39^ _children_  −0.019% (SD: 0.070)^40^  1.28% (1.27; 1.29)^41^  9.86% (SD: 7.21)^42^ \| \| --- \| --- \| \| T_max_ > 85^th^ \| \| T_mean_ > 90^th^  Moderate heat  95^th^ > T_mean_ > MMT  97.5^th^ > T_mean_ > MMT  Extreme heat  T_mean_ > 95^th^  T_mean_ > 97.5^th^  T_mean_ > 99^th^  Temperature variability  Interday TV  Intraday TV  Hourly TV  Air pollution  PM2.5 > WHO  Per 10ug/m^3^  PM2.5  PM10  CO  NO2  O3  Wildfire-related O3  Extreme events  Extreme precipitation  Floods  Heatwaves  (various definitions) \|   Infectious diseases (A00–B99)   \| Moderate heat  97.5^th^ > T_mean_ > MMT  Extreme heat  T_mean_ > 97.5^th^ \| 1.50% (1.3; 1.6)_leishmaniasis_^43^  1.70% (1.6; 1.8)_leishmaniasis_^43^ \| \| --- \| --- \|   Neoplasms (C00–D99)   \| Interday TV  (per 1° increase) \| 4.29% (2.02; 6.65)^44^ \| \| --- \| --- \|   Endocrine, nutritional, metabolic (E00–E90)   \| Heat  T_mean_ > MMT  T_mean_ > 90^th^ \| 0.47% (-1.40; 1.86)_diabetes_^18^  10.67% (-14.03; 26.24)_diabetes_^18^ \| \| --- \| --- \|   Mental, behavioral (F00–F99)   \| Heat  T_mean_ > 90^th^  Extreme heat  T_mean_ > 99^th^  Air pollution  PM2.5 > WHO  PM10 > WHO \| 4.49% (0.54; 7.84)^18^  0.22% (-0.98; 1.22)^18^  6.92% (-)^45^  6.99% (-)^45^ \| \| --- \| --- \|   Nervous system (G00-G99)   \| Air pollution  PM2.5 > WHO  PM10 > WHO  Extreme events  Heatwaves  (various definitions) \| 4.21% (-)_dementia_^45^  4.43% (-)_dementia_^45^  12.3% - 31.5% _alzheimer and other dementias_^46^ \| \| --- \| --- \|   Genitourinary system (N00-N99)   \| Air pollution  PM2.5 > WHO  O3 > WHO  NO2 > WHO \| 1.9% (-)^47^  6.3% (-)^47^  5.2% (-)^47^ \| \| --- \| --- \|   Injury-related   \| Extreme events  Heatwaves \| 11.4% (10.0; 12.9)^48^ \| \| --- \| --- \| | Cardiovascular (E00–E90)   \| Heat  T_mean_ > MMT  T_mean_ > 90^th^  Moderate heat  97.5^th^ > T_mean_ > MMT  Extreme heat  T_mean_ > 97.5^th^  T_mean_ > 99^th^  Temperature variability  Interday TV  Intraday TV  Hourly TV  Air pollution  PM2.5 > WHO  Per 10ug/m^3^  PM2.5  PM10  O3  Wildfire-related O3  Extreme events  Floods  Heatwaves  (various definitions) \| 2.05% (SD: 1.12)^5-7,49-53^  0.07% (-0.13; 0.28)^18^  1.00% (-0.10; 2.00)_IHD_^54^  0.94% (SD: 0.59)^5,21,23^  0.20% (-0.20; 0.50)_IHD_^21^  0.85% (SD: 0.07)_MI_^55,56^  0.15% (-0.25; 0.51)^18^    0.44% (0.28; 0.60)^26^  5.01% (SD: 6.88)^26^  1.89% (0.43; 3.34)^31^  4.46% (SD: 1.15)^50^  1.83% (SD: 1.69)^35,37^  1.96% (SD: 2.24)^35,37^  0.85% (0.75; 0.96)^37^  0.41% (-0.15; 0.91)^38^  0.017% (SD: 0.18%)^40^  1.18%-1.74%^57^ \| \| --- \| --- \|   Strokes (I60–I64)   \| Heat  T_mean_ > MMT  Moderate heat 97.5^th^ > T_mean_ > MMT  Extreme heat  T_mean_ > 97.5^th^  Air pollution  Per 10ug/m^3^  PM2.5  PM10 \| 2.83% (SD: 1.33)^51,58,59^  0.10% (-0.00; 0.10)^61^  0.40% (0.00; 0.70)^61^  2.98% (0.89; 5.01)^79^  3.46% (1.24; 5.61)^79^ \| \| --- \| --- \|   Respiratory (J00–J99)   \| Heat  T_mean_ > MMT  T_mean_ > 90^th^  Moderate heat  97.5^th^ > T_mean_ > MMT  Extreme heat  T_mean_ > 97.5^th^  T_mean_ > 99^th^  Temperature variability  Interday TV  Intraday TV  Hourly TV  Air pollution  Per 10ug/m^3^  PM2.5  PM10  O3  Wildfire-related O3  Extreme events  Floods \| 2.71% (SD: 0.67)^5-7,50^  3.30% (0.50; 5.70)_asthma_^60^  0.06% (-0.14; 0.26)^18^  1.62% (SD: 0.73)^5,21^  2.40% (SD: 3.93)^5,6,21,23,61^  0.15% (-0.26; 0.53)^18^  0.44% (0.28; 0.60)^26^  3.55% (SD: 3.88)^23,26,29,31^  1.89% (0.43; 3.34)^31^  1.46% (SD: 1.07)^35,37^  1.55% (SD: 1.48)^35,37^  2.08% (SD: 1.13)^37,62^  0.86% (0.18; 1.51)^38^  0.041% (SD: 0.38%)^40^ \| \| --- \| --- \|   Suicides (X60–I84)   \| Moderate heat  97.5^th^ > T_mean_ > 50^th^  Extreme heat  T_mean_ > 95^th^ \| 9.9% (9.4; 10.4)^63^  31.7% (18.0; 43.2)^64^ \| \| --- \| --- \| |

Proportion of mortality attributable to climate-related exposures, stratified by ICD-10 disease groupings or more specific health outcomes. MI: myocardial infarction, IHD: ischemic heart disease. T_mean_: mean temperature; T_max_: maximum temperature; MMT: minimum mortality temperature; WHO: limit of air pollutants established by the World Health Organization; TV: temperature variability.

| **Table 1b – Descriptive results: AFs of climate-related morbidity for 15 subgroup diseases.** | |
| --- | --- |
| Risk factor AF in % (SD / CI) | Risk factor AF in % (SD / CI) |
| All-cause morbidity (A00–Z99)   \| Heat  T_mean_ > MMT  T_max_ > MMT  Moderate heat  97.5^th^ > T_mean_ > MMT  Extreme heat  T_mean_ > 95^th^  T_mean_ > 97.5^th^  Temperature variability  Intraday TV  Air pollution  PM2.5 > WHO  Per 10ug/m^3^  PM1  Wildfire-related O3 \| 0.1% (0.1; 0.1)_HA_^15^  3.47% (SD: 1.65)_EDV_^65^  4.90% (0.90; 8.30)_EAD_^66^  1.1% (0.7; 1.4)_EDV_^67^  4.50% (3.62; 5.19)_HA_^68^  0.1% (0.0; 0.1)_HA_^15^  0.4% (0.4; 0.4)_EDV_^15^  0.60% (0.00; 1.10)_OV_ ^69^  0.96% (0.86; 1.02)_HA_^68^  2.78% (SD: 1.03)_HA_^70,71^  1.09% (0.69; 1.49)_EDV_^72^  0.11% (0.01; 0.22)_HA_^73^  0.53% (0.48; 0.58)_HA_^74^ \| \| --- \| --- \|   Infectious diseases (A00–B99)   \| Heat  T_mean_ > MMT  T_max_ > MMT  Extreme heat  T_mean_ > 97.5^th^  Temperature variability  Interday TV  Intraday TV  Extreme events  Floods  Tropical cyclones \| 7.46% (5.55; 9.32)_prevalence, diarrhea, children_^75^  4.7% (3.3; 6)_EDV_^67^  0.99% (0.57; 1.29)_incidence, diarrhea_^76^  2.79% (-1.42; 6.73)_HA, tuberculosis_^77^  13.09% (5.47; 19.90)_HA, tuberculosis_^77^  0.25% (0.10; 0.36)_incidence, diarrhea_^78^  1.10% (0.62; 1.50)_incidence, bacillary dystentery_^79^ \| \| --- \| --- \|   Nutritional anemias (D50–D53)   \| Air pollution  Dust-PM2.5 \| 16.73% (15.19; 18.16)_prevalence, anemia, children_^80^ \| \| --- \| --- \|   Endocrine, nutritional, metabolic (E00–E90)   \| Heat  T_max_ > MMT  Temperature variability  Intraday TV  Air pollution  PM2.5 > WHO  PM10 > WHO  NO2 > WHO  Wildfire-PM2.5 \| 0.0% (-0.1; 0.2)_EDV, digestive_^67^  0.91% (-3.07; 3.57)_HA_^71^  1.10% (0.49; 1.71)_HA, endocrine_^81^  0.76% (0.35; 1.16)_HA, diabetes II_^82^  0.33% (0.04; 0.62)_HA, diabetes II_^82^  3.39% (2.26; 4.54)_HA, diabetes II_^82^  0.67% (0.16; 0.18)_HA, diabetes all_^83^ \| \| --- \| --- \|   Mental, behavioral (F00–F99)   \| Heat  T_mean_ > MMT  T_max_ > MMT  T_mean_ > 90^th^  Moderate heat  99^th^ > T_mean_ > 95^th^  Extreme heat  T_mean_ > 99^th^  Temperature variability  Intraday TV  Air pollution  PM_2.5_ > WHO  PM_10_ > WHO  NO_2_ > WHO  CO > WHO  O_3_ > WHO  Extreme events  Heatwaves  Extreme precipitation \| 7.41% (3.97; 10.86)_HA, schizophrenia_^84^  3% (2; 4.1)_EDV_^67^  0.28% (−1.18; 1.78) _HA, schizophrenia_^85^  0.36% (-0.43; 1.13)_EDV_^86^  0.33% (0.16; 0.50)_EDV_^86^  3.22% (0.25; 5.43)_HA_^71^  2.25% (0.79; 3.75)_HA, schizophrenia_^87^  9.53% (2.67; 15.58)_HA_^88^  2.78% (SD: 0.58)_HA, schizophrenia_^89,90^  3.47% (2.76; 4.16)_OV, anxiety_^91^  0.48% (0.26; 0.70)_HA_^92^  3.26% (SD: 0.43)_HA, schizophrenia_^89,90^  4.61% (2.93; 6.32)_HA, schizophrenia_^90^  2.02% (0.39; 3.68) _HA, schizophrenia_^90,91^  2.62% (1.49; 3.71)_OV, anxiety disorders_^91^  0.03% (0.01; 0.06)_HA, schizophrenia_^93^  5.00% (1.02; 8.82)_HA, depressions_^94^  0.02% (-0.02; 0.06)_HA, schizophrenia_^93^ \| \| --- \| --- \|   Nervous system (G00–G99)   \| Heat  T_mean_ > MMT  Air pollution  PM_2.5_ > WHO \| 0.4% (0.0; 0.8)_EDV_^67^  0.68% (0.12; 1.22)_HA_^88^ \| \| --- \| --- \|   Genitourinary (N00–N99)   \| Heat  T_mean_ > MMT  T_max_ > MMT  Temperature variability  Intraday TV  Air pollution  PM_2.5_ > WHO  PM_10_ > WHO  NO_2_ > WHO  O_3_ > WHO  NO_2_ per 10ug/m^3^  Extreme events  Heatwaves \| 7.4% (5.2; 9.6)_HA, renal diseases_^95^  2.1% (0.7; 3.4)_EDV_^67^  7.46% (1.48; 10.88)_HA_^71^  0.81% (0.41; 1.20)_HA_^81^  2.83% (1.92; 3.74)_HA, CKD_^96^  3.46% (1.92; 3.74)_HA, CKD_^96^  2.1% (0.6; 3.7)_OV, acute kidney injuries_^97^  16.8% (1.1; 29.9)OV_, acute kidney injuries_^97^  5.75% (0.60; 10.63)_HA, urolithiasis_^98^  8.30% (4.80; 11.60)_HA_^99^ \| \| --- \| --- \| | Cardiovascular diseases (I00–I99)   \| Heat  T_mean_ > MMT  Moderate heat  97.5^th^ > T_mean_ > MMT  Extreme heat  T_mean_ > 95^th^  T_mean_ > 97.5^th^  T_mean_ > 99^th^  Temperature variability  Intraday TV  Air pollution  PM2.5 > WHO  PM10 > WHO  O3 per 10ug/m^3^  Extreme events  Extreme precipitation \| 3.8% (-0.4; 8.1)_HA_^100^  0.6% (SD: 0.43)_OHCA_^101,102^  0.10% (0.06; 0.14)_OHCA_^101^  -0.3% (-1.0; 0.4)_cases, MI_^103^  3% (-0.60; 6.30)_HA_^100^  0.8% (0.1; 1.40)_HA_^100^  0% (-0.3; 0.3)_cases, MI_^103^  0.20% (0.15; 0.23)_OHCA_^101^  9.18% (2.03; 13.90)_HA_^71^  0.95% (SD: 0.09)_HA_^81,92^  13.0% (10.5; 15.4)_HA, acute aortic dissections_^104^  0.48% (0.26; 0.7)_HA_^92^  6.1% (4.7; 7.4)_HA, acute aortic dissections_^104^  11.66% (7.66; 15.4)_HA, MI_^105^  0.68% (0.20; 1.12)_HA, MI_^106^ \| \| --- \| --- \|   Strokes (I60–I64)   \| Heat  T_mean_ > MMT  Temperature variability  Intraday TV  Air pollution  PM2.5 per 10ug/m^3^  PM2.5 > WHO  PM10 > WHO \| 1.95% (0.63; 3.20)_HA_^107^  3.65% (1.81; 5.53)_HA_^108^  5.60% (4.20; 6.80)_HA_^109^  1.01% (0.45; 1.56)_HA_^92^  3.11% (SD: 3.53)_HA_^92,109^ \| \| --- \| --- \|   Respiratory (J00-J99)   \| Heat  T_mean_ > MMT  T_max_ > MMT  Moderate heat  97.5^th^ > T_mean_ > MMT  Extreme heat  T_mean_ > 97.5^th^  T_mean_ > 99^th^  Temperature variability  Interday TV  Intraday TV    Hourly TV  Air pollution  Per 10ug/m^3^ increase  PM_2.5_  PM_10_  NO_2_    CO  PM_2.5_ > WHO    PM_10_ > WHO  NO_2_ > WHO  Sand-dust PM_10_  Joint pollutants \| 0.90% (-0.50; 2.10)_HA, children_^110^  7.68% (-5.12; 32.21)_HA, pneumonia_^54^  0.4% (-0.4; 1.1)_EDV_^67^  5.50% (3.00; 7.80)_HA, asthma_^3^  0.2% (-0.3; 0.6)_HA_^61^  2.20% (1.30; 3.00)_HA, asthma_^3^  0.09% (0.02; 0.15)_HA, pneumonia_^111^  0.35% (-0.06; 0.61)_HA, children_^112^  3.42% (1.60, 5.14)_acute upper respiratory infections_^113^  1.79% (SD: 2.52)_HA_^71,114^  15.4% (9.2; 21.1)_HA_^115^  24.26% (15.46, 32.05)_acute upper respiratory infections_^311^  12.00% (6.50; 17.10)_HA, COPD_^115^    3.74% (0.29; 7.11)_HA, asthma_^116^  4.46% (2.43; 6.43)_incidence, influenza_^117^  5.03% (2.33; 7.56)_incidence, influenza_^117^  9.70% (−3.40; 21.20)_HA, COPD exacerbation_^118^  23.22% (17.56; 28.61)_incidence, influenza_^117^  1.69% (1.12; 2.24)_HA_^81^  7.33% (4.31; 10.34)_HA, COPD_^119^  4.3% (3.5; 5.2)_HA, children_^120^  6.26% (3.66; 8.86)_HA, COPD_^119^  2.6% (2.0; 3.2)_HA, children_^120^  1.64% (1.06; 2.18)_HA, asthma_^121^  22.4% (17.2; 27.4)_HA, acute aortic dissection_^476^ \| \| --- \| --- \|   Digestive system (K00–K93)   \| Heat  T_mean_ > MMT \| 6.72% (0.43; 12.61)_HA, alcoholic liver disease_^97^ \| \| --- \| --- \|   Skin diseases (L00-L99)   \| Heat  T_mean_ > MMT \| 6.51% (2.84; 9.96)_prevalence, children_^75^  3.0% (0.5; 5.0)_OV, atopic dermatitis_^122^ \| \| --- \| --- \|   Musculoskeletal (M00–M99)   \| Heat  T_mean_ > MMT  Temperature variability  Interday TV  Intraday TV  Air pollution  PM_2.5_ > WHO  PM_10_ > WHO  NO_2_ > WHO  O_3_ > WHO  CO > WHO \| 6.51% (2.84; 9.96)_prevalence, children_^75^  3.0% (0.5; 5.0)_OV, atopic dermatitis_^42^  6.24% (-2.28; 10.69)_HA_^71^  4.01% (-1.49; 9.63)_HA, gout_^58^  4.19% (-27.68; 29.18)_HA, gout_^58^  2.16% (1.45; 2.85)_OV, osteoathritis_^123^  0.58% (0.05; 1.09)_OV, osteoathritis_^123^  3.83% (2.88; 4.74)_OV, osteoathritis_^123^  2.83% (1.43; 4.17)_OV, osteoathritis_^123^  5.11% (4.10; 6.07)_OV, osteoathritis_^123^ \| \| --- \| --- \|   Occupational injuries   \| Heat  T_mean_ > MMT  Moderate heat  97.5^th^ > T_mean_ > MMT  Extreme heat  T_mean_ > 97.5^th^  Extreme events  Heatwaves \| 2.40% (2.09; 2.68)_incidence_^124^  2.24 (0.16; 0.18)_incidence_^124^  0.17% (0.16; 0.18)_incidence_^124^  0.27% (SD: 0.2)_cases_^125,126^ \| \| --- \| --- \|   Perinatal period (P00–P96)   \| Extreme heat  T_mean_ > 95^th^ \| 3.17% (2.58; 3.79)_cases, pre-term birth_^127^ \| \| --- \| --- \|   Injuries, poisoning (S00–T98)   \| Extreme events  Heatwaves \| 6.54% (3.94; 9.01)_HA, injuries_^98^ \| \| --- \| --- \| |

Proportion of morbidity outcomes attributable to climate-related exposures, stratified by ICD-10 disease groupings or more specific health outcomes. HA: hospital admission or hospitalization; EDV: emergency department visits; OV: outpatient visits; EAD: emergency ambulance dispatch; OHCA: out-of-hospital cardiac arrest; MI: myocardial infarction; CKD: chronic kidney disease; COPD: chronic obstructive pulmonary disease; T_mean_: mean temperature; T_max_: maximum temperature; MMT: minimum mortality temperature; WHO: limit of air pollutants established by the World Health Organization; TV: temperature variability.

Several methodologically idiosyncratic analyses—insufficiently comparable with the main evidence base—were omitted from Table 1; their full results are presented in Appendix S6. Approaches other than the WHO air-quality guideline or the conventional 10 µg/m³ increment were excluded from the table—namely the Chinese national standard (CNAAQS)^118,128^, 7.5 µg/m³,^129^ general mean concentration,^130^ 2-year or decadal means,^131,132^ median pollutant values 69, the 25th, 50th, or 90th percentiles of the pollutant distribution,^133-136^ 1 µg/m³ incremental increases,^137^ and interquartile-range increments.^138,139^ Similarly, rarely used climate metrics were excluded, such as AFs per 1 °C change,^140-142^ temperatures above the 50th percentile,^143^ bespoke heat definitions,^97,144,145^ hot-night or hot-day exposures,^146-148^ single-use heat indices,^149,150^ temperature variability expressed per interquartile range,^151^ and unconventional heat-wave definitions.^152^
Vulnerable populations experienced substantial attributable fractions: heat accounted for 27.3% (SD: 31.3)^153^ of deaths among people experiencing homelessness. Two investigations of combined temperature-pollutant exposures reported AFs of 5.31% (4.58; 5.91)^41^ and 16.65% (16.43; 16.87),^36^ highlighting the additive impact of concurrent hazards. One study linked drowning mortality to compound dry-hot and wet-hot conditions.^154,155^ Estimates based solely on warm-season data—potentially inflating annual risk—were identified in several reports.^140,142,156-163^ Additional findings included pneumonia-specific AFs of 1.3–2.0%^164^ and a 4.96% (3.16; 6.66)^53^ AF for cardiorespiratory mortality attributable to moderate heat.

## S10. Sensitivity analysis Meta-Analysis.

|  | Number of studies included | Pooled effect size | CI (95%) | I2 (p-value) | τ2 (SE) | Q test (p-value) | Egger's test p-value | Outlying studies | Excluded outlying studies + reason |
| --- | --- | --- | --- | --- | --- | --- | --- | --- | --- |
| All-cause mortality | 17 - **1** | 1.18 | (1.01-1.37) | 99.99% (<0.0001) | 0.0003 (0.0002) | 261435.2676 (<0.0001) | p <0.0001 | Ma et al., 2020, Chen et al., 2018, Yezli et al., 2023 | **Yezli et al., 2023** – strongest outlier and highly influential over the pooled effect size with risk of overestimation. |
| All-cardiovascular mortality | 10 | 2.15 | (1.54-2.88) | 99.98% (<0.0001) | 0.0014 (0.0009) | 46517.2650 (<0.0001) | p = 0.6927 | Ma et al., 2020 | _ |
| All-respiratory mortality | 6 - **1** | 3.08 | (2.17-4.15) | 99.82% (<0.0001) | 0.0011 (0.0009) | 2268.5472 (<0.0001) | p = 0.7116 | Iñiguez et al., 2021 | **Iñiguez et al.,** **2021** – strong outlier and highly influential over the pooled effect size, with risk of overestimation. |
| Stroke mortality | 6 - **1** | 2.71 | (1.85-3.73) | 99.94% (<0.0001) | 0.0011 (0.0009) | 6261.46 (<0.0001) | p = 0.041 | Ma et. al., 2020 | **Ma et al., 2020** – strongest outlier and highly influential over the pooled effect size with risk of overestimation. |
| All-cause mortality – extreme heat | 4 | 0.45 | (0.22-0.76) | 99.95% (<0.0001) | 0.0004 (0.0004) | 6272.17 (<0.0001) | p = 0.5121 | _ | _ |
| All-cardiovascular mortality – extreme heat | 3 – **2** (no meta-analysis possible) | 0.64 | (0.32-1.06) | 99.91% (<0.0001) | 0.0004 (0.0005) | 2343.63 | p <0.0001 | Janos et al., 2024, Zhang et al., 2019 | **Janos et al., 2024, Zhang et al., 2019** – both outliers were highly influential in opposite directions from the pooled effect size. |

## S11. Sensitivity analysis – meta-analysis performed after excluding outlying studies.


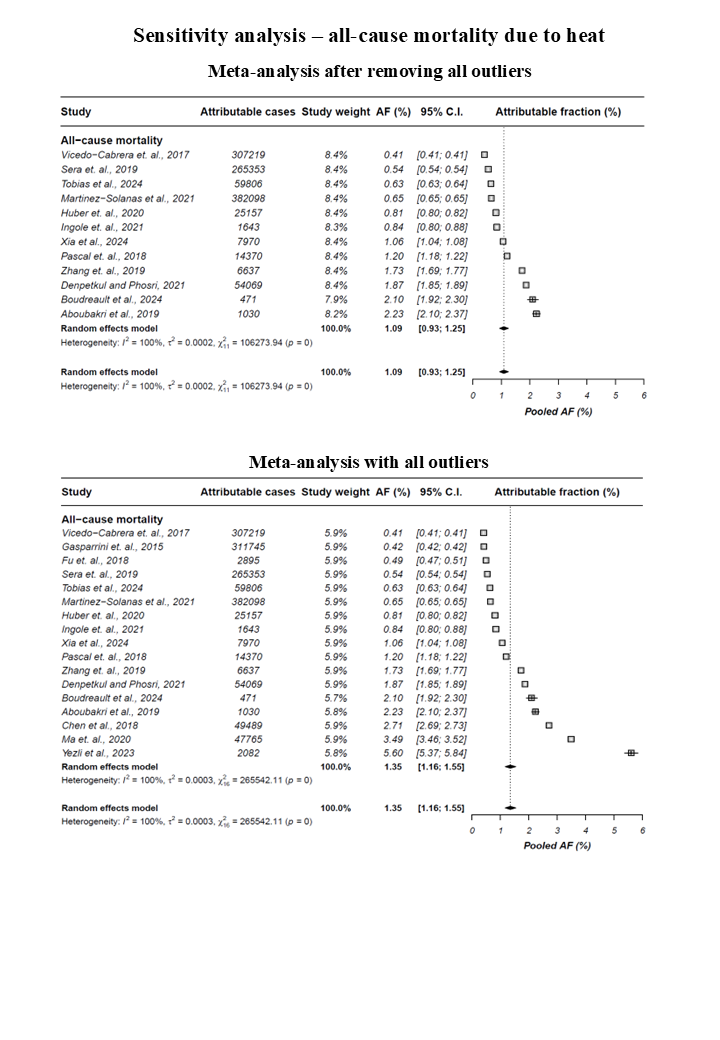


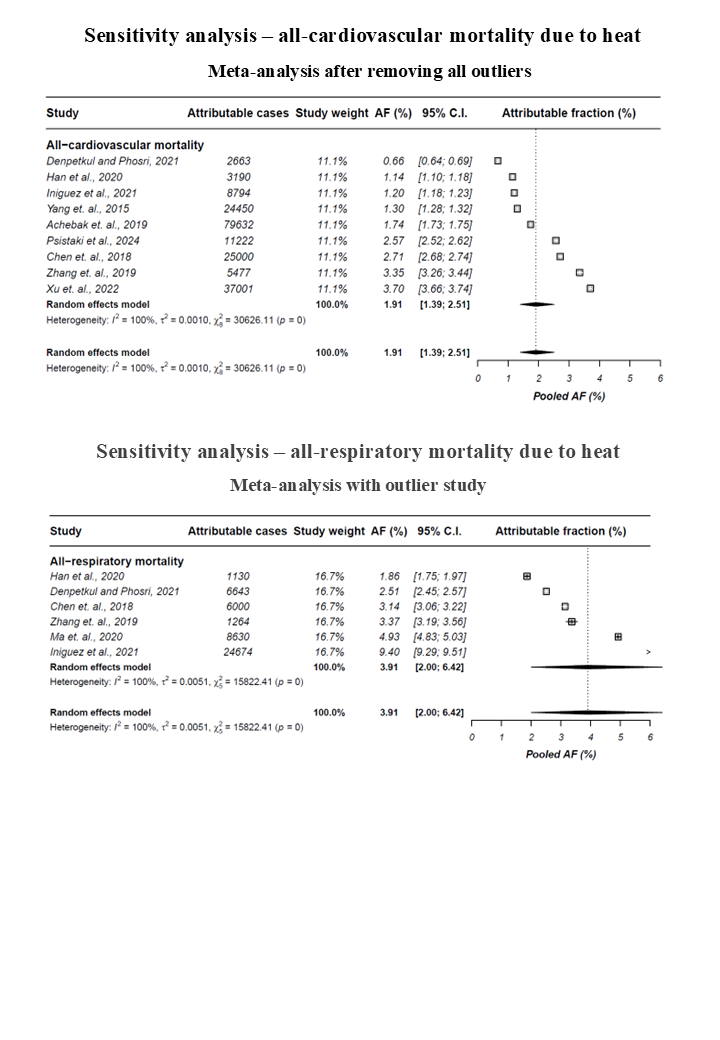


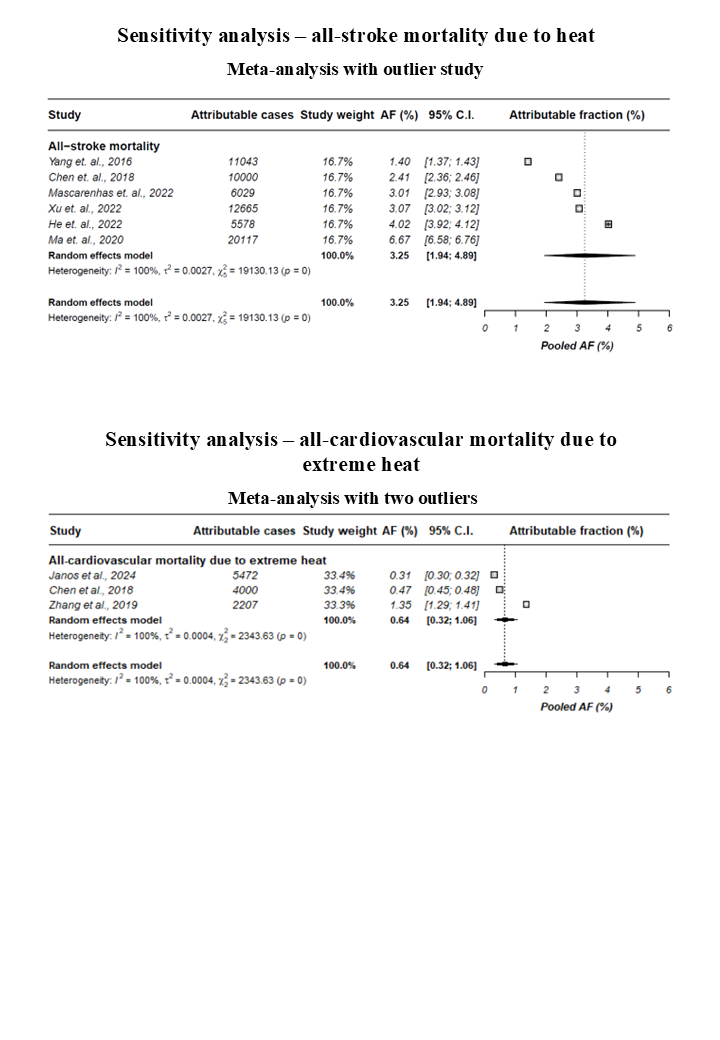


## S12. Funnel plots


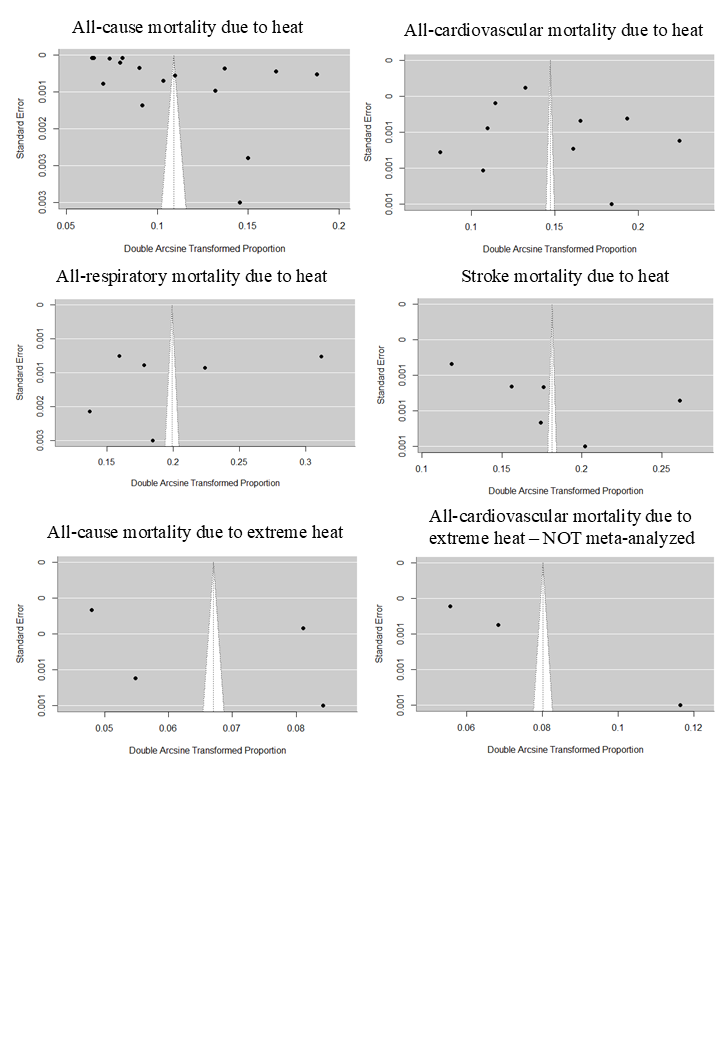


### References of Appendix

1. Aboubakri O, Khanjani N, Jahani Y, Bakhtiari B. Attributable risk of mortality associated with heat and heat waves: A time-series study in Kerman, Iran during 2005–2017. *Journal of Thermal Biology* 2019; **82**: 76-82.

2. Huber V, Krummenauer L, Peña-Ortiz C, et al. Temperature-related excess mortality in German cities at 2 °C and higher degrees of global warming. *Environmental Research* 2020; **186**: 109447.

3. Chen R, Yin P, Wang L, et al. Association between ambient temperature and mortality risk and burden: time series study in 272 main Chinese cities. *Bmj* 2018; **363**: k4306.

4. Ingole V, Sheridan SC, Juvekar S, Achebak H, Moraga P. Mortality risk attributable to high and low ambient temperature in Pune city, India: A time series analysis from 2004 to 2012. *Environmental Research* 2022; **204**: 112304.

5. Zhang Y, Wang SG, Zhang XL, Cheng YF, Tang CJ. Mortality Risk Attributed to Ambient Temperature in Nanjing, China. *Biomed Environ Sci* 2019; **32**(1): 42-6.

6. Xu X, Chen Z, Huo X, et al. The effects of temperature on human mortality in a Chinese city: burden of disease calculation, attributable risk exploration, and vulnerability identification. *International Journal of Biometeorology* 2019; **63**(10): 1319-29.

7. Denpetkul T, Phosri A. Daily ambient temperature and mortality in Thailand: Estimated effects, attributable risks, and effect modifications by greenness. *Science of The Total Environment* 2021; **791**: 148373.

8. Mascarenhas MS, Silva DDD, Nogueira MC, Farias WCM, Ferreira CCM, Ferreira LCM. The effect of air temperature on mortality from cerebrovascular diseases in Brazil between 1996 and 2017. *Cien Saude Colet* 2022; **27**(8): 3295-306.

9. Aboubakri O, Rezaee R, Maleki A, et al. Temporal change in cold and heat-related burden of mortality: an evidence of increasing heat impact in Iran. *Air Quality, Atmosphere & Health* 2023; **16**(12): 2421-9.

10. Sera F, Armstrong B, Tobias A, et al. How urban characteristics affect vulnerability to heat and cold: a multi-country analysis. *Int J Epidemiol* 2019; **48**(4): 1101-12.

11. Gasparrini A, Guo Y, Hashizume M, et al. Mortality risk attributable to high and low ambient temperature: a multicountry observational study. *Lancet* 2015; **386**(9991): 369-75.

12. Martínez-Solanas È, Quijal-Zamorano M, Achebak H, et al. Projections of temperature-attributable mortality in Europe: a time series analysis of 147 contiguous regions in 16 countries. *The Lancet Planetary Health* 2021; **5**(7): e446-e54.

13. Vicedo-Cabrera AM, Sera F, Guo Y, et al. A multi-country analysis on potential adaptive mechanisms to cold and heat in a changing climate. *Environment International* 2018; **111**: 239-46.

14. Yezli S, Khan AH, Yassin YM, Khan AA, Alotaibi BM, Bouchama A. Human tolerance to extreme heat: evidence from a desert climate population. *Journal of Exposure Science & Environmental Epidemiology* 2023; **33**(4): 631-6.

15. Boudreault J, Lavigne É, Campagna C, Chebana F. Estimating the heat-related mortality and morbidity burden in the province of Quebec, Canada. *Environmental Research* 2024; **257**: 119347.

16. Sharma A, Dutta P, Shah P, et al. Characterizing the effects of extreme heat events on all-cause mortality: A case study in Ahmedabad city of India, 2002–2018. *Urban Climate* 2024; **54**: 101832.

17. Pascal M, Wagner V, Corso M. Changes in the temperature-mortality relationship in France: Limited evidence of adaptation to a new climate. *International Journal of Biometeorology* 2023; **67**(4): 725-34.

18. Liu J, Hansen A, Varghese B, et al. Cause-specific mortality attributable to cold and hot ambient temperatures in Hong Kong: a time-series study, 2006–2016. *Sustainable Cities and Society* 2020; **57**: 102131.

19. Pattenden S, Nikiforov B, Armstrong BG. Mortality and temperature in Sofia and London. *J Epidemiol Community Health* 2003; **57**(8): 628-33.

20. Ordanovich D, Tobías A, Ramiro D. Temporal variation of the temperature-mortality association in Spain: a nationwide analysis. *Environmental Health* 2023; **22**(1): 5.

21. Fu SH, Gasparrini A, Rodriguez PS, Jha P. Mortality attributable to hot and cold ambient temperatures in India: a nationally representative case-crossover study. *PLOS Medicine* 2018; **15**(7): e1002619.

22. Janoš T, Ballester J, Čupr P, Achebak H. Countrywide analysis of heat- and cold-related mortality trends in the Czech Republic: growing inequalities under recent climate warming. *International Journal of Epidemiology* 2024; **53**(1): dyad141.

23. Lee W-H, Lim Y-H, Dang TN, et al. An Investigation on Attributes of Ambient Temperature and Diurnal Temperature Range on Mortality in Five East-Asian Countries. *Scientific Reports* 2017; **7**(1): 10207.

24. Su X, Song H, Cheng Y, Yao X, Li Y. The mortality burden of nervous system diseases attributed to ambient temperature: A multi-city study in China. *Science of The Total Environment* 2021; **800**: 149548.

25. Wu Y, Wen B, Li S, et al. Fluctuating temperature modifies heat-mortality association around the globe. *The Innovation* 2022; **3**(2).

26. Wen B, Wu Y, Guo Y, et al. Comparison for the effects of different components of temperature variability on mortality: A multi-country time-series study. *Environment International* 2024; **187**: 108712.

27. Lee W, Bell ML, Gasparrini A, et al. Mortality burden of diurnal temperature range and its temporal changes: A multi-country study. *Environment International* 2018; **110**: 123-30.

28. Lee W, Kim Y, Sera F, et al. Projections of excess mortality related to diurnal temperature range under climate change scenarios: a multi-country modelling study. *The Lancet Planetary Health* 2020; **4**(11): e512-e21.

29. Wang Y, Chen Y, Chen J, et al. Mortality risk attributable to diurnal temperature range: a multicity study in Yunnan of southwest China. *Environmental Science and Pollution Research* 2021; **28**(43): 60597-608.

30. Wen B, Wu Y, Guo Y, Li S. A new method to separate the impacts of interday and intraday temperature variability on mortality. *BMC Medical Research Methodology* 2023; **23**(1): 92.

31. Yu Y, Luo S, Zhang Y, et al. Comparative analysis of daily and hourly temperature variability in association with all-cause and cardiorespiratory mortality in 45 US cities. *Environmental Science and Pollution Research* 2022; **29**(8): 11625-33.

32. Zhang Y, Xiang Q, Yu C, et al. Mortality risk and burden associated with temperature variability in China, United Kingdom and United States: Comparative analysis of daily and hourly exposure metrics. *Environmental Research* 2019; **179**: 108771.

33. Zhang Y, Yu Y, Peng M, Meng R, Hu K, Yu C. Temporal and seasonal variations of mortality burden associated with hourly temperature variability: A nationwide investigation in England and Wales. *Environment International* 2018; **115**: 325-33.

34. Lin H, Liu T, Xiao J, et al. Mortality burden of ambient fine particulate air pollution in six Chinese cities: Results from the Pearl River Delta study. *Environment International* 2016; **96**: 91-7.

35. Hu K, Guo Y, Hu D, et al. Mortality burden attributable to PM1 in Zhejiang province, China. *Environment International* 2018; **121**: 515-22.

36. Zhu Q, Yu M, Bai G, et al. The joint associations of ambient air pollutants and weather factors with mortality: Evidence from a national time-stratified case-crossover study in China. *Science of The Total Environment* 2024; **907**: 168129.

37. Guo J, Zhou J, Han R, et al. Association of Short-Term Co-Exposure to Particulate Matter and Ozone with Mortality Risk. *Environmental Science & Technology* 2023; **57**(42): 15825-34.

38. Chen G, Guo Y, Yue X, et al. All-cause, cardiovascular, and respiratory mortality and wildfire-related ozone: a multicountry two-stage time series analysis. *The Lancet Planetary Health* 2024; **8**(7): e452-e62.

39. Xue T, Wu J, Li F, et al. Variation in under-5 mortality attributable to anomalous precipitation during El Niño–Southern Oscillation cycles: Assessment of the intertemporal inequality in child health. *Global Environmental Change* 2024; **87**: 102879.

40. Yang Z, Huang W, McKenzie JE, et al. Mortality risks associated with floods in 761 communities worldwide: time series study. *BMJ* 2023; **383**: e075081.

41. Li Z, Fan Y, Xu Z, et al. Exceptional heatwaves and mortality in Europe: Greater impacts since the coronavirus disease 2019 outbreak. *Environmental Pollution* 2024; **363**: 125058.

42. Zhao C, Li Y-H, Tong S, et al. Economic burden of premature deaths attributable to different heatwaves in China: A multi-site study, 2014–2019. *Advances in Climate Change Research* 2023; **14**(6): 836-46.

43. Adegboye MA, Olumoh J, Saffary T, Elfaki F, Adegboye OA. Effects of time-lagged meteorological variables on attributable risk of leishmaniasis in central region of Afghanistan. *Science of The Total Environment* 2019; **685**: 533-41.

44. Yi W, Cheng J, Wei Q, et al. Disparities of weather type and geographical location in the impacts of temperature variability on cancer mortality: A multicity case-crossover study in Jiangsu Province, China. *Environmental Research* 2021; **197**: 110985.

45. Zhan Z-Y, Xu X-Y, Wei J, et al. Short-term associations of particulate matter with different aerodynamic diameters with mortality due to mental disorders and dementia in Ningde, China. *Ecotoxicology and Environmental Safety* 2024; **271**: 115931.

46. Zhang R, Sun L, Jia A, et al. Effect of heatwaves on mortality of Alzheimer's disease and other dementias among elderly aged 60 years and above in China, 2013&#x2013;2020: a population-based study. *The Lancet Regional Health – Western Pacific* 2024; **52**.

47. Min J, Kang DH, Kang C, et al. Fluctuating risk of acute kidney injury-related mortality for four weeks after exposure to air pollution: A multi-country time-series study in 6 countries. *Environ Int* 2024; **183**: 108367.

48. Huang Z, Li Z, Hu J, et al. The association of heatwave with drowning mortality in five provinces of China. *Science of The Total Environment* 2023; **903**: 166321.

49. Achebak H, Devolder D, Ballester J. Trends in temperature-related age-specific and sex-specific mortality from cardiovascular diseases in Spain: a national time-series analysis. *The Lancet Planetary Health* 2019; **3**(7): e297-e306.

50. Han L, Sun Z, He J, et al. Estimating the mortality burden attributable to temperature and PM2.5 from the perspective of atmospheric flow. *Environmental Research Letters* 2020; **15**(12): 124059.

51. Xu R, Shi C, Wei J, et al. Cause-specific cardiovascular disease mortality attributable to ambient temperature: A time-stratified case-crossover study in Jiangsu province, China. *Ecotoxicology and Environmental Safety* 2022; **236**: 113498.

52. Yang J, Yin P, Zhou M, et al. Cardiovascular mortality risk attributable to ambient temperature in China. *Heart* 2015; **101**(24): 1966-72.

53. Psistaki K, Kouis P, Michanikou A, Yiallouros PK, Papatheodorou SI, Paschalidou A. Temporal trends in temperature-related mortality and evidence for maladaptation to heat and cold in the Eastern Mediterranean region. *Sci Total Environ* 2024; **943**: 173899.

54. Adegboye OA, McBryde ES, Eisen DP. Epidemiological analysis of association between lagged meteorological variables and pneumonia in wet-dry tropical North Australia, 2006–2016. *Journal of Exposure Science & Environmental Epidemiology* 2020; **30**(3): 448-58.

55. Ferreira LdCM, Nogueira MC, Pereira RVdB, et al. Ambient temperature and mortality due to acute myocardial infarction in Brazil: an ecological study of time-series analyses. *Scientific Reports* 2019; **9**(1): 13790.

56. Hu P, Chang J, Huang Y, et al. Nonoptimum Temperatures Are More Closely Associated With Fatal Myocardial Infarction Than With Nonfatal Events. *Can J Cardiol* 2023; **39**(12): 1974-83.

57. Xu R, Huang S, Shi C, et al. Extreme Temperature Events, Fine Particulate Matter, and Myocardial Infarction Mortality. *Circulation* 2023; **148**(4): 312-23.

58. He F, Wei J, Dong Y, et al. Associations of ambient temperature with mortality for ischemic and hemorrhagic stroke and the modification effects of greenness in Shandong Province, China. *Science of The Total Environment* 2022; **851**: 158046.

59. Yang J, Yin P, Zhou M, et al. The burden of stroke mortality attributable to cold and hot ambient temperatures: Epidemiological evidence from China. *Environ Int* 2016; **92-93**: 232-8.

60. Zhou Y, Pan J, Xu R, et al. Asthma mortality attributable to ambient temperatures: A case-crossover study in China. *Environmental Research* 2022; **214**: 114116.

61. Iñiguez C, Royé D, Tobías A. Contrasting patterns of temperature related mortality and hospitalization by cardiovascular and respiratory diseases in 52 Spanish cities. *Environmental Research* 2021; **192**: 110191.

62. Tang W, Yu M, Bai G, et al. Ambient ozone and mortality from respiratory diseases: A nationwide analysis in China. *Global Transitions* 2024; **6**: 113-22.

63. Thawonmas R, Kim Y, Hashizume M. Short-term exposure to ambient temperature and the mortality burden of suicide in Japan. *Environmental Research Communications* 2024; **6**(6): 065012.

64. Wu Q, Xing X, Yang M, et al. Increased Suicide Mortality and Reduced Life Expectancy Associated With Ambient Heat Exposure. *American Journal of Preventive Medicine* 2024; **66**(5): 780-8.

65. Tong MX, Wondmagegn BY, Xiang J, et al. Emergency department visits and associated healthcare costs attributable to increasing temperature in the context of climate change in Perth, Western Australia, 2012–2019. *Environmental Research Letters* 2021; **16**(6): 065011.

66. Wu W, Chen B, Wu G, et al. Increased susceptibility to temperature variation for non-accidental emergency ambulance dispatches in Shenzhen, China. *Environmental Science and Pollution Research* 2021; **28**(24): 32046-56.

67. Schulte F, Röösli M, Ragettli MS. Risk, Attributable Fraction and Attributable Number of Cause-Specific Heat-Related Emergency Hospital Admissions in Switzerland. *International Journal of Public Health* 2024; **Volume 69 - 2024**.

68. Wen B, Kliengchuay W, Suwanmanee S, et al. Association of cause-specific hospital admissions with high and low temperatures in Thailand: a nationwide time series study. *The Lancet Regional Health – Western Pacific* 2024; **46**.

69. Zhao Y, Huang Z, Wang S, et al. Morbidity burden of respiratory diseases attributable to ambient temperature: a case study in a subtropical city in China. *Environmental Health* 2019; **18**(1): 89.

70. Zhao Q, Coelho MSZS, Li S, et al. Spatiotemporal and demographic variation in the association between temperature variability and hospitalizations in Brazil during 2000–2015: A nationwide time-series study. *Environment International* 2018; **120**: 345-53.

71. Huang Y-S, Song H-J, Cheng Y-B, et al. Temperature change between neighboring days and hospital admissions in China. *Advances in Climate Change Research* 2023; **14**(6): 847-55.

72. Jiao A, Xiang Q, Ding Z, et al. Short-term impacts of ambient fine particulate matter on emergency department visits: Comparative analysis of three exposure metrics. *Chemosphere* 2020; **241**: 125012.

73. Xu J, Chen Y, Lu F, Chen L, Dong Z. The Association between Short-Term Exposure to PM1 and Daily Hospital Admission and Related Expenditures in Beijing. *Toxics*, 2024. (accessed.

74. Ye T, Guo Y, Chen G, et al. Risk and burden of hospital admissions associated with wildfire-related PM<sub>2&#xb7;5</sub> in Brazil, 2000&#x2013;15: a nationwide time-series study. *The Lancet Planetary Health* 2021; **5**(9): e599-e607.

75. Singh N, Mall RK, Banerjee T, Gupta A. Association between climate and infectious diseases among children in Varanasi city, India: A prospective cohort study. *Science of The Total Environment* 2021; **796**: 148769.

76. Wang H, Jiang B, Zhao Q, Zhou C, Ma W. Temperature extremes and infectious diarrhea in China: attributable risks and effect modification of urban characteristics. *International Journal of Biometeorology* 2023; **67**(10): 1659-68.

77. Huang K, Yang X-J, Hu C-Y, et al. Short-term effect of ambient temperature change on the risk of tuberculosis admissions: Assessments of two exposure metrics. *Environmental Research* 2020; **189**: 109900.

78. Lan T, Hu Y, Cheng L, et al. Floods and diarrheal morbidity: Evidence on the relationship, effect modifiers, and attributable risk from Sichuan Province, China. *J Glob Health* 2022; **12**: 11007.

79. Ma Y, Wen T, Xing D, Zhang Y. Associations between floods and bacillary dysentery cases in main urban areas of Chongqing, China, 2005–2016: a retrospective study. *Environmental Health and Preventive Medicine* 2021; **26**(1): 49.

80. Kang N, Wang R, Lu H, et al. Burden of Child Anemia Attributable to Fine Particulate Matters Brought by Sand Dusts in Low- and Middle-Income Countries. *Environ Sci Technol* 2024; **58**(29): 12954-65.

81. Qiu H, Wang L, Zhou L, Pan J. Coarse particles (PM2.5-10) and cause-specific hospitalizations in southwestern China: Association, attributable risk and economic costs. *Environmental Research* 2020; **190**: 110004.

82. Jiang W, Chen H, Li H, et al. The Short-Term Effects and Burden of Ambient Air Pollution on Hospitalization for Type 2 Diabetes: Time-Stratified Case-Crossover Evidence From Sichuan, China. *Geohealth* 2023; **7**(11): e2023GH000846.

83. Zhang Y, Xu R, Huang W, et al. Short-term Exposure to Wildfire-Specific PM2.5 and Diabetes Hospitalization: A Study in Multiple Countries and Territories. *Diabetes Care* 2024; **47**(9): 1664-72.

84. Pan R, Zhang X, Gao J, et al. Impacts of heat and cold on hospitalizations for schizophrenia in Hefei, China: An assessment of disease burden. *Science of The Total Environment* 2019; **694**: 133582.

85. Crank PJ, Hondula DM, Sailor DJ. Mental health and air temperature: Attributable risk analysis for schizophrenia hospital admissions in arid urban climates. *Science of The Total Environment* 2023; **862**: 160599.

86. Corvetto JF, Helou AY, Kriit HK, et al. Private vs. public emergency visits for mental health due to heat: An indirect socioeconomic assessment of heat vulnerability and healthcare access, in Curitiba, Brazil. *Sci Total Environ* 2024; **934**: 173312.

87. Yi W, Zhang X, Pan R, et al. Quantifying the impacts of temperature variability on hospitalizations for schizophrenia: A time series analysis in Hefei, China. *Science of The Total Environment* 2019; **696**: 133927.

88. Qiu H, Zhu X, Wang L, et al. Attributable risk of hospital admissions for overall and specific mental disorders due to particulate matter pollution: A time-series study in Chengdu, China. *Environmental Research* 2019; **170**: 230-7.

89. Ji Y, Liu B, Song J, et al. Short-term effects and economic burden assessment of ambient air pollution on hospitalizations for schizophrenia. *Environmental Science and Pollution Research* 2022; **29**(30): 45449-60.

90. Bai L, Jiang Y, Wang K, et al. Ambient Air Pollution and Hospitalizations for Schizophrenia in China. *JAMA Network Open* 2024; **7**(10): e2436915-e.

91. Xu R, Luo L, Yuan T, et al. Association of short-term exposure to ambient fine particulate matter and ozone with outpatient visits for anxiety disorders: A hospital-based case-crossover study in South China. *J Affect Disord* 2024; **361**: 277-84.

92. Wang X, Yu C, Zhang Y, Shi F, Meng R, Yu Y. Attributable Risk and Economic Cost of Cardiovascular Hospital Admissions Due to Ambient Particulate Matter in Wuhan, China. *Int J Environ Res Public Health* 2020; **17**(15).

93. Liu J, Yu W, Pan R, et al. Association between sequential extreme precipitation-heatwaves events and hospitalizations for schizophrenia: The damage amplification effects of sequential extremes. *Environmental Research* 2022; **214**: 114143.

94. Jiang G, Ji Y, Chen C, et al. Effects of extreme precipitation on hospital visit risk and disease burden of depression in Suzhou, China. *BMC Public Health* 2022; **22**(1): 1710.

95. Wen B, Xu R, Wu Y, et al. Association between ambient temperature and hospitalization for renal diseases in Brazil during 2000–2015: A nationwide case-crossover study. *The Lancet Regional Health - Americas* 2022; **6**: 100101.

96. Chen J, Liu H, Li G, et al. Ambient Air Particulate Matter and Hospital Admissions for Chronic Kidney Disease in China: A Nationwide Case-Crossover Study. *Environment & Health* 2024; **2**(8): 553-62.

97. López-Bueno JA, Díaz J, Padrón-Monedero A, Martín MAN, Linares C. Short-term impact of extreme temperatures, relative humidity and air pollution on emergency hospital admissions due to kidney disease and kidney-related conditions in the Greater Madrid area (Spain). *Sci Total Environ* 2023; **903**: 166646.

98. Zhao C, Huang Y, Cheng Y, et al. Association between heatwaves and risk and economic burden of injury related hospitalizations in China. *Environ Res* 2024; **259**: 119509.

99. Huang Y, Song H, Cheng Y, Bi P, Li Y, Yao X. Heatwave and urinary hospital admissions in China: Disease burden and associated economic loss, 2014 to 2019. *Science of The Total Environment* 2023; **857**: 159565.

100. Wang B, Chai G, Sha Y, Su Y. Association between ambient temperature and cardiovascular disease hospitalisations among farmers in suburban northwest China. *International Journal of Biometeorology* 2022; **66**(7): 1317-27.

101. Onozuka D, Hagihara A. Out-of-hospital cardiac arrest risk attributable to temperature in Japan. *Scientific Reports* 2017; **7**(1): 39538.

102. Park C, Yang J, Lee W, Kang C, Song I-K, Kim H. Excess out-of-hospital cardiac arrests due to ambient temperatures in South Korea from 2008 to 2018. *Environmental Research* 2022; **212**: 113130.

103. Hu P, Chang J, Huang Y, et al. Nonoptimum Temperatures Are More Closely Associated With Fatal Myocardial Infarction Than With Nonfatal Events. *Canadian Journal of Cardiology* 2023; **39**(12): 1974-83.

104. Ji Y, Yuan Z, Huang Z, Xiong J, Li L. Ambient air pollution and hospital admissions for acute aortic dissection in Shantou, China: A time-series analysis. *Atmospheric Environment* 2024; **318**: 120272.

105. Wei Y, Fei L, Wang Y, et al. A time-series analysis of short-term ambient ozone exposure and hospitalizations from acute myocardial infarction in Henan, China. *Environ Sci Pollut Res Int* 2023; **30**(40): 93242-54.

106. Chen Y, Chang Zg, Zhao Y, et al. Association of extreme precipitation with hospitalizations for acute myocardial infarction in Beijing, China: A time-series study. *Frontiers in Public Health* 2022; **10**.

107. Bao J, Guo Y, Wang Q, et al. Effects of heat on first-ever strokes and the effect modification of atmospheric pressure: A time-series study in Shenzhen, China. *Science of The Total Environment* 2019; **654**: 1372-8.

108. Lei L, Bao J, Guo Y, Wang Q, Peng J, Huang C. Effects of diurnal temperature range on first-ever strokes in different seasons: a time-series study in Shenzhen, China. *BMJ Open* 2020; **10**: e033571.

109. Wu H, Zhang B, Wei J, et al. Short-term effects of exposure to ambient PM1, PM2.5, and PM10 on ischemic and hemorrhagic stroke incidence in Shandong Province, China. *Environmental Research* 2022; **212**: 113350.

110. Fang J, Song J, Wu R, et al. Association between ambient temperature and childhood respiratory hospital visits in Beijing, China: a time-series study (2013–2017). *Environmental Science and Pollution Research* 2021; **28**(23): 29445-54.

111. Qiu H, Sun S, Tang R, Chan K-P, Tian L. Pneumonia Hospitalization Risk in the Elderly Attributable to Cold and Hot Temperatures in Hong Kong, China. *American Journal of Epidemiology* 2016; **184**(8): 570-8.

112. Nguyen VT, Doan Q-V, Tran NN, et al. The protective effect of green space on heat-related respiratory hospitalization among children under 5 years of age in Hanoi, Vietnam. *Environmental Science and Pollution Research* 2022; **29**(49): 74197-207.

113. Jiang F, Wang R, Yang Y, et al. Effects of intra- and inter-day temperature change on acute upper respiratory infections among college students, assessments of three temperature change indicators. *Front Public Health* 2024; **12**: 1406415.

114. Zhai C, Bai L, Xu Y, et al. Temperature variability associated with respiratory disease hospitalisations, hospital stays and hospital expenses the warm temperate sub-humid monsoon climate. *Public Health* 2023; **225**: 206-17.

115. Zhan ZY, Tian Q, Chen TT, et al. Temperature Variability and Hospital Admissions for Chronic Obstructive Pulmonary Disease: Analysis of Attributable Disease Burden and Vulnerable Subpopulation. *Int J Chron Obstruct Pulmon Dis* 2020; **15**: 2225-35.

116. Dąbrowiecki P, Chciałowski A, Dąbrowiecka A, Badyda A. Ambient Air Pollution and Risk of Admission Due to Asthma in the Three Largest Urban Agglomerations in Poland: A Time-Stratified, Case-Crossover Study. *International Journal of Environmental Research and Public Health* 2022; **19**(10): 5988.

117. Zhang R, Li Y, Bi P, et al. Seasonal associations between air pollutants and influenza in 10 cities of southern China. *International Journal of Hygiene and Environmental Health* 2023; **252**: 114200.

118. Qu F, Liu F, Zhang H, et al. The hospitalization attributable burden of acute exacerbations of chronic obstructive pulmonary disease due to ambient air pollution in Shijiazhuang, China. *Environmental Science and Pollution Research* 2019; **26**(30): 30866-75.

119. Qiu H, Tan K, Long F, et al. The Burden of COPD Morbidity Attributable to the Interaction between Ambient Air Pollution and Temperature in Chengdu, China. *Int J Environ Res Public Health* 2018; **15**(3).

120. Nguyen TTN, Vu TD, Vuong NL, et al. Effect of ambient air pollution on hospital admission for respiratory diseases in Hanoi children during 2007-2019. *Environ Res* 2024; **241**: 117633.

121. Zhou E, Zhou B, Zhang L, et al. The effect and burden of sand-dust storms on asthma hospitalization: Evidence from cities with arid climate in China. *Environ Res* 2025; **264**(Pt 1): 120345.

122. Chen Z, Li M, Lan T, et al. Effects of ambient temperature on atopic dermatitis and attributable health burden: a 6-year time-series study in Chengdu, China. *PeerJ* 2023; **11**: e15209.

123. Jiang Y, Li G, Wu S, Duan F, Liu S, Liu Y. Assessment of short-term effects of ambient air pollution exposure on osteoarthritis outpatient visits. *Ecotoxicology and Environmental Safety* 2024; **284**: 117014.

124. Martínez-Solanas È, López-Ruiz M, Wellenius Gregory A, et al. Evaluation of the Impact of Ambient Temperatures on Occupational Injuries in Spain. *Environmental Health Perspectives* 2018; **126**(6): 067002.

125. Fatima SH, Giles LC, Rothmore P, Varghese BM, Bi P. Heatwaves and occupational injuries and illnesses risk varied at localised spatial scale: A national study in Australia. *Safety Science* 2025; **181**: 106684.

126. Borg MA, Xiang J, Anikeeva O, et al. Current and projected heatwave-attributable occupational injuries, illnesses, and associated economic burden in Australia. *Environ Res* 2023; **236**(Pt 2): 116852.

127. Qiu J, Liang Z, Yi J, et al. Extreme temperature exposure increases the risk of preterm birth in women with abnormal pre-pregnancy body mass index: a cohort study in a southern province of China. *Front Public Health* 2023; **11**: 1156880.

128. He Y-S, Wu Z-D, Wang G-H, et al. Impact of short-term exposure to ambient air pollution on osteoarthritis: a multi-city time-series analysis in Central-Eastern China. *Environmental Science and Pollution Research* 2023; **30**(47): 104258-69.

129. Allen RW, Gombojav E, Barkhasragchaa B, et al. An assessment of air pollution and its attributable mortality in Ulaanbaatar, Mongolia. *Air Qual Atmos Health* 2013; **6**(1): 137-50.

130. Wang H, Qian G, Shi J, et al. Association between short-term exposure to ambient air pollution and upper respiratory tract infection in Kunshan. *Int J Biometeorol* 2024; **68**(2): 189-97.

131. He Y, Gao Z, Guo T, et al. Fine particulate matter associated mortality burden of lung cancer in Hebei Province, China. *Thorac Cancer* 2018; **9**(7): 820-6.

132. Yu P, Xu R, Coelho MSZS, et al. The impacts of long-term exposure to PM2.5 on cancer hospitalizations in Brazil. *Environment International* 2021; **154**: 106671.

133. Wu ZD, Chen C, He YS, et al. Association between air pollution exposure and outpatient visits for dermatomyositis in a humid subtropical region of China: a time-series study. *Environ Geochem Health* 2023; **45**(8): 6095-107.

134. Li YH, Tong Tan J, Hwa Ooi P, Jiang F, Kan H, Leung WK. Association Between Short-Term Exposure to Air Pollutants and Emergency Attendance for Upper Gastrointestinal Bleeding in Hong Kong: A Time-Series Study. *Geohealth* 2024; **8**(11): e2024GH001086.

135. Rahmati S, Aboubakri O, Maleki A, et al. Risk of cardiovascular and respiratory diseases attributed to satellite-based PM2.5 over 2017–2022 in Sanandaj, an area of Iran. *International Journal of Biometeorology* 2024; **68**(8): 1689-98.

136. Sepandi M, Akbari H, Naseri MH, Alimohamadi Y. Emergency hospital admissions for cardiovascular diseases attributed to air pollution in Tehran during 2016-2019. *Environmental Science and Pollution Research* 2021; **28**(28): 38426-33.

137. Gonçalves KDS, Cirino GG, Costa MOD, Couto LOD, Tortelote GG, Hacon SS. The potential impact of PM2.5 on the covid-19 crisis in the Brazilian Amazon region. *Rev Saude Publica* 2023; **57**: 67.

138. Wang J, Li W, Huang W, et al. The associations of ambient fine particles with tuberculosis incidence and the modification effects of ambient temperature: A nationwide time-series study in China. *J Hazard Mater* 2023; **460**: 132448.

139. Zhang Y, Ye T, Yu P, et al. Preterm birth and term low birth weight associated with wildfire-specific PM2.5: A cohort study in New South Wales, Australia during 2016–2019. *Environment International* 2023; **174**: 107879.

140. Li Y-H, Ye D-X, Liu Y, et al. Association of heat exposure and emergency ambulance calls: A multi-city study. *Advances in Climate Change Research* 2021; **12**(5): 619-27.

141. Ascaso MS, Díaz J, López-Bueno JA, Navas MA, Mirón IJ, Linares C. How heatwaves affect short-term emergency hospital admissions due to bacterial foodborne diseases. *Sci Total Environ* 2024; **946**: 174209.

142. Xu R, Zhao Q, Coelho Micheline SZS, et al. Association between Heat Exposure and Hospitalization for Diabetes in Brazil during 2000–2015: A Nationwide Case-Crossover Study. *Environmental Health Perspectives* 2019; **127**(11): 117005.

143. Yang C-L, Bao J-Z, Bi P, Zhang Y-D, Tan C-M, Chen K. Association between heat and upper urinary tract stones morbidity and medical costs: A study in the subtropical humid climate zone. *Advances in Climate Change Research* 2024; **15**(4): 717-24.

144. Zhang R, Lin Z, Guo Z, et al. Daily mean temperature and HFMD: risk assessment and attributable fraction identification in Ningbo China. *Journal of Exposure Science & Environmental Epidemiology* 2021; **31**(4): 664-71.

145. Su Y, Cheng L, Cai W, et al. Evaluating the effectiveness of labor protection policy on occupational injuries caused by extreme heat in a large subtropical city of China. *Environmental Research* 2020; **186**: 109532.

146. Tao J, Zhang Y, Li Z, et al. Daytime and nighttime high temperatures differentially increased the risk of cardiovascular disease: A nationwide hospital-based study in China. *Environ Res* 2023; **236**(Pt 1): 116740.

147. Feng Y, Xu Z, Zahid Hossain M, et al. Impact of hot and cold nights on pneumonia hospitalisations in children under five years: Evidence from low-, middle-, and high-income countries. *Environ Int* 2024; **192**: 109041.

148. Tao J, Zhang Y, Wu Q, et al. Impacts of hot and cold nights on mental disorders: A three-stage nationwide hospital-based time-series study with 1.2 million patients in China. *Global Transitions* 2024; **6**: 10-8.

149. Jingesi M, Yin Z, Huang S, et al. Cardiovascular morbidity risk attributable to thermal stress: analysis of emergency ambulance dispatch data from Shenzhen, China. *BMC Public Health* 2024; **24**(1): 2861.

150. Zhou L, Chen R, He C, et al. Ambient heat stress and urolithiasis attacks in China: Implication for climate change. *Environ Res* 2023; **217**: 114850.

151. Zhao Q, Coelho MSZS, Li S, et al. Temperature variability and hospitalization for cardiac arrhythmia in Brazil: A nationwide case-crossover study during 2000–2015. *Environmental Pollution* 2019; **246**: 552-8.

152. Yang C, Li S, Yang Y, et al. Heatwave and upper urinary tract stones morbidity: effect modification by heatwave definitions, disease subtypes, and vulnerable populations. *Urolithiasis* 2024; **52**(1): 134.

153. Lin Z, Weinberger E, Nori-Sarma A, Chinchilla M, Wellenius GA, Jay J. Daily heat and mortality among people experiencing homelessness in 2 urban US counties, 2015-2022. *Am J Epidemiol* 2024; **193**(11): 1576-82.

154. Jiang Z, Lin Z, Li Z, et al. Joint effects of heat-humidity compound events on drowning mortality in Southern China. *Inj Prev* 2024; **30**(6): 488-95.

155. Zhang H, Li X, Shang W, et al. Risk and attributable fraction estimation for the impact of exposure to compound drought and hot events on daily stroke admissions. *Environ Health Prev Med* 2024; **29**: 56.

156. Vicedo-Cabrera AM, Scovronick N, Sera F, et al. The burden of heat-related mortality attributable to recent human-induced climate change. *Nature Climate Change* 2021; **11**(6): 492-500.

157. Bernstein AS, Sun S, Weinberger KR, Spangler KR, Sheffield PE, Wellenius GA. Warm Season and Emergency Department Visits to U.S. Children's Hospitals. *Environ Health Perspect* 2022; **130**(1): 17001.

158. Cheng J, Xie MY, Zhao KF, et al. Impacts of ambient temperature on the burden of bacillary dysentery in urban and rural Hefei, China. *Epidemiol Infect* 2017; **145**(8): 1567-76.

159. Xu R, Zhao Q, Coelho MSZS, et al. The association between heat exposure and hospitalization for undernutrition in Brazil during 2000−2015: A nationwide case-crossover study. *PLOS Medicine* 2019; **16**(10): e1002950.

160. Liu F, Qu F, Zhang H, et al. The effect and burden modification of heating on adult asthma hospitalizations in Shijiazhuang: a time-series analysis. *Respiratory Research* 2019; **20**(1): 122.

161. Yan S, Wang X, Yao Z, et al. Seasonal characteristics of temperature variability impacts on childhood asthma hospitalization in Hefei, China: Does PM2.5 modify the association? *Environmental Research* 2022; **207**: 112078.

162. Lu P, Xia G, Zhao Q, et al. Attributable risks of hospitalizations for urologic diseases due to heat exposure in Queensland, Australia, 1995-2016. *Int J Epidemiol* 2022; **51**(1): 144-54.

163. Wang F-L, Wang W-Z, Zhang F-F, et al. Heat exposure and hospitalizations for chronic kidney disease in China: a nationwide time series study in 261 major Chinese cities. *Military Medical Research* 2023; **10**(1): 41.

164. He Q, Liu Y, Yin P, et al. Differentiating the impacts of ambient temperature on pneumonia mortality of various infectious causes: a nationwide, individual-level, case-crossover study. *EBioMedicine* 2023; **98**: 104854.
